# Electrostatic Fields Induce Accelerated Proton Coupled Electron Transfer Rates in Chlorophyll Model Compounds

**DOI:** 10.1021/jacs.5c09511

**Published:** 2025-07-29

**Authors:** Oscar Reid Kelly, Brendan Twamley, Marcel Swart, Aidan R. McDonald

**Affiliations:** † School of Chemistry, Trinity College Dublin, The University of Dublin, College Green, Dublin 2, Ireland; ‡ IQCC and Department of Chemistry, 16738 Universitat de Girona, Girona 17003, Spain; § ICREA, Pg. Lluís Companys 23, Barcelona 08010, Spain

## Abstract

Chlorophyll-based pigments are crucial mediators of redox processes in photosynthesis, serving as the primary electron donors in photosystems I and II. Despite their structural similarities, these pigments exhibit a wide range of redox potentials (0.5–1.3 V vs SHE), and little experimental insight into the origins of this variation is available. To address this deficit, we have synthesized two crown ether-appended Mg-porphyrin complexes as chlorophyll model compounds and demonstrated their ability to bind redox-inactive metal cations. Cation binding to the Mg-porphyrin complexes was found to increase their redox potentials in a manner that depends linearly on the total cationic charge felt by the complex, implicating a through-space electrostatic field effect. The corresponding 1-electron oxidized π-cation radical complexes were then prepared and characterized by UV–vis, FT-IR, and EPR spectroscopies and ESI-MS. The π-cation radical species were found to be competent for the PCET oxidation of a phenolic substrate, mimicking the reaction between photo-oxidized chlorophyll and tyrosine in photosystem II. Cation binding to the π-cation radical complexes was found to increase the rates of their PCET and ET reactions in a charge-dependent manner which could be rationalized using Marcus theory. This work provides direct experimental evidence that electrostatic fields can tune the redox potentials of chlorophyll model compounds, leading to an increase in their oxidative reactivity.

## Graphical abstract

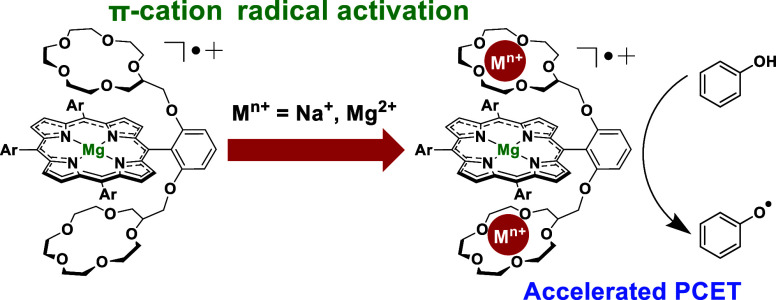

## Introduction

Chlorophyll is an essential component of the photosynthetic machinery of cyanobacteria, algae, and higher plants, being responsible for both light-harvesting and electron transport.^1–3^ For example, multimeric chlorophyll pigments called P700 and P680 serve as the primary electron donors of photosystems I and II (PSI and PSII), respectively.^1,3^ Light energy is concentrated in the reaction centre (RC) of PSII (the locus of water oxidation) leading to the photo-oxidation of P680 (λ_max_ = 680 nm) and release of an electron into the photosynthetic electron transport chain. This electron is eventually transferred to photoexcited P700 (λ_max_ = 700 nm), which continues the transfer of electrons to provide the reducing equivalents for carbon fixation elsewhere in the plant.^2,4^ The species generated by photo-oxidation of P680 (P680^+^) is postulated to be a Mg-chlorin π-cation radical species with an estimated redox potential of 1.1–1.3 V vs the standard hydrogen electrode (SHE). P680^+^ oxidizes the oxygen evolving complex (OEC, a tetramanganese cluster responsible for O_2_ synthesis from H_2_O) via oxidation of a proximal tyrosine residue through a proton coupled electron transfer (PCET) mechanism.^3,5^ P680^+^ is therefore responsible for generating the electrochemical driving force required for photosynthetic water oxidation.

Despite the importance of electron transfer by chlorophyll-based pigments in photosynthesis, little is known about the factors that influence the redox potentials of chlorophylls in vivo; despite their similar structural features, the redox potentials of isolated chlorophyll-*a* (0.78 V vs SHE), P700 (0.50 V), and P680 (1.1–1.3 V) span up to 800 mV.^2,6^ There are a number of postulates that attempt to explain this variation, predominantly supported by theoretical calculations: subtle aspects of the spatial relationship between the constituent chlorophyll molecules in P700/P680;^7–10^ charge delocalization in the oxidized state;^11–13^ the nature and positioning of the axial ligands at Mg;^14–16^ and the electrostatic/dielectric environment imposed on the pigments by the surrounding protein.^12,17–19^ However, there is currently a dearth of direct experimental evidence to support these postulates and therefore to explain the wide variation in the redox potentials of these pigments.

Efforts have been made to incorporate internal electrostatic fields into metal complexes to tune their properties outside of the steric-electronic paradigm that has historically dominated the rational design of molecular catalysts.^20–22^ Inspiration for this work largely draws on biology, where electrostatic fields have long been implicated in the efficient catalysis of various enzymes.^23,24^ Most relevant to the present work are cationic metalloporphyrin complexes, which share the same porphyrin core that is used herein to model the Mg-chlorin core of chlorophyll. Electrostatic field effects have been invoked in the study of cationic transition metal porphyrin complexes in the context of electrochemical O_2_ and CO_2_ reduction, where the enhancement of catalytic activity has been attributed to stabilization of charged intermediates during catalysis and enhanced substrate binding.^25–28^ Inductive effects have been implicated in cationic Fe-porphyrin and Fe-porphyrazine complexes in the context of C–H oxygenation and hydrogen atom transfer (HAT), respectively.^29,30^ The highly reactive nature of these species was later suggested to be attributable to electrostatic field effects through computational analysis.^31^ In general, an explicit relation between the redox potentials of these metalloporphyrins and the ligand electrostatic field has not been drawn due to the strongly electron withdrawing effect imposed by their cationic substituents, making the contributions from inductive effects and electrostatic effects difficult to parse. Further, the reactivity exhibited by these compounds is dominated by the central transition metal ion, leaving explicitly ligand-centered reactivity (as relevant to chlorophyll) unexplored.

An attractive strategy to explicitly evaluate the impacts of electrostatic fields on metal complexes involves incorporating secondary binding sites for redox-inactive metals into the ligand framework. This approach has been applied to a number of transition metal complexes, allowing for their redox and reactivity properties to be tuned through the binding of cations.^32–47^ Besides imposing an electrostatic potential on the transition metal ions, the impacts of secondary cation binding in these systems has been interpreted in a number of ways, such as cation-induced reductions in donor ligand strength and structural deformations.^45,47–50^ While such cation-induced effects have been demonstrated to influence the redox/reactivity properties of d- and f-block metals, reports on the impacts of electrostatic fields on complexes of redox-inactive metals, where redox chemistry is borne exclusively by the ligand, are unprecedented to the best of our knowledge.

Synthetic mimics have succeeded in providing insight into some features of P680^+^. For example, freebase porphyrins decorated with phenols and organic bases as covalently bound substituents have allowed for spectroscopic and kinetic studies of charge separation and interrogation of the impact of specific structural features (e.g., H-bonds) on the nature of the radical species formed upon photo-oxidation and PCET.^51–53^ We recently reported the reactivity of a dimeric Mg-porphyrin π-cation radical species which served as a structural and functional mimic of P680^+^, revealing spin delocalization over multiple porphyrins to enhance the rate of PCET through a comparison with the monomeric counterpart.^54^ However, these studies do not address the impacts of electrostatic fields on the PCET chemistry in P680^+^. Herein, we address the role of electrostatic fields on the redox and reactivity properties of chlorophylls through the preparation of synthetic Mg-porphyrin model complexes. We targeted crown ether-substituted porphyrins for this study due to the documented ability of crown ether substituents to bind cations and their synthetic accessibility.^55^ We report on the impacts of mono- and divalent cation binding on the redox and reactivity properties of crown ether-appended Mg-porphyrin complexes and their corresponding π-cation radicals. These compounds serve as structural mimics of chlorophyll and functional mimics of P680/P680^+^ and thus provide insight into the effects of electrostatic fields on photosynthetic electron transport.

## Results and Discussion

### Synthesis and Characterization of Mg-Porphyrin Complexes

Ligand **L1** was accessed by etherification of 5-(2-hydroxyphenyl)-10,15,20-tri-*p*-tolylporphyrin with 2-(tosyloxymethyl)-15-crown-5 according to the method developed by Kuś and co-workers (Scheme 1).^56^ **L2** was prepared from 5-(2,6-dihydroxyphenyl)-10,15,20-tri-*p*-tolylporphyrin under the same conditions (see Supporting Information file for details). The stereoisomers of **L1** and **L2** (arising from the chirality of the crown ether starting material) and their respective Mg^2+^ complexes (vide infra) were treated together throughout the present work (i.e., they were not separated). The ^1^H nuclear magnetic resonance (NMR) spectrum of **L1** showed the methine proton of the crown ether substituent as a broad triplet at δ = 2.87 ppm integrating to one proton, with the remaining crown ether resonances appearing as overlapping peaks between δ = 2.69–2.19 ppm integrating to 18 protons (Figure S1). The methylene resonance of the linker between the porphyrin core and crown ether substituent was found as a broad doublet at δ = 3.92 ppm integrating to two protons. As expected, **L2** exhibited twice the number of crown ether resonances as **L1** in its ^1^H NMR spectrum but otherwise displayed similar ^1^H NMR features (Figure S2). The Fourier transform infrared (FT-IR) spectra of **L1** and **L2** showed sharp but low-intensity features at ν = 3318 cm^–1^ and ν = 3317 cm^–1^, respectively, which were assigned to the pyrrolic N–H groups (Figures S3 and S4). Electrospray-ionization mass spectrometry (ESI-MS) of **L1** showed peaks corresponding to the [**L1** + Na]^+^ cation (calculated *m*/*z* = 927.4092, found *m*/*z* = 927.4082, Figure S5), while the ESI-MS of **L2** showed peaks that were assigned to the [**L2** + 2Na]^2+^, [**L2** + H]^+^ and [**L2** + Na]^+^ cations (calculated *m*/*z* = 599.2622, 1153.5533, 1175.5352, found *m*/*z* = 599.2614, 1153.5538, 1175.5353 respectively, Figure S6). The electronic absorption spectra of **L1** and **L2** were identical and typical of free-base porphyrins, with an intense Soret band at λ = 416 nm and four Q-bands of lesser intensity at λ = 513, 548, 590, and 646 nm arising from π–π* transitions (Figure S7).^57–59^ The similarity of the two spectra indicated that the differing degree of crown-substitution of the ligands had a minimal effect on their electronic structures.

**Scheme 1:**
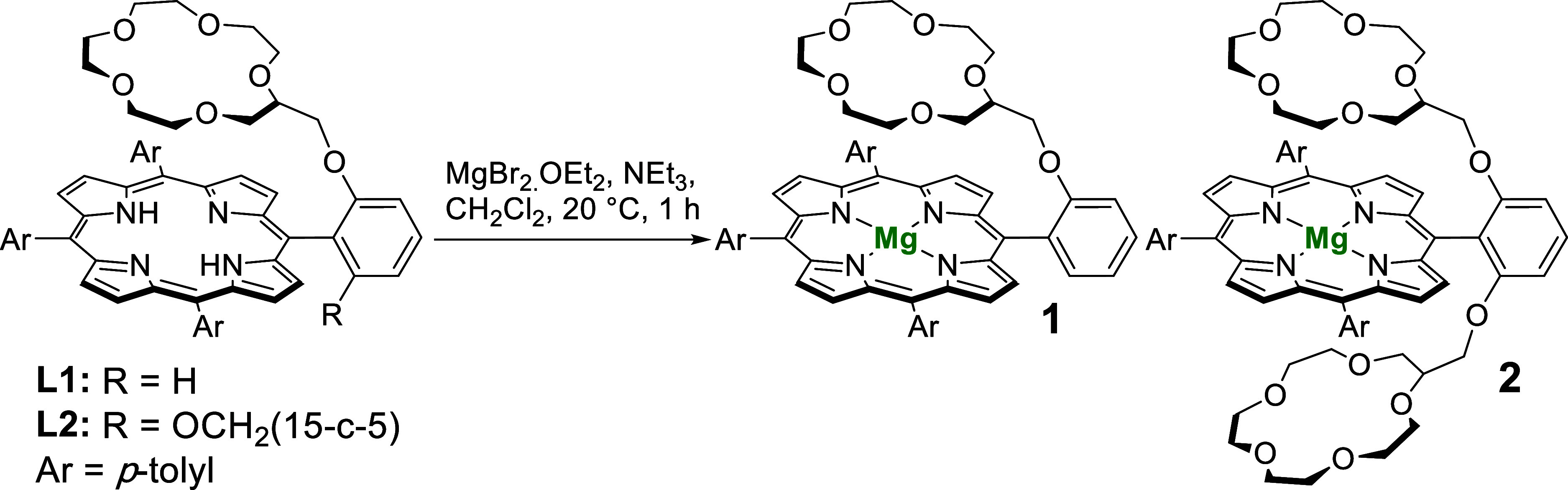
Synthesis of Compounds **1** and **2** ^a^

a OEt_2_ = diethyl ether, NEt_3_ = triethylamine.

Metalation of **L1** and **L2** with Mg^2+^ was achieved by treatment of the ligands with MgBr_2_·OEt_2_ (OEt_2_ = diethyl ether) and triethylamine (NEt_3_) in CH_2_Cl_2_ at 20 °C according to the method of Lindsey and Woodford (Scheme 1).^60^ To assess the impact of the crown ether substituents on the Mg-porphyrin unit, the Mg^2+^ complex of 5,10,15,20-tetra­(*p*-tolyl)­porphyrin ([Mg­(H_2_O)­(TTP)]) was also synthesized by the same method. Mg complexes [Mg­(H_2_O)­(TTP)], **1**, and **2**, which bear zero, one and two covalently bound 15-crown-5 substituents respectively, were isolated in good yields.

Characterization data for [Mg­(H_2_O)­(TTP)] were consistent with literature reports.^61^ The ^1^H NMR spectra of **1** and **2** were consistent with Mg^2+^ insertion into free base ligands, showing the disappearance of the NH resonances of **L1** and **L2** at δ = −2.74 and −2.75 ppm, respectively (Figures S8 and S9). FT-IR also supported metalation of the ligands with the disappearance of the ν_N–H_ of the free-base porphyrins and the appearance of broad peaks at ν_O–H_ = ∼3450 cm^–1^ corresponding to Mg^2+^ ligated H_2_O ligands (vide infra, Figures S3 and S4). The ^1^H NMR crown ether resonances of **1** appeared as broad, overlapping peaks between δ = 2.25–0.23 ppm, while the methylene resonance of the linker was clearly distinguishable as a doublet of doublets at δ = 3.39 ppm. This upfield shift in the crown ether ^1^H resonances of **1** relative to **L1** suggested that these protons were influenced by the ring current of the aromatic porphyrin core. The resolution of the geminal coupling in the methylene protons and the coupling to the neighboring methine proton also suggested a rigidification of the linker in **1** relative to **L1**. Together, these observations indicated that the pendant crown ether in **1** interacted closely with the porphyrin core in solution after Mg^2+^ insertion, a feature that was also observed in the solid-state structure of **1** (vide infra). The ^1^H NMR crown ether resonances of **2** were found as overlapping multiplets at δ = 2.80–1.64 ppm, and the two methylene resonances of the linkers were observed as one multiplet at δ = 3.83 ppm. These features were similar to the corresponding resonances in the ^1^H NMR spectrum of **L2** but with a slight downfield shift.

ESI-MS of **1** showed peaks corresponding to the [**1** + H]^+^, [**1** + NH_4_]^+^ and [**1** + Na]^+^ cations (calculated *m*/*z* = 927.3972, 944.4233 and 949.3791, found *m*/*z* = 927.3948, 944.4238 and 949.3757, respectively, Figure S10), while the ESI-MS spectrum of **2** showed peaks that were assigned to the [**2** + Na]^+^ and [**2** + 2Na]^2+^ cations (calculated *m*/*z* = 1197.5046 and 610.2469, found *m*/*z* = 1197.5033 and 610.2445, respectively, Figure S11). The observation of these ions by ESI-MS indicated that **1** and **2** were capable of cation sequestration vide infra. The electronic absorption spectra of **1**, **2**, and [Mg­(H_2_O)­(TTP)] were identical (Figures S12 and S13), suggesting that the crown ether substituents had a minimal effect on the electronic structure of the Mg-porphyrin core. The absorption bands were typical of Mg-porphyrin complexes: an intense Soret band at λ = 424 nm and two Q-bands of lesser intensity at λ = 564 and 604 nm.^57–59,62^ The reduction in the number of Q-bands from four in the freebases **L1** and **L2** to two in complexes **1** and **2** also served as evidence for complete metalation.^59^ Characterization of **1** and **2** by NMR, ESI-MS, FT-IR, and electronic absorption spectroscopy thus confirmed their identities as Mg-porphyrin complexes bearing one and two covalently linked crown ether substituents, respectively.

Crystals of **1** suitable for single crystal X-ray diffraction (SC-XRD) measurements were grown by vapor diffusion of pentane into a dilute solution of the complex in tetrahydrofuran (THF) at 20 °C. The structure obtained for **1** featured the Mg^2+^ ion in an octahedral coordination environment, coordinated by the tetradentate porphyrin ligand in the equatorial plane (Mg–N = 2.076–2.084 Å, Table S1) and by two H_2_O molecules in the axial positions (Figure 1). The Mg–N bond lengths were consistent with previously reported six-coordinate Mg-porphyrin complexes.^63–66^ The Mg–O distances for the two axially coordinated H_2_O molecules were found to be equal within error (2.106(5) and 2.103(4) Å), which is ∼0.1 Å shorter than analogous previously reported bis-H_2_O–Mg-porphyrin complexes.^64,65^ This observation was attributed to the H-bonding interactions between the crown ether O atoms and the protons of the axial H_2_O ligands (vide infra). Importantly, the crown was not ligated to any ions in the as-prepared complex, priming **1** for interaction with added cations. Instead, the crown ether was found to engage in intramolecular H-bonding with one of the axial H_2_O ligands. This interaction positioned the crown ether directly above and parallel to the porphyrin core, with distances of ∼3.8–4.8 Å between the O atoms of the crown and the porphyrin ring. Intermolecular hydrogen bonding between the other axial H_2_O molecule and the crown ether of a neighboring complex was observed upon examination of the crystal packing, giving rise to a stacked arrangement of units of **1** in the solid-state (Figure S14). Similar intra- and intermolecular H-bonding interactions have been observed in the solid-state structures of aza-crown appended Zn porphyrins.^67–69^ The solid-state structure of **1** therefore validated its spectroscopic characterization, critically showing that the appended crown ether was vacant and positioned in close proximity to the porphyrin ring.

**Figure 1:**
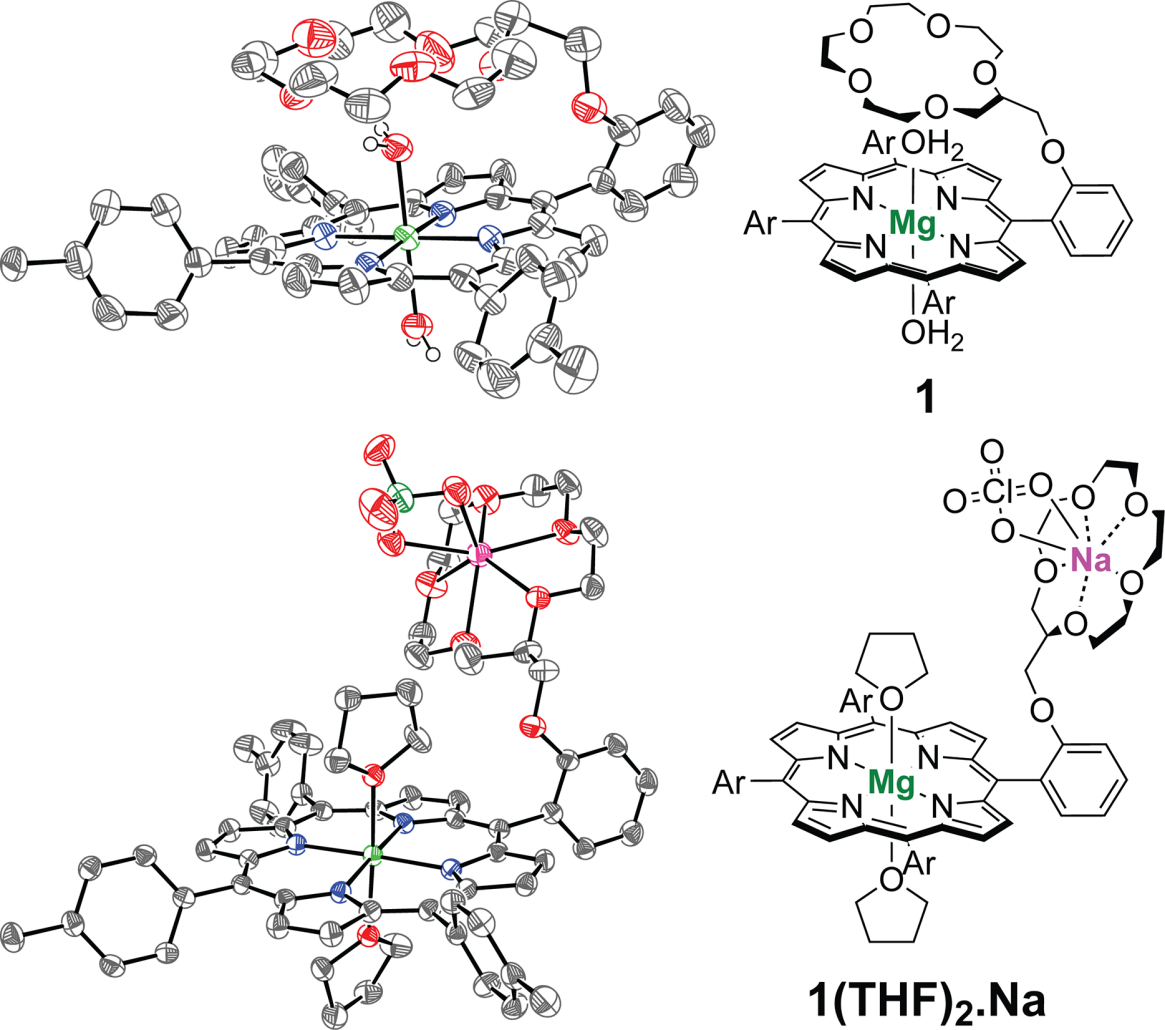
ORTEPs of **1** and **1­(THF)**_**2**_ **.Na** drawn at 50% probability. Solvent of crystallization and hydrogen atoms (excluding those of the axial H_2_O ligands of **1**) have been omitted for clarity. Crystallographic tables can be found in the Supporting Information (Tables S1 and S2). THF = tetrahydrofuran.

### Cation Binding Studies

We sought to investigate the ability of the crown ether substituents of **1** and **2** to bind redox-inactive cations. Diffusion of pentane into a NaClO_4_-saturated solution of **1** in THF yielded crystals of the NaClO_4_-bound complex as its THF adduct (**1­(THF)**_**2**_ **.Na**) that were suitable for SC-XRD measurements. The structure obtained displayed a 1:1 **1**/Na^+^ stoichiometry (Figure 1). The Na^+^ ion was found to be coordinated by the five O atoms of the 15-crown-5 moiety (Na–O­(crown) = 2.389–2.463 Å, Table S1) along with two O atoms of the ClO_4_^–^ anion (Na–O­(ClO_4_^–^) = 2.473, 2.501 Å), with the Na^+^ ion lying ∼0.8 Å from the least-squares plane defined by the crown O-atoms. These structural features are consistent with those reported for the NaClO_4_ complex of 15-crown-5.^70^ As in **1**, the Mg^2+^ ion was found in an octahedral coordination environment, ligated equatorially by the porphyrin ring (Mg–N = 2.067–2.077 Å, Table S1) with two THF O-donors occupying the axial positions, analogous to the H_2_O ligands observed in **1**. The Mg–O distances were inequivalent at 2.185 and 2.290 Å, where the longer distance corresponded to the axial THF ligand on the face of the porphyrin opposite the crown ether substituent. Examination of the crystal packing showed that the O atom of this THF molecule was engaged in intermolecular hydrogen bonding with a tolyl substituent of a neighboring complex (H36–O8 = 2.7 Å), giving rise to the elongated Mg–O distance (Figure S15).

With respect to the structure of **1**, the crown ether in **1­(THF)**_**2**_ **.Na** was tilted away from the porphyrin core, likely due to the loss of the intramolecular H-bonding interactions between the axial H_2_O ligand and crown ether that pulled the crown close to the porphyrin ring in **1**. This conformational change in the crown ether substituent resulted in significant separation between the Na and Mg sites of **1­(THF)**_**2**_ **.Na**; a minimum Na···porphyrin distance of ∼6.6 Å was observed, which is considerably longer than the porphyrin-crown ether distances observed in **1** (∼3.8–4.8 Å). Attempts to crystallize a NaClO_4_ adduct of **1** from noncoordinating solvents, such as CH_2_Cl_2_ or CHCl_3_, to eliminate THF binding, were unsuccessful.

As noted above, the metal-porphyrin interactions in **1­(THF)**_**2**_ **.Na** were consistent with reported solid-state structures of Mg-porphyrins and the metal-crown interactions were consistent with the solid-state structure of the NaClO_4_ complex of 15-crown-5. Furthermore, these coordination modes are distinct from those of Na-porphyrin complexes,^71^ and Mg^2+^ complexes of 15-crown-5 (and its derivatives) in the solid-state,^72–75^ indicating that the porphyrin-bound Mg^2+^ ion in **1** was inert to substitution by excess NaClO_4_. Indeed, Na-porphyrin complexes are known to be considerably less stable than Mg-porphyrin complexes, being readily demetalated in the presence of H_2_O.^71,76^ Overall, the solid-state structure of **1­(THF)**_**2**_ **.Na** confirmed the ability of the crown ether substituent in **1** to complex Na^+^ in the proximity of the porphyrin core in a 1:1 stoichiometry.

The interaction between NaClO_4_ and **1** was then investigated by ^1^H NMR spectroscopy. Titration of substoichiometric quantities of NaClO_4_ into a solution of **1** (5 mM, 9.5% CDCl_3_ in CD_3_CN, see Supporting Information for details) at 20 °C resulted in significant broadening of the peaks in its ^1^H NMR spectrum along with shifts to the crown ether resonances and the resonances of the phenyl group bearing the crown ether substituent (Figure S16). These observations indicated that an equilibrium existed between the NaClO_4_-bound complex (**1.Na**, as distinct from **1­(THF)**_**2**_ **.Na** due to differing solvation in CH_3_CN) and free **1** in CH_3_CN solution, and that Na^+^ interacted with the crown ether substituent of **1** in fast exchange on the NMR time scale. No shift was observed in the peaks corresponding to the *p*-tolyl substituents (δ = 8.13–8.00, 7.61–7.56, and 2.69 ppm) or pyrrolic protons (δ = 8.80–7.67 ppm), indicating that NaClO_4_ binding did not induce a significant change in the conformation of the porphyrin core. Nonlinear fitting of the chemical shifts of the phenyl protons (δ = 8.50, 7.74, 7.05 ppm) and the methylene group of the linker (δ = 3.42 ppm) as a function of [NaClO_4_] to a 1:1 binding model returned an association constant of log­(*K*_a_) = 3.9 ± 0.2, where the units of *K*_a_ are M^–1^ (Figure S16). This value was close to that reported for NaClO_4_ binding to 15-crown-5 in acetone measured by conductometry (log­(*K*_a_) = 4.26 ± 0.06)^77^ and is consistent with a strong affinity of the crown ether substituent of **1** for Na^+^ binding in CH_3_CN solution.

We then investigated the interaction between **1** and Mg­(ClO_4_)_2_ by ^1^H NMR spectroscopy. Titration of **1** against Mg­(ClO_4_)_2_ under identical conditions also significantly impacted the crown ether and aromatic resonances in the ^1^H NMR spectrum, though the effect was different to that observed for NaClO_4_. Instead of a gradual shift in the resonances observed with increasing salt concentration, substoichiometric addition of Mg­(ClO_4_)_2_ led to the appearance of new aromatic and aliphatic resonances along with the disappearance of the original resonances corresponding to **1** (Figure S17). For example, the methylene resonance of the linker decreased in intensity, with a small upfield shift, until it disappeared with the addition of one equivalent of Mg­(ClO_4_)_2_. Simultaneously, new peaks appeared in the range δ = 4.32–3.68 ppm, tentatively assigned to crown ether resonances of the Mg­(ClO_4_)_2_ adduct (**1.Mg**). Furthermore, the peaks observed for **1.Mg** remain relatively sharp by comparison to those observed in the NaClO_4_ titration, suggesting a lower degree of fluxionality in the Mg^2+^ adduct. These observations indicate that **1** interacted with Mg­(ClO_4_)_2_ via the crown ether substituent in a 1:1 stoichiometry with a binding constant beyond the limit of quantitation by NMR (log­(*K*_a_) ≥ 5), and that exchange between free **1** and **1.Mg** was slow on the NMR time scale.^78,79^ The lower limit obtained for log­(*K*_a_) in this case was close to the value measured for Mg­(ClO_4_)_2_ binding to 15-crown-5 by conductometry (log­(*K*_a_) = 4.74 ± 0.06).^80^ Together, the ^1^H NMR titrations against NaClO_4_ and Mg­(ClO_4_)_2_ showed a strong affinity of the crown ether substituent of **1** for complexing these salts in CH_3_CN solution via the pendant crown ether substituent in a 1:1 stoichiometry.

The electronic absorption spectrum of **1** was only slightly perturbed by the addition of NaClO_4_ or Mg­(ClO_4_)_2_ (Figures S18 and S19). These results indicate that cation binding had a negligible impact on the energies of its electronic transitions. The electronic absorption spectrum of **2** was also essentially unperturbed by the addition of NaClO_4_ or Mg­(ClO_4_)_2_ (Figures S20 and S21). Overall, the cation binding studies indicate that the crown ether substituent installed in the secondary coordination spheres of **1** and **2** exhibited affinities for Na^+^ and Mg^2+^ complexation that were broadly consistent with previously reported *K*_a_ values for 15-crown-5. The binding stoichiometry was determined to be 1:1 (cation/crown), meaning that one cation may bind to **1** and two cations may bind to **2**. Finally, cation binding did not impact the energies of the π–π* transitions of the complexes.

### Cyclic Voltammetry

The impact of cation binding on the electrochemical properties of **1** and **2** was then investigated. The cyclic voltammograms of **1** and **2** in the absence of added cations showed redox events typical of closed-shell metalloporphyrin complexes.^81^ In both cases two 1-electron redox couples were observed (*E*_1/2_ = 0.240, 0.580 V vs Fc/Fc^+^ for **1**, *E*_1/2_ = 0.230, 0.560 V vs Fc/Fc^+^ for **2**, Fc/Fc^+^ = ferrocene/ferrocenium), which were assigned to the formation of the porphyrin-π-cation radical and porphyrin-dication complexes, respectively (Figure S22).^81^ The scan rate dependence of the cathodic and anodic waves of the first redox couple of both complexes indicated that the 1-electron oxidized and neutral forms of **1** and **2** were freely diffusing in solution (Figures S23 and S24). Further, the peak potentials remained constant across multiple scan rates. These facts, along with the peak-to-peak separations (Δ*E*_p_ = 74 and 73 mV for **1** and **2**, respectively, at 200 mV/s, cf. Δ*E*_p_ = 96 mV for the Fc/Fc^+^ couple at 200 mV/s under identical conditions, Figure S25), indicated that formation of the porphyrin-π-cation radical complexes was *quasi*-reversible under the conditions of the cyclic voltammetry experiments.

The NaClO_4_ and Mg­(ClO_4_)_2_ adducts of **1** and **2** (**1.Na**, **2.Na**, **1.Mg**, **2.Mg**) were then generated in situ in CH_3_CN for electrochemical analysis. Addition of concentrated solutions of NaClO_4_ or Mg­(ClO_4_)_2_ into solutions of **1** and **2** in CH_3_CN brought about anodic shifts in *E*_1/2_ of the first redox event (oxidation to the π-cation radical, Figures 2 and S26, see Supporting Information for details). For both **1** and **2**, four equivalents of NaClO_4_ per crown ether and one equivalent of Mg­(ClO_4_)_2_ per crown ether were required to induce the maximum anodic shift. The requirement for excess NaClO_4_ to induce the maximum anodic shift was consistent with the ^1^H NMR titration data, which indicated that **1.Na** existed in equilibrium with free **1**/NaClO_4_ in CH_3_CN solution and that the maximum yield of **1.Na** was reached after the addition of four equivalents of NaClO_4_. Further, the fact that a stoichiometric quantity of Mg­(ClO_4_)_2_ was sufficient to reach the maximum anodic shift was also consistent with the ^1^H NMR titration data, which indicated that Mg­(ClO_4_)_2_ binding to **1** was stronger than that of NaClO_4_ and that a maximum yield of **1.Mg** was reached after the addition of one equivalent of Mg­(ClO_4_)_2_. Hence, we concluded that the potentials measured in the presence of the salts corresponded to the cation-bound complexes (Table 1), which were the dominant species in solution under the conditions of the cyclic voltammetry experiments. Variable scan rate experiments showed that the oxidized and reduced complexes remained freely diffusing upon cation binding and that the redox couples remained *quasi*-reversible (Figures S27–S30). Unfortunately, addition of salts of trivalent cations (e.g., Al­(ClO_4_)_3_, Sc­(OTf)_3_) led to decomposition of the Mg-porphyrin complexes, as evidenced by the appearance of a new feature in the electronic absorption spectrum at λ = 656 nm (Figure S31). This feature closely resembles the most prominent Q-band of protonated *meso*-tetraphenylporphyrin, indicating that these salts are unsuitable for this study due to their acidity in solution.^82^

**Figure 2:**
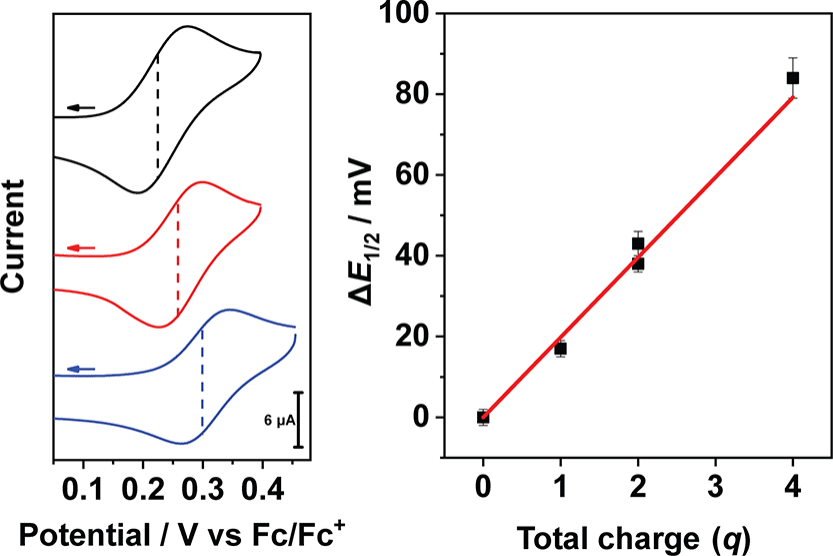
Left: Cyclic voltammograms showing the first redox event of **2** (black), showing the shift in *E*_1/2_ upon addition of NaClO_4_ (red, 4 equiv) and Mg­(ClO_4_)_2_ (blue, 1 equiv). Scan rate = 200 mV/s, electrolyte = 0.1 M NBu_4_PF_6_ in CH_3_CN, 20 °C. The arrows indicate the direction of the potential sweep (cathodic). Right: Maximum shifts to *E*_1/2_ of the first oxidation event of **1** and **2** as a function of total bound charge (*q*, the number of bound cations times the cation charge). *q* has been normalized by the elementary charge, *e* = 1.6 × 10^–19^ C. *R* ^2^ = 0.9864. Exact values and associated errors are given in Table S2.

**Table 1:** First Oxidation Potential of the Compounds of Relevance to the Present Study

	*E*_1/2_ (V vs Fc/Fc^+^)
[Mg(H_2_O)(TTP)]	0.23
**1**	0.24
**1.Na**	0.26
**1.Mg**	0.28
**2**	0.23
**2.Na**	0.27
**2.Mg**	0.31
Chl-*a*	0.16^a^
P700	–0.12^a^
P680	0.48–0.68^a^

a Converted from the value referenced against the standard hydrogen electrode (SHE) according to the previously reported Fc/Fc^+^ = +0.624 V vs SHE.^83^ Chl-*a* = chlorophyll-*a*. **1.M**, **2.M** (M = Na, Mg) refer to the Na and Mg adducts of **1** and **2**.

In control experiments, the first oxidation potential of [Mg­(H_2_O)­(TTP)] was unaffected by the addition of NaClO_4_ or Mg­(ClO_4_)_2_ (Figure S26). The observed shifts in *E*_1/2_ (Δ*E*_1/2_) for **1.Na**, **2.Na**, **1.Mg**, and **2.Mg** thus appear to be associated with the cations interacting with the appended crown ethers that are held in close proximity to the Mg-porphyrin core.

Taking the data for both **1** and **2** together, Δ*E*_1/2_ was found to vary linearly with the total charge exposed to the porphyrin with a slope of ∼20 mV per unit charge (Figure 2). Accordingly, the largest shift (+80 mV) was achieved upon addition of two equivalents of Mg­(ClO_4_)_2_ to **2** (a total charge of +4). Furthermore, a linear dependence of Δ*E*_1/2_ on charge was consistent with eq 1, which describes the electrostatic field potential (Δ*E*_1/2_) exerted by a point charge *q* at a distance *r* through a medium with dielectric constant ε.^34^ Hence, the observed linear dependence of Δ*E*_1/2_ on *q* is consistent with the origin of the shift in redox potential being an imposed electrostatic potential.

1 $$\Delta E_{1/2}=\frac{q}{4\pi \varepsilon r}$$

The magnitudes of Δ*E*_1/2_ were consistent with those observed by Zhong and co-workers for an aza-crown ether appended Co-porphyrin complex, where the redox-active entity was a Co^II^ ion. The bound cations appear to be held at similar distances from the redox-active entity in both of these systems (cation-porphyrin distance ∼6.6 Å for **1.(THF)**_**2**_ **Na**, 5.6–6.8 Å calculated by DFT for the Co^II^ system). Larger shifts in redox potential (∼100–700 mV) have been reported for cation binding to transition metal complexes of salen and salophen ligands, which differ from the present study in both the nature of the redox-active entity (a transition metal) and the proximity of the bound cation to the redox-active entity (∼3.4–3.7 Å, directly interacting with the primary coordination sphere of the transition metal).^34,38–40,43,45^ Based on the linear dependence of Δ*E*_1/2_ on *q*, and the invariance of the electronic absorption spectra of **1** and **2** upon cation binding, we concluded that the observed Δ*E*_1/2_ was caused by a through-space interaction with the bound cation(s), with negligible contributions from “through-bond” interactions.

The fact that the redox potentials of **1** and **2** increased upon cation binding, while their π–π* transitions were unaffected, is an interesting observation. We propose that the frontier molecular orbitals (MOs) of **1** and **2** were stabilized to similar extents upon cation binding. This effect would preserve the HOMO/LUMO gap energy, resulting in equivalent electronic absorption spectra between the free and cation-bound complexes, while increasing the redox potentials in the cation-bound complexes due to stabilization of the HOMO. This interpretation is consistent with previous semiempirical calculations on chlorophyll-*a*, which predicted a uniform stabilization of the frontier MOs in response to a static charge.^12^ To probe this idea further, we performed preliminary density functional theory (DFT) calculations at the B97-3c level of theory (see Supporting Information for details and data). The optimized structure of **1** was consistent with the solid-state structure measured by SC-XRD; for example, the Mg–N bond lengths were reproduced to within 0.01 Å (Figure S32). We then performed a rudimentary calculation of the energies of the frontier MOs of a number of isomers of **1** and **2** and their NaClO_4_ and Mg­(ClO_4_)_2_ adducts (Table S4). The HOMO–LUMO gap was found to vary by ∼0.01 eV across the series, suggesting that a near-uniform stabilization of the frontier MOs upon cation binding may indeed reconcile the electronic absorption spectra and the cyclic voltammetry data.

### Generation and Characterization of Mg-Porphyrin π-Cation Radical Complexes

We then sought to explore the impact of peripheral cationic charge on Mg-porphyrin π-cation radical complexes as synthetic mimics for P680^+^. Chemical oxidation of **1** and **2** was therefore investigated. Addition of one equivalent of the 1-electron oxidants [Ru­(bpy)_3_]­(PF_6_)_3_ (bpy = 2,2′-bipyridine), ceric ammonium nitrate (CAN), or tris­(*p*-tolyl)­ammoniumyl hexachloroantimonate ([N­(*p*-tol)_3_]­SbCl_6_) to solutions of **1** and **2** in CH_3_CN at 20 °C resulted in the formation of new species (**1** ^**•+**^ and **2** ^**•+**^) as judged by electronic absorption spectroscopy (Figures 3, S33–S36). The absorption spectra of **1** ^**•+**^ and **2** ^**•+**^ were almost identical, characterized by a weakened and blue-shifted Soret band (λ = 409 nm), sharp bands at λ = 362 and 364 nm, respectively, and broad overlapping features toward the red end of the visible spectrum (λ = 515–840 nm). These features were highly consistent with previously characterized Mg-porphyrin π-cation radicals with an A_2u_-type ground state; previously reported Mg-porphyrin π-cation radicals with A_1u_ ground states instead display split Soret bands and a pronounced Q-band between λ = 680–700 nm.^84,85^ Substoichiometric additions of oxidant gave maximum yields of **1** ^**•+**^ and **2** ^**•+**^ after the addition of precisely one equivalent of oxidant.

**Figure 3:**
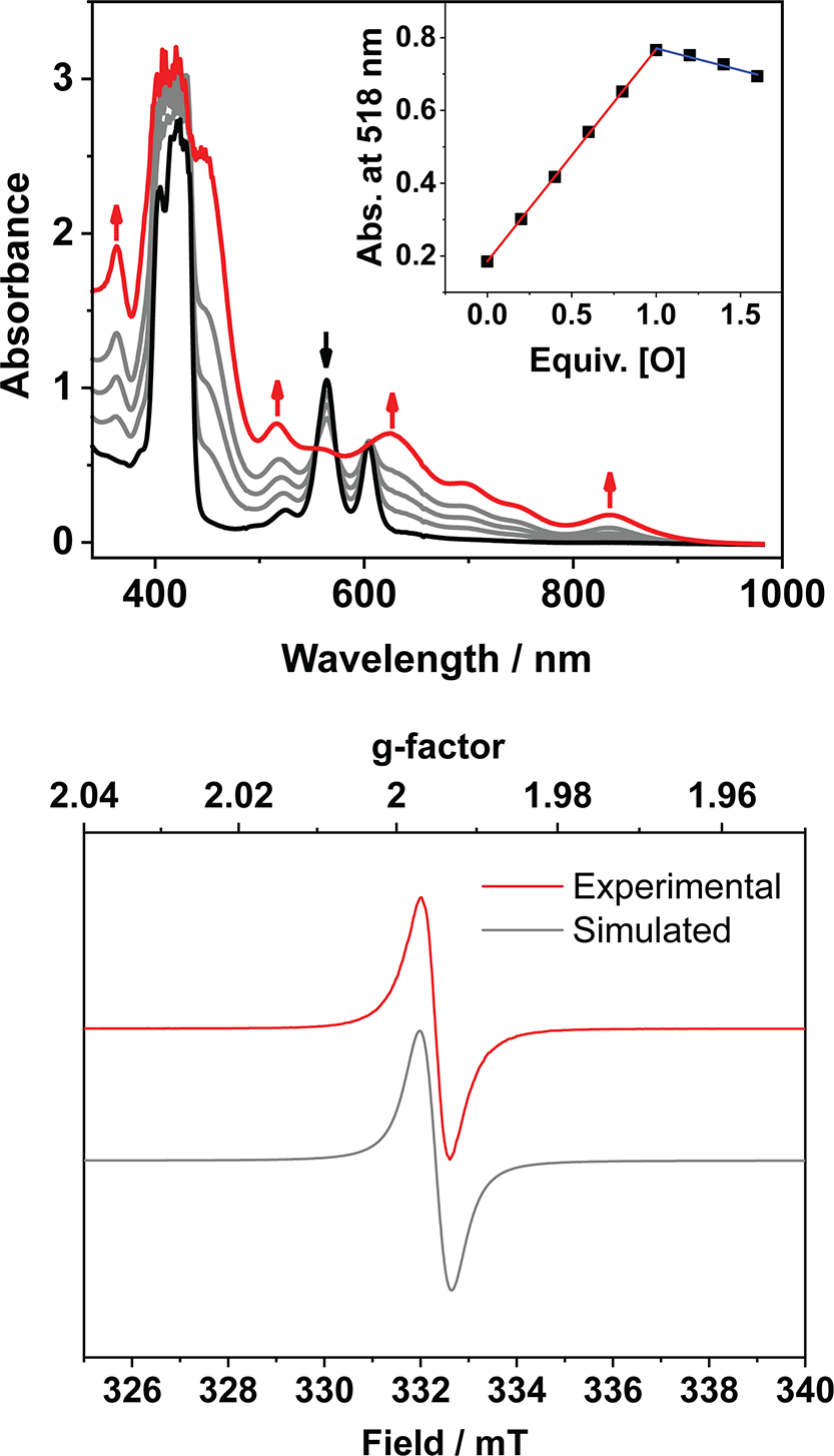
Top: Electronic absorption spectra of **2** (black trace, CH_3_CN, 60 μM) and new species **2** ^**•+**^ (red trace) formed upon addition of [Ru­(bpy)_3_]­(PF_6_)_3_ (1 equiv) at 20 °C. Inset: Titration of **2** with substoichiometric quantities of oxidant ([O]), showing maximum yield of **2** ^**•+**^ at one equivalent. Bottom: X-band electron paramagnetic resonance (EPR) spectrum of **2** ^**•+**^ in frozen CH_3_CN solution (77 K, red trace) and the simulated spectrum (gray trace, see Supporting Information for simulation details). Instrumental parameters: 0.2 mW power, 9.2 GHz frequency and 0.3 mT modulation amplitude.

The X-band electron paramagnetic resonance (EPR) spectrum of **2** ^**•+**^ showed a single isotropic signal centered at *g* = 1.99, typical of Mg-porphyrin π-cation radical complexes and organic radical species generally (Figure 3).^84,85^ Double integration of the spectrum returned a yield of 100 ± 20% with reference to an external TEMPO (2,2,6,6-tetramethyl-piperidin-1-yl)­oxyl radical standard, indicating a quantitative yield of **2** ^**•+**^.

The FT-IR spectra of **1** ^**•+**^ and **2** ^**•+**^ showed new peaks at ν = 1271 and 1270 cm^–1^, respectively, that were not present in the spectra of the neutral species (Figures S37 and S38). New vibrational bands in the ν = 1250–1280 cm^–1^ range are characteristic of porphyrin π-cation radicals with an A_2u_ ground state, which further supports our assignment of **1** ^**•+**^ and **2** ^**•+**^ as such.^86^ Positive mode ESI-MS of **1** ^**•+**^ showed a peak corresponding to the [**1**]^**+**^ cation (calculated *m*/*z* = 926.3888, found *m*/*z* = 926.3906, Figure S39), while ESI-MS of **2** ^**•+**^ showed peaks corresponding to the [**2**]^**+**^ and [**2** + Na]^2+^ cations (calculated *m*/*z* = 1174.5154 and 598.7520, found *m*/*z* = 1174.5156 and 598.7515 respectively, Figure S40). These ions were absent from the ESI-MS spectra of **1** and **2,** and their valencies support the assignment of **1** ^**•+**^ and **2** ^**•+**^ as radical cation species. Based on the combined characterization data we concluded that **1** ^**•+**^ and **2** ^**•+**^ were Mg-porphyrin π-cation radical species that formed in quantitative yield upon 1-electron oxidation of **1** and **2** with chemical oxidants.

### Reactivity Studies

The half-lives of **1** ^**•+**^ and **2** ^**•+**^ were found to be greater than 2 h at 20 °C in CH_3_CN, allowing sufficient time for their PCET reactivity toward external substrates to be investigated. We sought to mimic the reaction between P680^+^ and its natural substrate (a tyrosine residue); therefore, 2,6-di-*tert*-butyl-4-methoxyphenol (4-CH_3_O-2,6-DTBP, Scheme 2) was chosen as a suitable phenolic substrate due to its well-documented PCET reactivity.^87^ The reactions were conducted under *pseudo* first-order conditions (≥10 equiv 4-CH_3_O-2,6-DTBP) and were followed by electronic absorption spectroscopy. Addition of the substrate to **1** ^**•+**^ or **2** ^**•+**^ in CH_3_CN at 20 °C resulted in a rapid decay of the absorption features associated with the π-cation radical species (Figures 4 and S41). The Q-bands of **1** and **2** were found to reform from these reactions in approximately 75–80% yield based on the final absorption intensity (Figures 4 and S41). This indicated that **1** ^**•+**^ and **2** ^**•+**^ acted as 1-electron oxidants, producing their 1-electron reduced precursors in good yield. Exponential fitting of the decay of the cation radical absorption feature at λ = 362 and 364 nm for **1** ^**•+**^ and **2** ^**•+**^, respectively, yielded the *pseudo*-first order rate constants (*k*_obs_) for the reactions. The two rates were found to be comparable (*k*_obs_(**1** ^**•+**^) = (1.74 ± 0.03) × 10^–2^, *k*_obs_(**2** ^**•+**^) = (1.1 ± 0.1) × 10^–2^ for 20 equiv substrate), indicating that the presence of a second crown ether substituent in **2** ^**•+**^ did not substantially impact the rate of PCET.

**Scheme 2:**
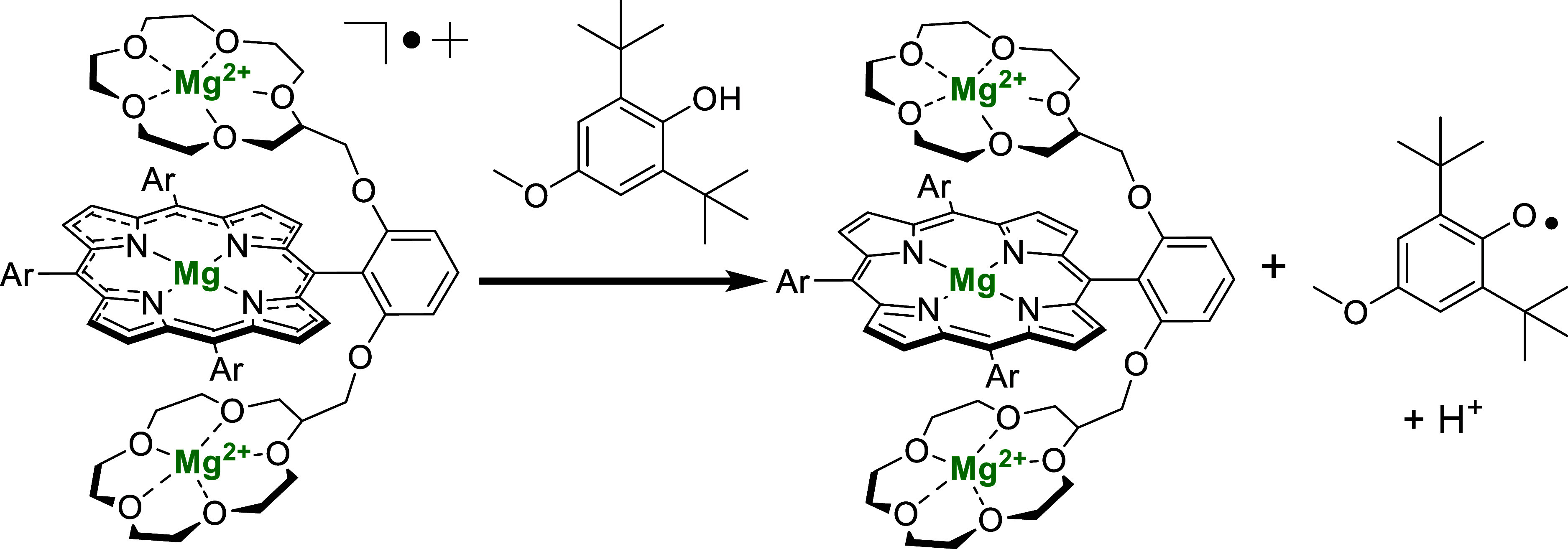
Representation of the Reaction between Mg^2+^-Bound **2** ^**•+**^ and 4-CH_3_O-2,6-DTBP^a^

a Ar = *p*-tolyl.

**Figure 4:**
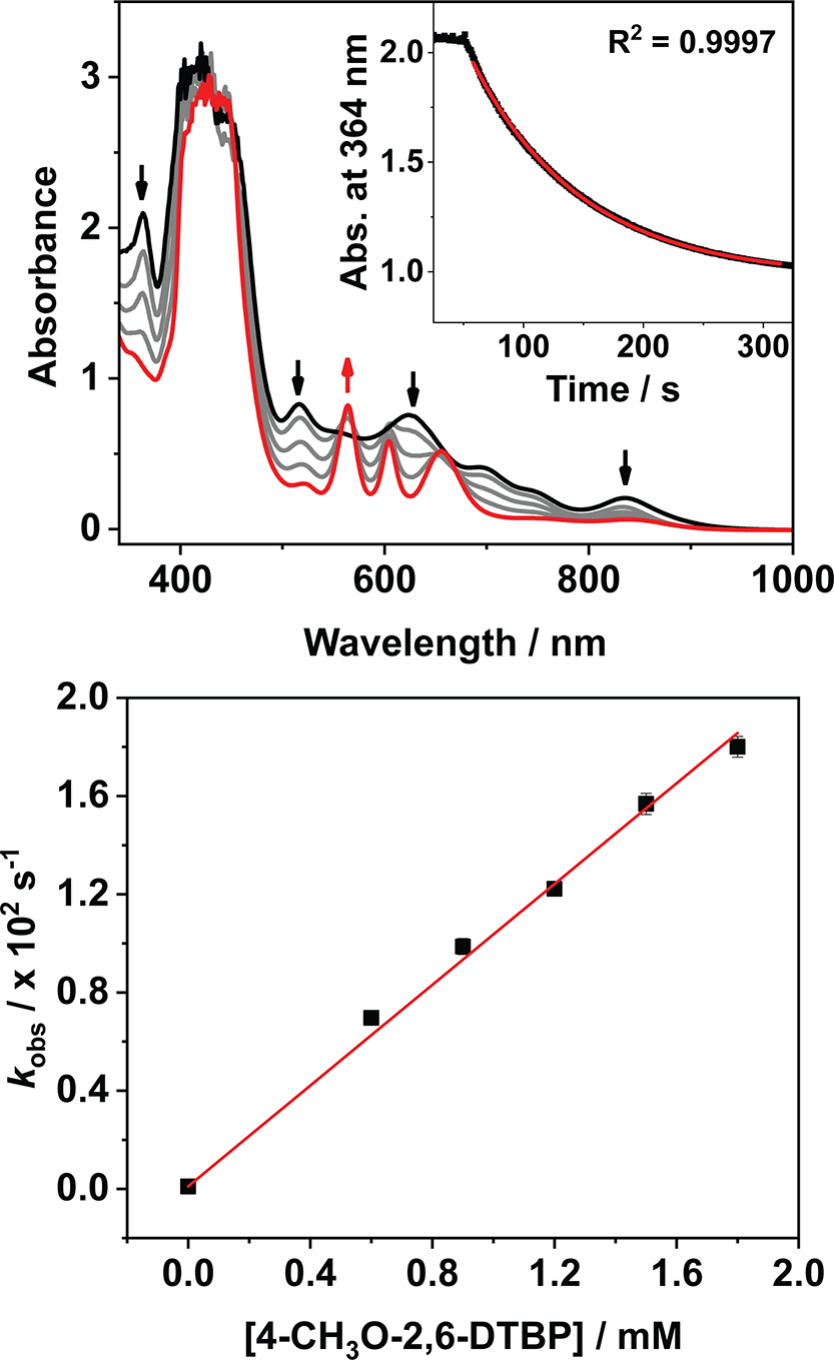
Top: Changes to the electronic absorption spectrum of **2** ^**•+**^ (black trace, 60 μM, CH_3_CN) upon reaction with 4-CH_3_O-2,6-DTBP (10 equiv, producing 1 (red trace)) at 20 °C. Inset: Exponential fit of the decay of the absorption features of **2** ^**•+**^ at λ = 364 nm. Bottom: Plot of *k*_obs_ against the concentration of substrate for the same reaction at varying [4-CH_3_O-2,6-DTBP].

New absorption features at λ = 656, 750, 840 nm were evident in the postreaction electronic absorption spectra (Figures S41 and S42). These new species presumably correspond to side-products of the reaction between **1** ^**•+**^ and **2** ^**•+**^ and the substrate, accounting for the “missing” 20–25% of **1** and **2** in the post reaction mixtures. The lower-energy features (λ = 750, 840 nm) are typical of *iso*-porphyrin species, and post reaction ESI-MS of the reaction between 4-CH_3_O-2,6-DTBP and **2** ^**•+**^ showed a peak at *m*/*z* = 1191.5186, consistent with a hydroxylated *iso*-porphyrin species (calculated *m*/*z* = 1191.5176 for [**2** + OH]^+^, Figure S43).^88^ Furthermore, the feature at λ = 656 nm closely resembles the most prominent Q-band of protonated *meso*-tetraphenylporphyrin,^82^ and a mass attributable to the proto-demetalated ligand ([**L2** + H]^+^, calculated *m*/*z* = 1153.5538, found *m*/*z* = 1153.5546) was also present in the same postreaction ESI-MS. As the p*K*_a_ of 1-electron oxidized phenols is very low, and Mg-porphyrins are very sensitive to acid, this species presumably formed due to the release of the phenolic protons upon oxidation of the substrate.^87^

The initial phenol-derived product resulting from this PCET reaction was anticipated to be the corresponding 2,6-di-*tert*-butyl-4-methoxyphenoxyl radical. The EPR spectra of the post reaction mixtures were silent, however, presumably owing to the rapid decomposition of the radical under the reaction conditions. Gas chromatographic analysis of the post reaction mixture of the reaction of **2** ^**•+**^ with 4-CH_3_O-2,6-DTBP showed 2,6-di-*tert*-butylbenzoquinone (DTBQ) present at an equimolar concentration with respect to the initial concentration of ****2** ^**•+**^** (100% yield, Figure S44). As DTBQ is a 2e^–^ oxidation product of 4-CH_3_O-2,6-DTBP (alongside the loss of one H^+^), a 50% yield of DTBQ with respect to the concentration of would be expected if the cation radical species were the sole oxidant responsible for conversion of 4-CH_3_O-2,6-DTBP to DTBQ. As the yield of DTBQ was quantitative with respect to the cation radical species, we assume that this product forms through aerobic oxidation of the phenoxyl radical formed *after* the initial 1-electron PCET oxidation of the substrate by **1** ^**•+**^/**2** ^**•+**^ (Scheme S2).

We then endeavored to probe the mechanism of these reactions. Measuring *k*_obs_ for the reaction between **2** ^**•+**^ and 4-CH_3_O-2,6-DTBP at varying substrate concentrations yielded a linear relationship which could be fit to obtain the second order rate constant, *k*_2_ = 10 M^–1^ s^–1^ (Figure 4). Kinetic isotope effect (KIE) experiments with deuterated substrate ^2^H-*O*-2,6-di-*tert*-butyl-4-methoxyphenol (4-CH_3_O-2,6-DTBP-OD) returned a value of *k*_H_/*k*_D_ ≈ 1, indicating that the reaction did not proceed via rate limiting proton transfer (PT), hydrogen atom transfer (HAT), or concerted proton and electron transfer (CPET) and that electron transfer (ET) was therefore likely rate-limiting (Figure S45).

To further probe the proton-dependence of the reaction we pursued a comparable substrate that lacked an OH group. In this context, **1** ^**•+**^ and **2** ^**•+**^ were found to react with the diether 1,3-di-*tert*-butyl-2,5-dimethoxybenzene (1,3-DTB-2,5-DMB), which was prepared by methylation of the phenolic OH of 4-CH_3_O-2,6-DTBP (see Supporting Information for details). During these reactions, the neutral species **1** and **2** were found to reform in approximately 75–80% yield based on the final absorption intensity, indicating that **1** ^**•+**^ and **2** ^**•+**^ acted as 1-electron oxidants once again (Figures S46 and S47). The only salient difference between the reactions of the cation radical species with the two substrates was that the reaction with 1,3-DTB-2,5-DMB was slower (*k*_2_ ≈ 0.4 M^–1^ s^–1^ versus 10 M^–1^ s^–1^ for **2** ^**•+**^), owing to the increased redox potential of 1,3-DTB-2,5-DMB relative to 4-CH_3_O-2,6-DTBP.^89,90^ Nonetheless, the fact that the radical cations were competent for the 1-electron oxidation of a comparable substrate lacking an OH group supported our assessment that **1** ^**•+**^ and **2** ^**•+**^ reacted with 4-CH_3_O-2,6-DTBP via rate-limiting ET.

We then sought to probe the impact of cation binding on the spectroscopic and reactivity properties of **1** ^**•+**^ and **2** ^**•+**^. We found that both species could be generated in comparable yields by oxidation of the parent Mg-porphyrin complexes in the presence of excess NaClO_4_ and Mg­(ClO_4_)_2_ (10 equiv, Figures S48 and S49). Little-to-no discrepancies in the electronic absorption spectra of **1** ^**•+**^ or **2** ^**•+**^ in the presence of the salts were identified. In light of the evidence for Mg^2+^ binding to **2** ^**•+**^ detailed below, this result indicated that cation binding to the π-cation radical complexes did not substantially impact the energies of their electronic transitions.

As **2** has the ability to bind the maximum peripheral charge, a direct comparison of the reactivity of **2** ^**•+**^ in the presence/absence of Mg­(ClO_4_)_2_ was first made. Reacting **2** ^**•+**^ with 4-CH_3_O-2,6-DTBP (10 equiv) in the presence of Mg­(ClO_4_)_2_ (10 equiv) gave a marked increase in *k*_obs_ relative to the analogous experiment in the absence of Mg­(ClO_4_)_2_ (*k*_obs_ = 0.414 s^–1^ vs 0.070 s^–1^, Figure S50). Measuring *k*_obs_ for the reaction between **2** ^**•+**^ and a fixed concentration of substrate while varying the Mg­(ClO_4_)_2_ concentration (1–10 equiv) revealed a nonlinear relationship (Figure 5). This behavior was indicative of an equilibrium corresponding to Mg­(ClO_4_)_2_ binding to **2** ^**•+**^ in solution, resulting in a concentration-dependent increase in *k*_obs_ that approaches a saturation value. The data was therefore fit to an offset Hill function: eq 2, where *n* is the Hill coefficient, [*S*] is the substrate concentration, *k*_2_^min^ is the value of *k*_2_ in the absence of Mg^2+^, *k*_2_^max^ is the value of *k*_2_ in the presence of a large excess of Mg^2+^ (i.e., [Mg]^*n*^/(*K* ^*n*^ + [Mg]^*n*^) ≈ 1) and *K* is a constant (Figure 5, see Supporting Information for details).

2 $$k_{obs}=\left({k_{2}}^{\min}+\left({k_{2}}^{\max}-{k_{2}}^{\min}\right)\cdot \frac{{\left[Mg\right]}^{n}}{K^{n}+{\left[Mg\right]}^{n}}\right)\left[S\right]$$

**Figure 5:**
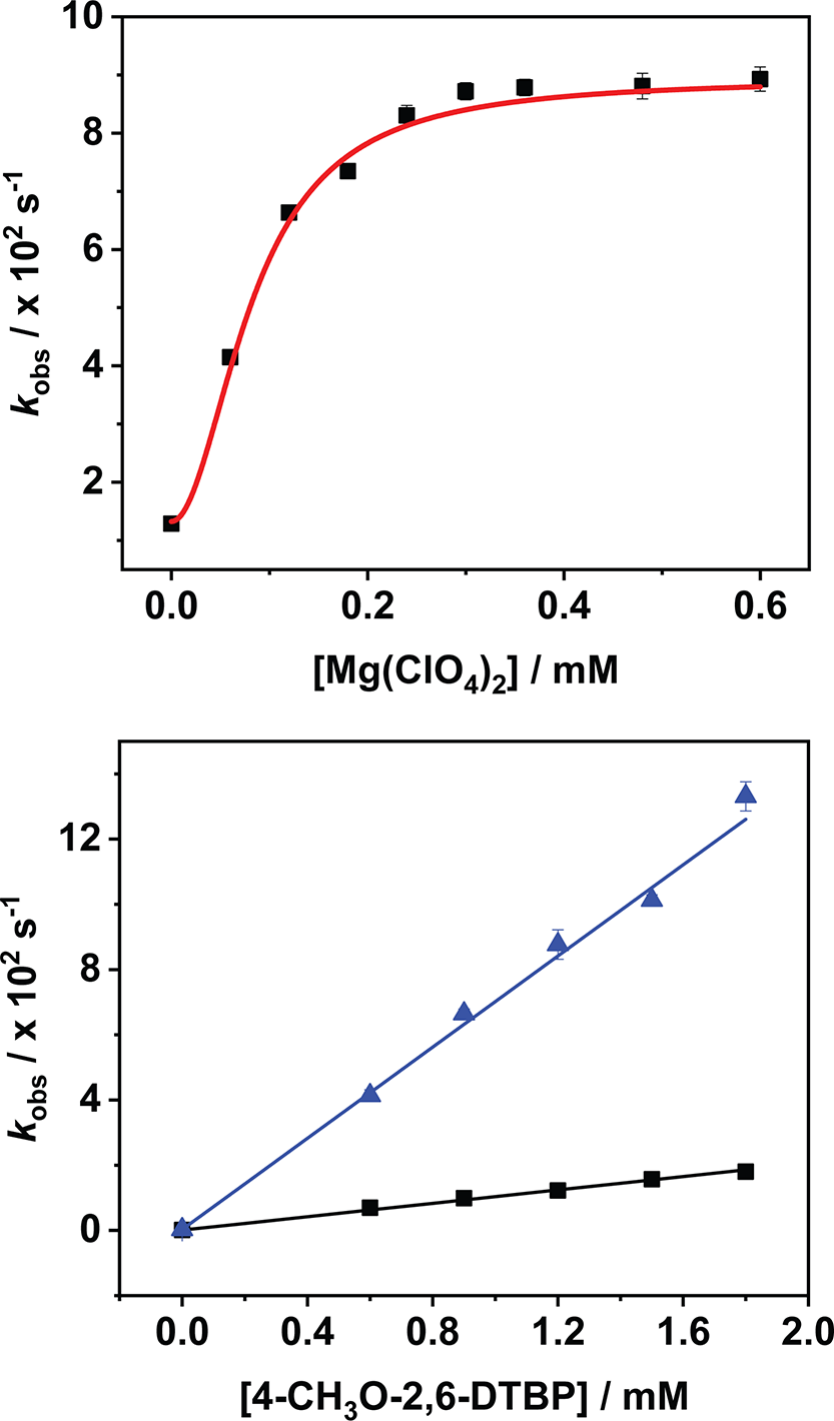
Top: Nonlinear dependence of *k*_obs_ on the concentration of Mg­(ClO_4_)_2_ for the reaction between and 4-CH_3_O-2,6-DTBP (20 equiv, see Supporting Information for fit details). *R* ^2^ = 0.9966. Bottom: Linear dependence of *k*_obs_ on substrate concentration, where *k*_2_ is the slope of the linear fits. Black = absence of Mg­(ClO_4_)_2_ (*R* ^2^ = 0.9981), blue = 10 equiv of Mg­(ClO_4_)_2_ (*R* ^2^ = 0.9977).

A value of *n* = 2 was returned by the nonlinear regression analysis, consistent with two Mg^2+^ ions binding to **2** ^**•+**^. Furthermore, *K* is a measure of the dissociation constant (*K*_d_), allowing for an estimate of the association constant (*K*_a_) of Mg^2+^ binding to **2** ^**•+**^ to be made. A value of log­(*K*_a_) = 4.0 ± 0.3 was obtained. This result is consistent with a previously reported value for the association of Mg^2+^ with benzo-15-crown-5 in CH_3_CN at 25 °C,^91^ but somewhat reduced when compared to the results of the ^1^H NMR titration of **1** with Mg^2+^ (log­(*K*_a_) ≥ 5). Finally, taking the ratio of the limiting value of *k*_obs_ and the initial value revealed a maximum rate enhancement of 6.9-fold. The magnitude of the rate enhancement was consistent with the estimated rate enhancement predicted by the simplified Marcus equation (Equation S3) for the 80 mV increase in redox potential that was measured for Mg^2+^ binding to **2** (predicted rate enhancement of ∼4.8-fold for an outer-sphere ET reaction assuming *E*_1/2_ = *E* ^0^).^92–94^

As with **2** ^**•+**^, the post reaction mixture for the reaction in the presence of Mg­(ClO_4_)_2_ showed the Q-bands of **2** (λ = 564, 604 nm) alongside small quantities of decay products (the protonated ligand, Figure S50). The yield of reformed **2** in the presence of Mg­(ClO_4_)_2_ was slightly higher (90%). In order to gain insight into the mechanism of the reaction in the presence of Mg­(ClO_4_)_2_, **2** ^**•+**^ was reacted with deuterated substrate (4-CH_3_O-2,6-DTBP-OD) in the presence of Mg­(ClO_4_)_2_ (10 equiv). A KIE value of *k*_H_/*k*_D_ = 1.3 was returned, indicating that PT, HAT, or CPET were unlikely to be rate limiting and that ET was likely rate-limiting in this PCET reaction (Figure S51). Hence, the binding of Mg^2+^ to **2** ^**•+**^ and the presence of excess Mg­(ClO_4_)_2_ appeared not to change the mechanism of the reaction.

Linear fitting of a plot of *k*_obs_ against [4-CH_3_O-2,6-DTBP] in the presence of a fixed excess of Mg­(ClO_4_)_2_ (10 equiv, in the saturated region of Figure 5) returned the limiting value of *k*_2_^max^ = 70 M^–1^ s^–1^ (Figures 5 and S50). This result represented a marked increase in the *k*_2_ value determined for the oxidation of the same substrate by **2** ^**•+**^ in the absence of cations (10 M^–1^ s^–1^). Furthermore, the measured value agreed well with the rate enhancement predicted from the nonlinear fit of *k*_obs_ against Mg­(ClO_4_)_2_ concentration (Figure 5). To further control for the role of the crown ether substituents in this reactivity enhancement, we reacted the π-cation radical complex [Mg­(TTP^**•**^)]^**+**^ with 4-CH_3_O-2,6-DTBP in the presence and absence of Mg­(ClO_4_)_2_. No change to the rate of substrate oxidation in the presence of Mg­(ClO_4_)_2_ was observed in these control experiments, ruling out a nonspecific interaction between the Mg-porphyrin core and the added salt (Figures S52 and S53). This result was consistent with our Hill analysis above, which indicated that a specific binding interaction between **2** ^**•+**^ and Mg^2+^ was occurring. Hence, the Mg^2+^ dependence of the rate of phenol oxidation by **2** ^**•+**^ was consistent with Mg^2+^ binding to **2** ^**•+**^ via its crown ether substituents, leading to an increase in the rate of electron transfer from a phenolic substrate that was consistent with our cyclic voltammetry results.

The impact of Mg^2+^ binding on the PCET reactivity of **1** ^**•+**^ was then probed. The reaction between **1** ^**•+**^ and 4-CH_3_O-2,6-DTBP (20 equiv) in the presence of Mg­(ClO_4_)_2_ (10 equiv) proceeded analogously to the reaction in the absence of Mg­(ClO_4_)_2_, producing a postreaction electronic absorption spectrum with features corresponding to **1** (90% yield) and protonated ligand (λ = 656 nm, Figure S54). Measuring *k*_obs_ for this reaction revealed a 2.5-fold increase in the rate relative to the reaction in the absence of Mg­(ClO_4_)_2_. This is a smaller rate enhancement compared to the analogous result for Mg^2+^ binding to **2** ^**•+**^ (∼7-fold enhancement). Hence, the rate of substrate oxidation appeared to trend positively with the peripheral charge bound to the porphyrin (*q*), as the total bound charge for Mg^2+^ binding to **1** ^**•+**^ was 2+ (1 equiv Mg^2+^) and to **2** ^**•+**^ was 4+ (2 equiv Mg^2+^).

We endeavored to measure the impact of Na^+^ binding to **1** ^**•+**^ and **2** ^**•+**^ on their rates of reaction with 4-CH_3_O-2,6-DTBP to further investigate the relationship between *k*_obs_ and *q*. Unfortunately, the Na^+^ adducts of **1** ^**•+**^ and **2** ^**•+**^ underwent excessive ligand proto-demetalation during the course of the reaction (2-fold increase in the yield of protonated ligand (λ = 656 nm) observed in the presence of NaClO_4_, Figure S55), which prevented a productive analysis. This problem was circumvented by utilizing the methylated substrate 1,3-DTB-2,5-DMB. **1** ^**•+**^ and **2** ^**•+**^ were reacted with 1,3-DTB-2,5-DMB under *pseudo*-first order conditions (120 equiv substrate) under three separate conditions: (i) in the absence of perchlorate salts, (ii) in the presence of NaClO_4_ (100 equiv), and (iii) in the presence of Mg­(ClO_4_)_2_ (10 equiv). A 10-fold excess of NaClO_4_ was used relative to Mg­(ClO_4_)_2_ to ensure full occupation of the crown ether sites (Figures 5, S16 and S17). The reactions were monitored by electronic absorption spectroscopy as before, with yields of 75–90% of **1** and **2** returned as judged by the final absorption intensities (Figures S47 and S56–S60). This indicated that ligand proto-demetalation was indeed suppressed and that **1** ^**•+**^ and **2** ^**•+**^ were facilitating 1-electron oxidation of the 1,3-DTB-2,5-DMB.

*k*_obs_ for these reactions were obtained as before (Figures S47 and S56–S60). Taking the data for **1** ^**•+**^ and **2** ^**•+**^ together, *k*_obs_ was found to trend positively with *q*. This result was interpreted using Marcus theory, which predicts that a plot of log­(*k*_ET_) against changes in redox potential (Δ*E*_1/2_) should be linear for an ET reaction with a slope of 0.5 (eq S3).^92–94^ Plotting log­(*k*_obs_) against Δ*E*_1/2_ for the reactions of **1** ^**•+**^, **2** ^**•+**^, and their Na^+^/Mg^2+^ adducts with 1,3-DTB-2,5-DMB revealed a linear correlation with a slope of 0.44, indicating that our results were consistent with Marcus theory (Figures 6 and S61). As we had previously demonstrated that Δ*E*_1/2_ was linearly dependent on *q* (Figure 2), it follows that log­(*k*_obs_) should also be linearly dependent on *q* (eq S4). A plot of log­(*k*_obs_) against *q* revealed a linear relationship with a slope of 9.2 ± 0.3 (Figure 6). Consistent with eqs 1 and S4, this slope is approximately 0.5 times the slope of the Δ*E*_1/2_ vs *q* relation described above (Figure 2, slope ∼20 mV per unit charge). We were therefore successful in unifying our cyclic voltammetry results and measured ET kinetics by means of Marcus theory and simple electrostatics, demonstrating a linear relationship between log­(*k*_obs_) and *q*. We therefore concluded that the measured rate enhancements in the PCET and ET reactions performed by **1** ^**•+**^ and **2** ^**•+**^ in this study were due to the cationic charges imposed on the Mg-porphyrins upon peripheral cation binding, which increased their redox potentials (and consequently ET rates) via the through-space action of the resulting electrostatic field.

**Figure 6:**
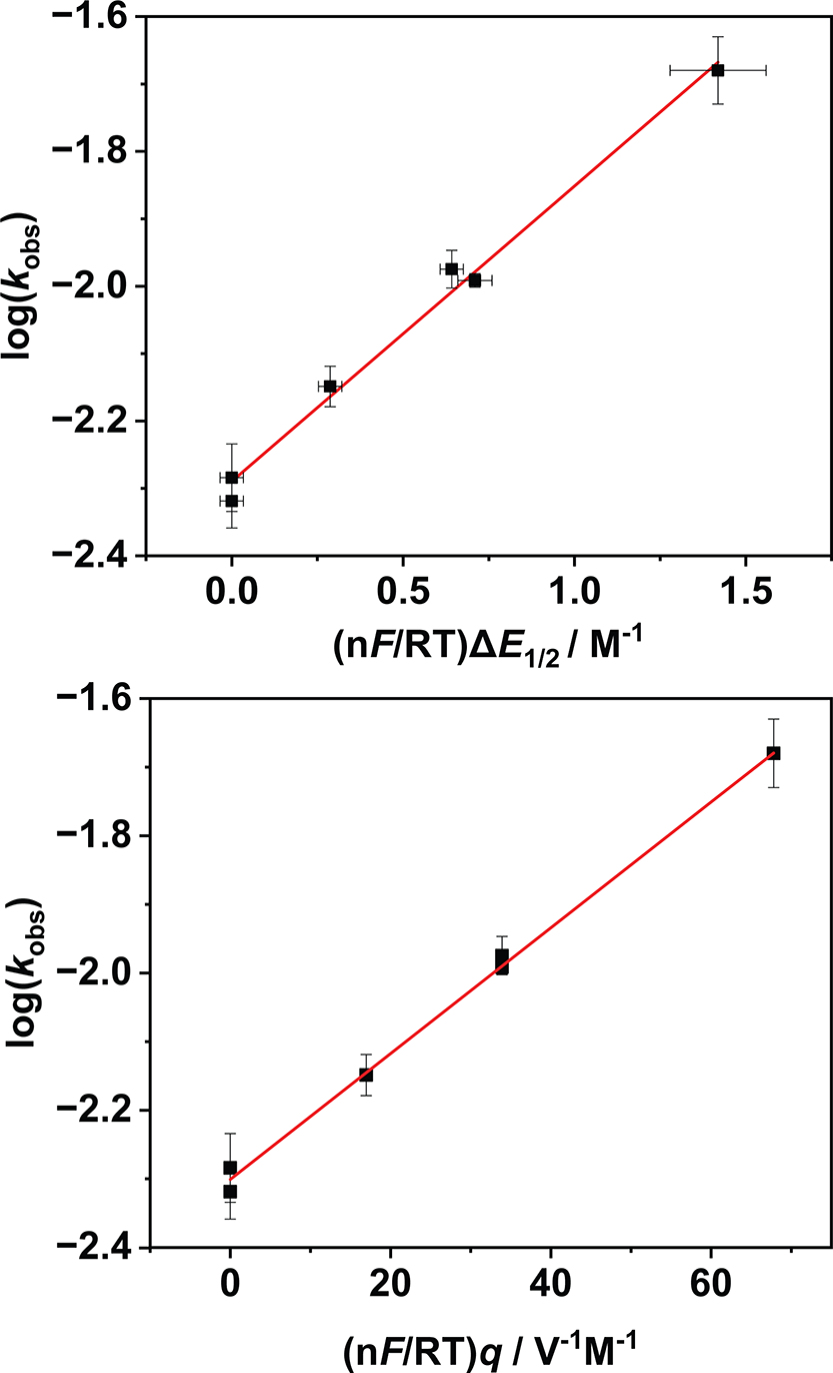
Top: Plot of log­(*k*_obs_) against Δ*E*_1/2_ for the reaction of **1** ^**•+**^ and **2** ^**•+**^ with 1,3-DTB-2,5-DMB (120 equiv, CH_3_CN, 20 °C). Bottom: Plot of log­(*k*_obs_) against *q* for the reaction of **1** ^**•+**^ and **2** ^**•+**^ with 1,3-DTB-2,5-DMB (120 equiv, CH_3_CN, 20 °C). *q* has been normalized by the elementary charge, *e* = 1.6 × 10^–19^ C.

Previous theoretical studies have implicated the electrostatic environment around the RC in PSII as a significant contributor to the increased redox potential of P680 relative to other photosynthetic pigments, attributing up to 450 mV of the 600–800 mV difference in redox potential between PSII and PSI to the atomic charges of the protein matrix.^17–19^ The present study explicitly demonstrates that the redox chemistry of Mg-porphyrin complexes can be tuned via through-space interactions with charged entities in their secondary coordination spheres for the first time. Our results therefore indicate that modulation of the electrostatic environment is a viable mechanism through which the redox properties of chlorophylls may also be tuned, providing direct experimental support to previous predictions. The magnitude of the effect is larger in PSII than that observed for the model systems presented here, likely owing to the fact that PSII contains a large number of charged residues that may contribute to the electrostatic potential it exerts on P680 (molecular weight of PSII ∼350 kDa).^95^ Replicating the magnitude of the influence of electrostatic fields on the reactivity of PSII in synthetic porphyrinoid systems therefore remains a challenge. Advances toward this goal promise to provide further insight on the reactivity properties of chlorophyll-based pigments (such as P680) and porphyrinoid cofactors generally. This work should also further the development of electrostatic field considerations as a means to design functional synthetic metal complexes supported by porphyrinoid ligands (e.g., for applications in oxidation catalysts).

## Conclusions

Cation binding to crown ether-appended Mg-porphyrin complexes **1** and **2** was demonstrated, resulting in anodic shifts to their 1-electron oxidation potentials of up to 80 mV. The magnitude of the anodic shifts was found to vary linearly with the total charge exposed to the complex, consistent with this effect arising from the electrostatic field potential exerted by the bound cation(s) and their net charge. These complexes were oxidized by 1-electron oxidants to yield Mg-porphyrin π-cation radical complexes (**1** ^**•+**^ and **2** ^**•+**^), which were characterized by electronic absorption, FT-IR, and EPR spectroscopies and ESI-MS. **1** ^**•+**^ and **2** ^**•+**^ were found to react as 1-electron oxidants, oxidizing a phenolic substrate by rate-limiting electron transfer, mimicking the reaction between photo-oxidized chlorophyll and tyrosine in PSII. Furthermore, Mg^2+^ binding to the crown ethers in the π-cation radical species was demonstrated and found to increase the rate of phenol oxidation in a charge-dependent manner up to a 7-fold acceleration. The relationship between the bound peripheral charge and the rate of 1-electron oxidation of a methylated substrate (no OH group) by **1** ^**•+**^ and **2** ^**•+**^ was demonstrated and formalized in terms of Marcus theory and simple electrostatics. Overall, we have experimentally demonstrated that proximal cationic charges can tune the redox properties of chlorophyll model compounds leading to an enhancement in their PCET reactivity. As an experimental verification of the viability of this previously predicted/calculated effect, this work has implications for our understanding of the factors that govern the redox chemistry of photosynthetic pigments.

## Supplementary Material



## Notes

Physical and synthetic methods; characterization of **L1**, **L2**, **1**, **2**, **1­(THF)**_**2**_ **.Na**, **1.Na**, **1.Mg**, **2.Na**, **2.Mg** and preparation of π-cation radicals thereof. NMR spectroscopy and NMR titrations data, electronic absorption spectroscopy data, infrared spectroscopy data, mass spectrometry, electron paramagnetic resonance spectroscopy, kinetics measurements and analysis, electrochemistry data. Computational methods and data (PDF)
