# Supplementary material for: Electrostatic Fields Induce Accelerated Proton Coupled Electron Transfer Rates in Chlorophyll Model Compounds
Source: J Am Chem Soc. 2025 Jul 29;147(32):29399–412. doi: 10.1021/jacs.5c09511 (PMC12356590; doi:10.1021/jacs.5c09511)
Supplement: Supplementary file 1 [file ja5c09511_si_001.pdf]

Supporting Information for:

Electrostatic Fields Induce Accelerated Proton Coupled Electron Transfer Rates in Chlorophyll Model Compounds

Oscar Reid Kelly,[a] Brendan Twamley,[a] Marcel Swart,[b,c] Aidan R. McDonald[a,*]

[a] School of Chemistry, Trinity College Dublin, the University of Dublin, College Green, Dublin 2, Ireland

[b] IQCC and Department of Chemistry, Universitat de Girona, 17003 Girona, Spain

[c] ICREA, Pg. Lluís Companys 23, 08010 Barcelona, Spain

Email: aidan.mcdonald@tcd.ie

Physical Methods

Handling of air-sensitive materials was performed using standard Schlenk techniques or under a nitrogen atmosphere inside an MBraun Labmaster Pro SP inert atmosphere glovebox.

^{1}H, ^{13}C, ^{19}F, and ^{31}P nuclear magnetic resonance (NMR) spectra were measured using an Agilent MR spectrometer or a Bruker AV400 spectrometer. ^{1}H and ^{19}F NMR spectra were referenced using the residual solvent peak and 1,2-difluorobenzene as internal standards, respectively.

Electrospray ionization mass spectrometry (ESI-MS) was acquired using a Bruker microTOF-Q III spectrometer in positive mode interfaced to a Dionex UltiMate 3000 LC. To obtain the ESI-MS of **3** the sample introduced to the spectrometer by direct injection (bypassing the LC).

Fourier transform infra-red (FT-IR) spectra were measured on neat samples using a Perkin Elmer Spectrum 100 FT-IR/ATR instrument. The FT-IR spectrum of **3** was obtained by drop casting a solution of the compound in CH_3CN onto the crystal and evaporating the solvent with a stream of nitrogen.

Electronic absorption spectra were obtained using an Agilent 8453 diode array spectrophotometer attached to a Unisoko Scientific Instruments cryostat cooled with liquid nitrogen.

Cyclic voltammetry experiments were conducted on a CH Instruments Model 600E Series potentiostat.

Single crystal X-ray diffraction (SC-XRD) data were collected from a shock-cooled single crystal at 100(2) K on a Bruker APEX2 Kappa Duo Kappa diffractometer with a microfocus sealed X-ray tube using mirror optics as a monochromator and an APEX2 detector. The diffractometer was equipped with a low temperature device and used Cu Kα radiation (λ = 1.54178 Å). All data were integrated with SAINT and a multi-scan absorption correction using

SADABS was applied.[1-2] The structure was solved by dual methods using XT and refined by full-matrix least-squares methods against F2 by XL using Olex2.[3-5] All non-hydrogen atoms were refined with anisotropic displacement parameters. All C-bound hydrogen atoms were refined isotropic on calculated positions using a riding model with their U_{iso} values constrained to 1.5 times the U_{eq} of their pivot atoms for terminal sp^3 carbon atoms and 1.2 times for all other carbon atoms. Disordered moieties were refined using bond lengths restraints and displacement parameter restraints. Crystallographic data for the structures reported here have been deposited with the Cambridge Crystallographic Data Centre.[6] CCDC 2390925-2390926 contain the supplementary crystallographic data for this paper. These data can be obtained free of charge from The Cambridge Crystallographic Data Centre via www.ccdc.cam.ac.uk/structures. This report was generated using FinalCif.[7]

Electron paramagnetic resonance (EPR) spectra were obtained on a Bruker EMX X-band EPR spectrometer equipped with a cold finger liquid nitrogen dewar. Samples were measured as frozen CH_3CN solutions (77 K) with a microwave power of 0.2 mW, microwave frequency of 9.2 GHz and modulation amplitude of 0.3 mT. Simulation of the EPR spectra was performed using EasySpin as implemented in MATLAB.[8] The systems were modelled as powder samples with $S = ½$, isotropic g-tensors and homogeneous line broadening.

Gas chromatography experiments were performed using a ThermoFisher TRACE™ 1300 Gas Chromatograph equipped with a Flame Ionization Detector (FID) and a ThermoFisher Trace GOLD TG-1MS GC column. Hydrogen was provided by a Parker Hydrogen Gas Generator 20H-MD. Air was provided by Parker Zero Air Generator UHP-10ZA-S. The instrument method for the detection/quantification of 2,6-di-*tert*-butylbenzoquinone was based on a temperature ramp (2 min. at 75 °C, 10 °C/min. to 200 °C, 2 min. at 250 °C) with splitless injections of 1 µL each. The product was quantified with reference to a calibration curve with

naphthalene as an internal standard. Under these conditions, the retention times for naphthalene and 2,6-di-*tert*-butylbenzoquinone were between 8.6-8.7 min. and 12.4-12.5 min., respectively.

The Density Functional Theory (DFT) calculations were performed with Orca 6.0, using the B97-3c functional in combination with PCM-COSMO (dichloromethane).[9-11] All species were optimized considering 2 axially ligated H_2O ligands at the porphyrin-bound Mg ion. The corresponding data have been uploaded onto the iochem-bd server: https://doi.org/10.19061/iochem-bd-4-83.

Materials

All materials were purchased from commercial vendors and used as received unless stated otherwise. 2-(tosyloxymethyl)-15-crown-5,[12] **L1**,[13] 5-(2,6-dihydroxyphenyl)-10,15,20-tritolylporphyrin,[14] $[N(p\text{-tol})_3][SbCl_6]$,[15] and $[Ru(bipy)_3](PF_6)_3$[16] were synthesized according to literature methods. ^{2}H-*O*-2,6-di-*tert*-butyl-4-methoxyphenol (4-CH_3O-2,6-DTBP-OD) was synthesized according to the literature and the degree of deuteration was assessed by ^{1}H NMR (96%, Figure S62).[17] Anhydrous N,N-dimethylformamide (DMF) was purchased from Sigma Aldrich and dispensed under a nitrogen atmosphere inside an MBraun Labmaster Pro SP inert atmosphere glovebox. Anhydrous CH_3CN was dispensed through an Innovative Technology PureSolv EN solvent purification system. Chromatography was performed on silica gel (0.06-0.200 nm, 60 Å).

Warning: **Perchlorate salts are explosive.** Although no detonations were observed during the course of this work, experiments were performed using the minimum quantity of perchlorate salt possible (not exceeding 500 mg) and heating/crushing/scraping of the salts was strictly avoided in order to mitigate risk.

Methods

General procedure for cyclic voltammetry experiments:

Cyclic voltammograms were obtained on 0.6 mM CH_3CN solutions of analyte in 0.1 M $N(^nBu)_4PF_6$ using a glassy carbon working electrode and platinum wire counter/reference electrodes at a scan rate of 0.2 V/s unless otherwise stated. All measurements were internally referenced to the decamethylferrocene/decamethylferrocenium cation redox couple ($DmFc/DmFc^+$) and reported against the ferrocene/ferrocenium cation redox couple (Fc/Fc^+) using the literature value for the $DmFc/DmFc^+$ couple.[18] Stoichiometric quantities of perchlorate salts were introduced as concentrated solutions in CH_3CN.

Synthesis and Characterization:

Aquomagnesium(II) tetra(p-tolyl)porphyrin, [Mg(H_2O)(TTP)]:

[Mg(H_2O)(TTP)] was synthesized using a modified literature procedure.[19] To a solution of 5,10,15,20-tetra(*p*-tolyl)porphyrin (H_2TTP) (200 mg, 0.3 mmol) in CH_2Cl_2 (20 mL) was added $MgBr_2.OEt_2$ (760 mg, 2.9 mmol) at 20 °C with stirring, giving a color change from red to green. Triethylamine (NEt_3, 0.8 mL) was then added dropwise, giving a color change to purple. After stirring for 2 h the reaction mixture was diluted with CH_2Cl_2 (50 mL) and washed with saturated $NaHCO_3$ solution (3 x 100 mL). The CH_2Cl_2 solution was then dried over $MgSO_4$. After passing the crude mixture through a silica gel plug (eluting with CH_2Cl_2) purification was achieved by column chromatography on alumina. Eluting with pure CH_2Cl_2 yielded the unreacted ligand first. Eluting then with 10% CH_3OH in CH_2Cl_2 gave the desired product, which was evaporated to dryness in vacuo to yield [Mg(H_2O)(TTP)] as a purple powder (202 mg; 98%).

^1H NMR ($CDCl_3$, 400 MHz) δ = 8.86 (8H, s, pyrrole C−H), 8.09 (8H, d, *J* = 8.0 Hz, *meta*-phenyl), 7.53 (8H, d, *J* = 8 Hz, *ortho*-phenyl), 2.71 (12H, s, *para*-tolyl CH_3), 1.00 (2H, br s, H_2O) ppm.

$^{13}C\{^1H\}$ NMR ($CDCl_3$, 101 MHz) δ = 150.0, 141.1, 136.4, 134.6, 131.6, 127.0 (C_β), 121.3 (C_{meso}), 21.5 (*para*-tolyl *C*H_3) ppm

ESI-MS: found *m/z* = 692.2771 ($[M]^+$, $C_{48}H_{36}MgN$ requires 692.2790).

ν (ATR-FTIR, cm^{-1}): 3570, 3507 (m, H_2O), 3022-2867 (w, C−H), 1590, 1517, 1480 (w, C=C), 1332, 1250, 1202, 1108, 1070, 1069 (m, C−O), 997, 973, 846, 790, 721 (s, C−H).

Electronic absorption (CH_3CN, 20 °C) λ / nm (ε / M^{-1}) = 402 (50700), 422 (362000), 564 (16000), 604 (9680).

5-(2-((1,4,7,10,13-pentaoxacyclopentadecan-2-yl)methoxy)phenyl)-10,15,20-tri-p-tolylporphyrin, ***L1****:*

L1 was synthesized according to a literature procedure.[13]

^1H NMR ($CDCl_3$, 400 MHz) δ = 8.86 (8H, m, $C_\beta H$), 8.12-8.07 (7H, m, *meta*-tolyl and *para*-phenyl), 7.76 (1H, t, *J* = 8.0 Hz, *meta*-phenyl), 7.57 (6H, m, *ortho*-tolyl), 7.39 (1H, t, *J* = 8 Hz, *meta*-phenyl), 7.30 (1H, d, *J* = 8.0 Hz, *meta*-phenyl), 3.92 (2H, d, *J* = 4 Hz, Ar−O−CH_2−R), 2.87 (1H, m, 15-c-5 C*H*), 2.71 (9H, s, *para*-tolyl CH_3), 2.69-2.19 (18H, m, 15-c-5) and -2.74 (2H, s, pyrrole N*H*) ppm.

$^{13}C\{^1H\}$ NMR ($CDCl_3$, 101 MHz) δ = 158.7, 139.3, 139.2, 137.3, 134.6, 134.5 (*para*-tolyl *ortho*- and *meta*-C), 131.3, 130.9 (C_β), 129.9 (*meta*-phenyl), 127.4 (*para*-tolyl *ortho*- and *meta*-

C), 119.8 (C_{meso}), 119.7 (*meta*-phenyl), 115.9, 111.8 (*ortho*-phenyl), 69.6, 69.5, 69.4, 69.3, 69.2, 68.8, 68.5 (15-crown-5 CH_2, *C*H), 21.5 (*para*-tolyl CH_3) ppm

ESI-MS: found *m/z* = 927.4082 ($[M + Na]^+$, $C_{58}H_{56}N_4O_6Na$ requires 927.4092).

ν (ATR-FTIR, cm^{-1}): 3318 (w, N−H), 3121, 3023 (w, C−H), 2920, 2867 (m, C−H), 1561, 1472 (w-m, C=C), 1349, 1248, 1114 (m-s, C−O), 966, 800, 736 (s, C−H).

Electronic absorption (CH_3CN, 20 °C) λ / nm (ε / M^{-1}) = 370 (20700), 416 (346200), 513 (17700), 548 (8700), 590 (5100), 646 (5100).

5-(2,6-bis((1,4,7,10,13-pentaoxacyclopentadecan-2-yl)methoxy)phenyl)-10,15,20-tri-p-tolylporphyrin, ***L2****:*

L2 was synthesized using a modification of the literature method for the synthesis of **L1**.[13] 5,10,15-tritolyl-20-(2,6-dihydroxyphenyl)porphyrin (144 mg, 0.20 mmol) was dissolved in DMF (50 mL) under a nitrogen atmosphere. With stirring, NaH (60% dispersion in paraffin, 20 mg, 0.48 mmol) was then added followed by 2-(tosyloxymethyl)-15-crown-5 (200 mg, 0.49 mmol) dropwise as a solution in minimal DMF. The reaction mixture was then heated to 70 °C under nitrogen for 30 h, after which it was diluted with dilute aqueous HCl (100 mL) and extracted with CH_2Cl_2 until the aqueous layer was colorless (3 x 100 mL). The combined CH_2Cl_2 extracts were then washed with water and dilute ammonia solution. The CH_2Cl_2 layer was then evaporated, diluted with diethylether (100 mL) and washed three times with water (100 mL). The Et_2O solution was then dried over $MgSO_4$ and adsorbed on silica. The product was purified by column chromatography on silica gel (5% CH_3OH in $CHCl_3$) and dried *in vacuo* to yield **L2** as a purple powder (122 mg, 53%).

^1H NMR ($CDCl_3$, 400 MHz) δ = 8.82 (8H, d, *J* = 9.2 Hz, $C_\beta H$), 8.07 (6H, m, *meta*-tolyl), 7.70 (1H, t, *J* = 8.0 Hz, *para*-phenyl), 7.55 (6H, m, *ortho*-tolyl), 7.00 (2H, d, *J* = 8.0 Hz, *meta*-

phenyl), 3.92 (4H, m, Ar−O−*CH*$_2$−R), 2.87 (2H, m, 15-c-5 C*H*), 2.71 (9H, s, *para*-tolyl), 2.67-2.38 (21H, m, 15-c-5), 2.33-2.07 (13H, m, 15-c-5), 1.96 (2H, m, 15-c-5) and -2.74 (2H, s, pyrrole N−H) ppm.

$^{13}C\{^1H\}$ NMR ($CDCl_3$, 101 MHz) δ = 159.5, 139.3, 139.1, 137.4, 134.4 (*para*-tolyl *ortho*- and *meta*-C), 130.4 (*para*-phenyl), 127.5, 127.4 (*para*-tolyl *ortho*- and *meta*-C), 119.4 (C_{meso}), 105.2 (*meta*-phenyl), 70.4, 70.0, 69.2, 68.6, 68.4 (15-crown-5 *C*H$_2$, *C*H), 21.5 (*para*-tolyl *C*H$_3$) ppm

ESI-MS: found *m/z* = 599.2614 ($[M + 2Na]^{2+}$, $C_{69}H_{76}N_4O_{12}Na_2$ requires 599.2622), 1153.5538 ($[M + H]^+$, $C_{69}H_{77}N_4O_{12}$ requires 1153.5533) and 1175.5353 ([M+Na]+, $C_{69}H_{76}N_4O_{12}Na$ requires 1175.5352).

ν (ATR-FTIR, cm^{-1}): 3317 (w, N−H), 3023-2860 (w-m, C−H), 1586, 1508, 1448, 1402 (w-m, C=C), 1349, 1250, 1222, 1184, 1097 (m-s, C−O), 991, 981, 965 (s, C−H).

Electronic absorption (CH_3CN, 20 °C) λ / nm (ε / M^{-1}) = 368 (19000), 416 (338000), 513 (17000), 548 (8000), 590 (4900), 646 (4000).

Diaquomagnesium(II) 5-(2-((1,4,7,10,13-pentaoxacyclopentadecan-2-yl)methoxy)phenyl)-10,15,20-tri-p-tolylporphyrin, ***1****:*

To **L1 (**125 mg, 0.138 mol) in CH_2Cl_2 (10 mL) was added $MgBr_2.OEt_2$ (350 mg, 1.36 mol) at 20°C with stirring, giving a color change from red to green. Triethylamine (NEt_3, 0.5 mL) was then added dropwise, giving a color change to purple. After stirring for 1 hour the crude reaction mixture was adsorbed on silica and purified by column chromatography on silica gel. Eluting with CH_2Cl_2 gave first unreacted ligand. Eluting then with 10% CH_3OH in CH_2Cl_2 gave the desired product, which was evaporated to dryness *in vacuo* to yield **1** as a purple powder (104 mg; 81%).

^{1}H NMR ($CDCl_3$, 400 MHz) δ = 8.80-8.66 (8H, m, pyrrole $C_{\beta}H$), 8.44 (1H, dd, J_1 = 8.0 Hz, J_2 = 1.6 Hz, *meta*-phenyl), 8.11-7.92 (6H, m, *meta*-tolyl), 7.67 (1H, td, J_1 = 8.0 Hz, J_2 = 1.6 Hz, *para*-phenyl), 7.53 (7H, m, *ortho*-tolyl and *meta*-phenyl), 6.98 (1H, d, *J* = 8.0 Hz, *ortho*-phenyl), 3.39 (2H, dd, J_1 = 32 Hz, J_2 = 8 Hz, Ar−O−CH_2−R), 2.71 (9H, s, *para*-tolyl), 2.25-1.61 (13H, m, 15-c-5), 1.49 (4H, br s, H_2O), 1.14 (1H, br s, 15-c-5), 0.89 (2H, br s, 15-c-5) and 0.66-0.23 (3H, m, 15-c-5) ppm

$^{13}C\{^{1}H\}$ NMR ($CDCl_3$, 101 MHz) δ = 159.60, 150.48, 150.37, 150.09, 149.95, 149.84, 149.79, 141.42, 136.54, 136.52, 135.47, 135.36, 135.30, 134.89, 134.46, 134.32 (*para*-tolyl *ortho*- and *meta*-C), 132.88, 131.88, 131.65, 131.61, 131.55, 131.39, 131.24, 131.07 (C_{β}), 129.35 (*meta*-phenyl), 127.37, 126.95 (*para*-tolyl *ortho*- and *meta*-C), 121.50, 121.36, 121.12, 120.54 (C_{meso}), 116.39, 112.87 (*ortho*-phenyl), 69.05, 68.16, 67.88, 67.64, 67.42, 67.37, 66.20 (15-crown-5 *C*H_2, *C*H), 21.64 (*para*-tolyl *C*H_3) ppm

ESI-MS: found *m/z* = 927.3948 ([M+H]+, $C_{58}H_{55}MgN_4O_6$ requires 927.3972), and 949.3757 ([M+Na]+, $C_{58}H_{54}MgN_4O_6Na$ requires 949.3791).

ν (ATR-FTIR, cm^{-1}): 3364 (br, H_2O), 3022-2886 (w, C−H), 1520, 1480, 1446 (w, C=C), 1334, 1250, 1201, 1181, 1107, 1064 (m, C−O), 994 (s, C−H).

Electronic absorption (CH_3CN, 20 °C) λ / nm (ε / M^{-1}) = 403 (44100), 424 (348000), 564 (15100), 604 (9450).

Diaquomagnesium(II) 5-(2,6-bis((1,4,7,10,13-pentaoxacyclopentadecan-2-yl)methoxy)phenyl)-10,15,20-tri-p-tolylporphyrin, ***2****:*

To **L2** (60 mg, 52 mmol) in CH_2Cl_2 (10 mL) was added $MgBr_2.OEt_2$ (140 mg, 542 mmol) at 20 °C with stirring, giving a color change from red to green. NEt_3 (0.2 mL) was then added dropwise, giving a color change to purple. After stirring for 1 h the crude reaction mixture was

adsorbed on silica and purified by column chromatography on silica gel (10% CH_3OH in CH_2Cl_2) to yield the desired product as the first fraction, which was dried *in vacuo* to yield **2** as a purple powder (48 mg ; 78%).

^{1}H NMR ($CDCl_3$, 400 MHz) δ = 8.84-8.70 (8H, m, pyrrole C−H), 8.10-8.00 (6H, m, *ortho*-tolyl), 7.64 (1H, t, *J* = 8.3 Hz, *para*-phenyl), 7.54 (6H, m, *meta*-tolyl), 6.94 (2H, d, *J* = 8.1 Hz, *meta*-phenyl), 3.83 (4H, m, Ar−O−CH_2−R), 2.80 (3H, m, 15-c-5), 2.71 (9H, s, *para*-tolyl) and 2.65-1.64 (35H, m, 15-c-5), 1.48 (4H, br s, H_2O) ppm.

^{13}C{^{1}H} NMR ($CDCl_3$, 101 MHz) δ = 149.8, 149.6, 141.2, 136.4, 134.8, 134.6, 134.6 (*para*-tolyl *ortho*- and *meta*-C), 131.7, 131.2 ($C_β$), 129.7 (*para*-phenyl), 127.0 (*para*-tolyl *ortho*- and *meta*-C), 120.7 (C_{meso}), 106.2 (*meta*-phenyl), 68.5, 68.1, 67.9, 67.3 (15-crown-5 CH_2, *C*H), 21.5 (*para*-tolyl CH_3) ppm

ESI-MS: found *m/z* = 610.2445 ([M+2Na]2+, $C_{69}H_{74}MgN_4O_{12}Na_2$ requires 610.2469) and 1197.5034 ([M+Na]+, $C_{69}H_{74}MgN_4O_{12}Na$ requires 1197.5046).

ν (ATR-FTIR, cm^{-1}): 3349 (br, H_2O), 3022-2850 (w-m, C−H), 1668, 1585, 1522, 1451 (m, C=C), 1353, 1332, 1293, 1251, 1201, 1182, 1104 (m-s, C−O), 994 (s, C−H).

Electronic absorption (CH_3CN, 20 °C) λ / nm (ε / M^{-1}) = 404 (43500), 424 (336000), 564 (15100), 604 (8070).

2,6-di-tert-butyl-1,4-dimethoxybenzene

This compound was synthesized using a procedure analogous to that reported for the preparation of 2,4,6-tri-*tert*-butylanisol.[20] 2,6-di-*tert*-butyl-4-methoxyphenol (450 mg, 1.9 mmol) was dissolved in anhydrous THF (20 mL) and degassed by sparging with Ar for 15 minutes. The solution was then cooled to 0 °C in an ice bath before NaH (60% dispersion in

paraffin, 100 mg, 2.4 mmol) was added. The mixture was allowed to stir for 1 h at 20 °C before CH_3I (0.13 mL, 2.1 mmol) was added dropwise. The flask was wrapped in aluminium foil and allowed to react at 20 °C in the dark overnight before ethanol (2 mL) was added dropwise to quench excess NaH. The solvent was removed in vacuo and purification was achieved by column chromatography (2% ethylacetate in hexane) to afford the title compound as an orange liquid. ^{1}H NMR data closely matched literature reports (Figure S63).[21]

^{1}H NMR ($CDCl_3$, 400 MHz) δ = 6.82 (s, 2H, aromatic C*H*), 3.80 (s, 3H, OC*H₃*), 3.69 (s, 3H, OC*H₃*), 1.45 (s, 18H, *tert*-butyl C*H₃*) ppm.

*Generation of Na^+ and Mg^{2+} adducts of **1** and **2**, preparation of **1(THF)₂.Na**:*

General: The equilibrium between the crown ether substituents and Na^+ in CH_3CN rendered the isolation of the Na^+ adducts impractical, as dissolution of the isolated 1:1 adduct in CH_3CN would result in release of Na^+. For consistency, all cation adducts of **1** and **2** were therefore generated *in-situ* for analysis by UV-Vis, ^{1}H NMR, and cyclic voltammetry, using excess salt as needed to push the equilibrium to the cation-bound species (see below for detailed procedures).

The exception to this is compound **$1(THF)_2.Na$**, which was isolated as single crystals for SC-XRD by the following method: **1** (10 mg, 0.01 mol) was added to a 4 mL vial and dissolved in minimal THF that had been pre-saturated with $NaClO_4$ (approx. 0.2 mL). Vapor diffusion of pentane into this solution overnight at 20 °C yielded purple crystals of **$1(THF)_2.Na$** along with colorless crystals of $NaClO_4$. A purple crystal of **$1(THF)_2.Na$** was isolated manually with a needle for analysis by SC-XRD.

*Procedure for in-situ generation of **1.Na**, **1.Mg**, **2.Na** and **2.Mg** for electrochemical analysis:*

1.Na: To a 2 mL solution of **1** (0.6 mM, CH_3CN) containing $N(^nBu)_4PF_6$ (0.1 M) was added 80 μL of a $NaClO_4$ solution (60 mM, CH_3CN, 4 equiv.) with stirring. Electrochemical measurements were then performed directly on this sample without stirring and without further purification.

1.Mg: To a 2 mL solution of **1** (0.6 mM, CH_3CN) containing $N(^nBu)_4PF_6$ (0.1 M) was added 20 μL of a $Mg(ClO_4)_2$ solution (60 mM, CH_3CN, 1 equiv.) with stirring. Electrochemical measurements were then performed directly on this sample without stirring and without further purification.

2.Na: To a 2 mL solution of **2** (0.6 mM, CH_3CN) containing $N(^nBu)_4PF_6$ (0.1 M) was added 160 μL of a $NaClO_4$ solution (60 mM, CH_3CN, 8 equiv.) with stirring. Electrochemical measurements were then performed directly on this sample without stirring and without further purification.

2.Mg: To a 2 mL solution of **2** (0.6 mM, CH_3CN) containing $N(^nBu)_4PF_6$ (0.1 M) was added 40 μL of a $Mg(ClO_4)_2$ solution (60 mM, CH_3CN, 1 equiv.) with stirring. Electrochemical measurements were then performed directly on this sample without stirring and without further purification.

*Procedure for in-situ generation of **1.Na** and **1.Mg** for ^1H NMR analysis:*

General: A solution of perchlorate salt ($NaClO_4$ or $Mg(ClO_4)_2$, 50 mM in perchlorate salt and 5 mM in **1**, 9.5% $CDCl_3$ in CD_3CN) was added in 10-50 μL aliquots to a solution of **1** (5 mM, 9.5% $CDCl_3$ in CD_3CN) at 20 °C. ^1H NMR spectra were collected after each addition. For

$NaClO_4$, the relevant chemical shifts were plotted as a function of [$NaClO_4$] and fitted to a 1:1 binding model *via* a global analysis.[22]

1.Na: To a solution of **1** (0.5 mL, 5 mM, 9.5% $CDCl_3$ in CD_3CN) in an NMR tube was successively added a solution of **1** and $NaClO_4$ combined (5 mM in **1** and 50 mM in $NaClO_4$) in the following volumes: 6 x 10 μL, followed by 2 x 20 μL, followed by 2 x 25 μL, followed by 1 x 50 μL (a total of 4 equivalents of $NaClO_4$). The solution was mixed by inverting the NMR tube after each addition and a ^1H NMR spectrum was collected between each addition.

1.Mg: To a solution of **1** (0.5 mL, 5 mM, 9.5% $CDCl_3$ in CD_3CN) in an NMR tube was successively added a solution of **1** and $NaClO_4$ combined (5 mM in **1** and 50 mM in $Mg(ClO_4)_2$) in the following volumes: 6 x 10 μL, followed by 2 x 20 μL, followed by 2 x 25 μL, followed by 1 x 50 μL (a total of 4 equivalents of $Mg(ClO_4)_2$). The solution was mixed by inverting the NMR tube after each addition and a ^1H NMR spectrum was collected between each addition.

Generation of π-cation radical complexes and reactivity studies:

Generation of $\mathbf{1}^{\bullet+}$ and $\mathbf{2}^{\bullet+}$ and reactivity studies were performed at 20 °C. For the generation of $\mathbf{1}^{\bullet+}$ and $\mathbf{2}^{\bullet+}$ for characterization, 1 equivalent of $[Ru(bipy)_3](PF_6)_3$, CAN, or $[N(p\text{-tol})_3]SbCl_6$ were added as an CH_3CN solution to **1** or **2** (0.6 mM). $[Ru(bipy)_3](PF_6)_3$ was used as the oxidant for the reactivity studies, which were performed using 60 μM solutions of **1** and **2**. Generation of $\mathbf{1}^{\bullet+}$ and $\mathbf{2}^{\bullet+}$ in the presence of $NaClO_4$ and $Mg(ClO_4)_2$ proceeded by first adding the salt to **1** or **2** as a solution in CH_3CN, followed by 1 equivalent of $[Ru(bipy)_3](PF_6)_3$. Substrate (varying volumes of a 120 mM solution) was added as a solution in CH_3CN to $\mathbf{1}^{\bullet+}$ and $\mathbf{2}^{\bullet+}$ and the reaction progress was followed by electronic absorption spectroscopy. Measurements of k_{obs} were

performed in triplicate by exponential fitting of the decay of the absorbance of **1**$^{•+}$ and **2**$^{•+}$ at λ = 362 and 364 nm, respectively. k_2 values were determined through linear fitting of k_{obs} against the concentration of substrate. See below for details on the kinetic analysis of the non-linear dependence of k_{obs} on [$Mg(ClO_4)_2$].

Kinetic analysis:

$$k_{\mathrm{obs}} = ({k_2}^{\mathrm{min}} + ({k_2}^{\mathrm{max}} - {k_2}^{\mathrm{min}}) \cdot \frac{[\mathrm{Mg}]^n}{K^n + [\mathrm{Mg}]^n})[\mathrm{S}] \quad \text{(Equation S1)}$$

Equation S1 was used to fit the dependence of k_{obs} (20 equivalents of phenol) on the concentration of $Mg(ClO_4)_2$. A value of n = 2 was returned by the nonlinear regression, reflecting the fact that 2 equivalents of $Mg(ClO_4)_2$ bind to **2**$^{•+}$ (see derivation below). Here, [S] is the substrate (phenol) concentration, [Mg] is the $Mg(ClO_4)_2$ concentration, k_2^{min} is the second-order rate constant measured in the absence of $Mg(ClO_4)_2$ and k_2^{max} is the theoretical k_2 value corresponding to infinite [Mg]. Note here the limiting behaviors of this function: at [S] = 0, k_{obs} = 0 (no reaction in the absence of phenol); at [Mg] = 0, $k_{obs} = k_2^{min}$[S] (Figure 4) and as [Mg] approaches infinity, k_{obs} approaches k_2^{max}[S] (Figure 5). Therefore, the equation predicts non-linear dependence of k_{obs} on [Mg] and linear dependence of k_{obs} on [S] at constant [Mg]. However, K^2 technically exhibits a dependence on [S] as shown in Equation S2:

$$K^2 = \frac{k_{-1} + {k_2}^{\mathrm{max}}[\mathrm{S}]}{k_1} \quad \text{(Equation S2)}$$

However, K^2 may be assumed to be constant if $k_1 >> k_2^{max}$[S] or if $k_{-1} >> k_2^{max}$[S], which would give K^2 a weak dependence on [S]. This is taken to be the case as a good fit for the data was returned assuming K^2 to be constant over the substrate concentration range employed. Therefore, K^2 is a measure of the apparent overall dissociation constant $K_d = k_{-1} / k_1$ for the

dissociation of two Mg^{2+} ions from $\mathbf{2}^{\bullet+}$. This value can then be used to calculate the apparent overall association constant $K_a = 1/K_d$. As we have considered the binding of two Mg^{2+} ions to $\mathbf{2}^{\bullet+}$ to occur in a single step in this analysis, the K_a thus derived will be the product of the association constants of the individual binding steps. The reported value is an estimate of the K_a for the association of *one* Mg^{2+} ion to $\mathbf{2}^{\bullet+}$, i.e., based on the above calculations using K rather than K^2. An estimation of the expected rate enhancement was made using the slope of the simplified Marcus equation, where k_{ab} is the electron transfer (ET) rate constant, k_{aa} and k_{bb} are self-exchange ET rate constants, ΔE^0 is the standard potential of the oxidant and f is a correction factor:

$$\log(k_{ab}) = 0.5\left(\log(k_{aa}) + \log(k_{bb}) + \frac{\mathrm{n}F}{\mathrm{RT}}\Delta E^0 + \log(f)\right) \text{ (Equation S3)}$$

Using Equation 1 (main text) we can express Equation S3 in terms of $\boldsymbol{q}$:

$$\log(k_{ab}) = 0.5\left(\log(k_{aa}) + \log(k_{bb}) + \frac{\mathrm{n}F}{\mathrm{RT}}\cdot\frac{\boldsymbol{q}}{4\pi\varepsilon r} + \log(f)\right) \text{ (Equation S4)}$$

Derivation of Equation S1:

Scheme S1. Simplified kinetic scheme used to derive Equation S1.

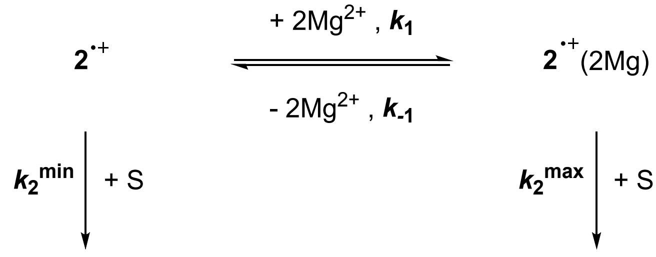

Equation S1 may be derived from Scheme S1 as follows, where S represents the phenol substrate and square brackets indicate the concentration of the enclosed species:

$$\text{Rate} = \frac{\text{d}[\mathbf{2}^{\bullet+}]_{\text{total}}}{\text{dt}} = k_2{}^{\text{min}}[\mathbf{2}^{\bullet+}][\text{S}] + k_2{}^{\text{max}}[\mathbf{2}^{\bullet+}(2\text{Mg})][\text{S}]$$

$$[\mathbf{2}^{\bullet+}]\text{total} = [\mathbf{2}^{\bullet+}] + [\mathbf{2}^{\bullet+}(2\text{Mg})]$$

$$\therefore\ \text{Rate} = k_2{}^{\text{min}}[\mathbf{2}^{\bullet+}]\text{total}[\text{S}] + (k_2{}^{\text{max}} - k_2{}^{\text{min}})[\mathbf{2}^{\bullet+}(2\text{Mg})][\text{S}]$$

$$\text{Under steady state}\left(\frac{\text{d}([\mathbf{2}^{\bullet+}(2\text{Mg})]}{\text{dt}} = 0\right)\text{: } [\mathbf{2}^{\bullet+}(2\text{Mg})] = \frac{[\mathbf{2}^{\bullet+}]_{\text{total}}[\text{Mg}]^2}{K^2 + [\text{Mg}]^2},\ K^2 = \frac{k_{-1} + k_{2\text{max}}[\text{S}]}{k_1}$$

$$\therefore\ \text{Rate} = k_2{}^{\text{min}}[\mathbf{2}^{\bullet+}]_{\text{total}}[\text{S}] + (k_2{}^{\text{max}} - k_2{}^{\text{min}})\frac{[\text{Mg}]^2}{K^2 + [\text{Mg}]^2}[\mathbf{2}^{\bullet+}]_{\text{total}}[\text{S}]$$

The equation takes the form: $\text{Rate} = k_2{}^{\text{apparent}}[\mathbf{2}^{\bullet+}]_{\text{total}}[\text{S}]$

Under pseudo first order conditions: $k_{\text{obs}} = k_2{}^{\text{apparent}}[\text{S}]$

$$\therefore\ k_{\text{obs}} = (k_2{}^{\text{min}} + (k_2{}^{\text{max}} - k_2{}^{\text{min}}) \cdot \frac{[\text{Mg}]^2}{K^2 + [\text{Mg}]^2})[\text{S}]$$

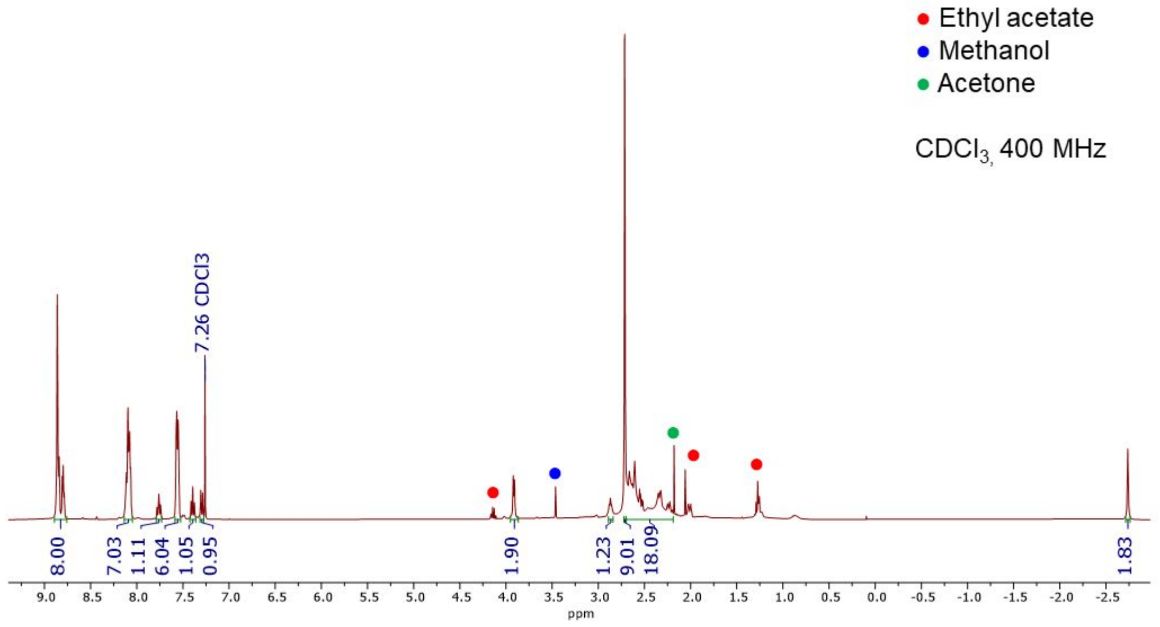

Figure S1. ^{1}H NMR spectrum of **L1**.

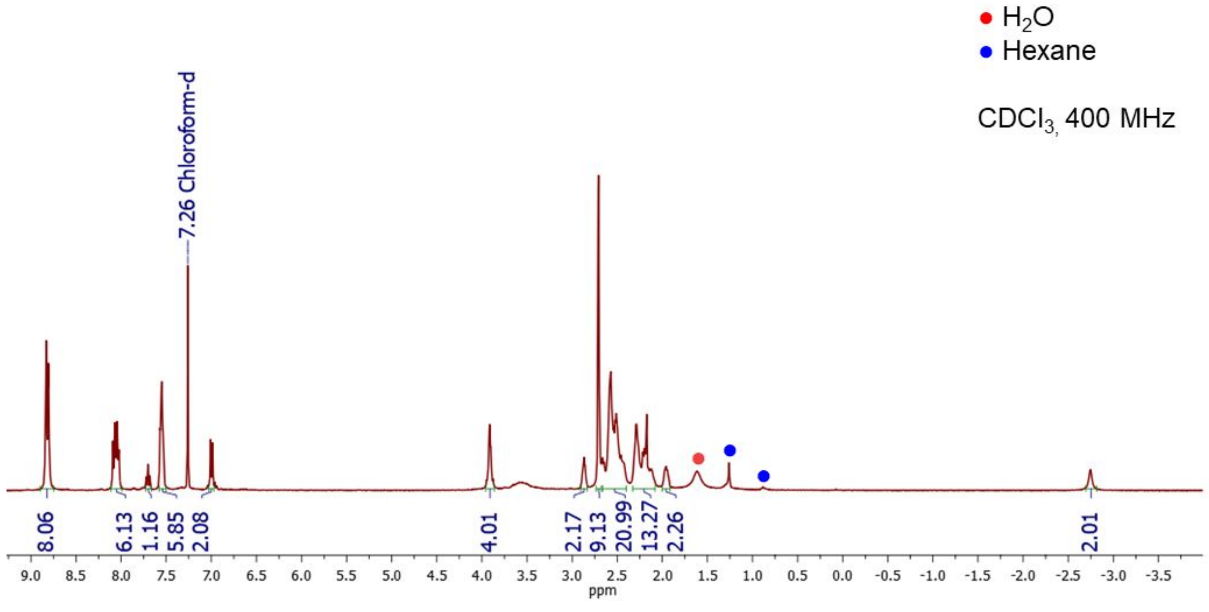

Figure S2. ^{1}H NMR spectrum of **L2**.

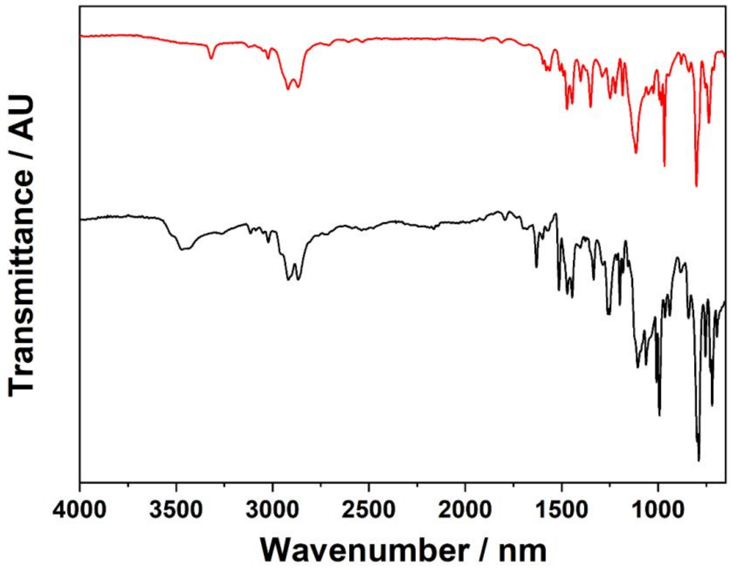

Figure S3. FT-IR spectrum of **L1** (red) and **1** (black).

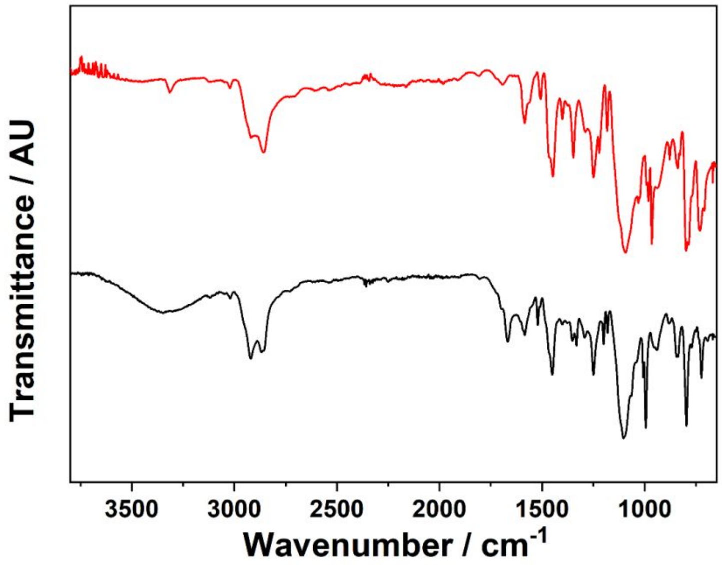

Figure S4. FT-IR spectrum of **L2** (red) and **2** (black).

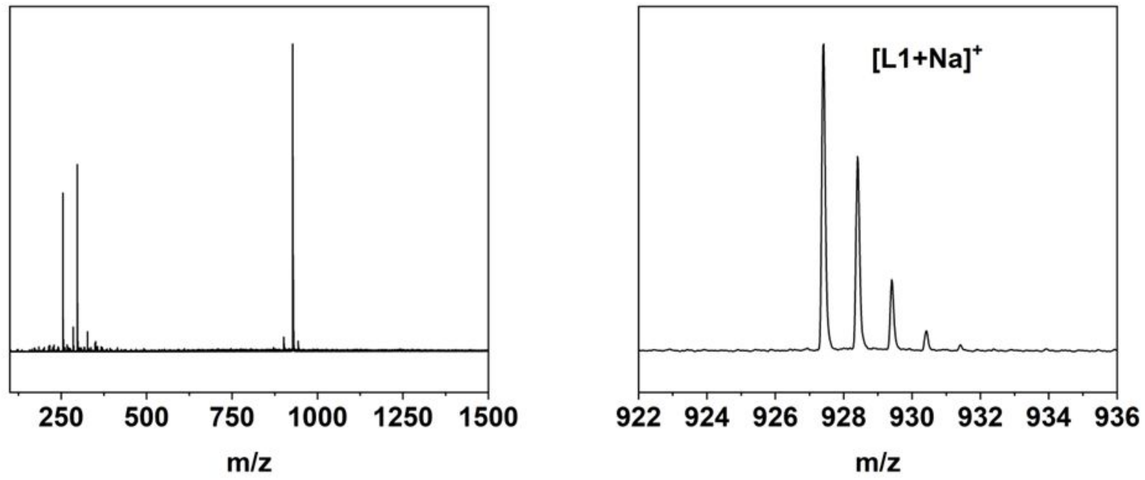

Figure S5. ESI-MS spectrum of **L1**.

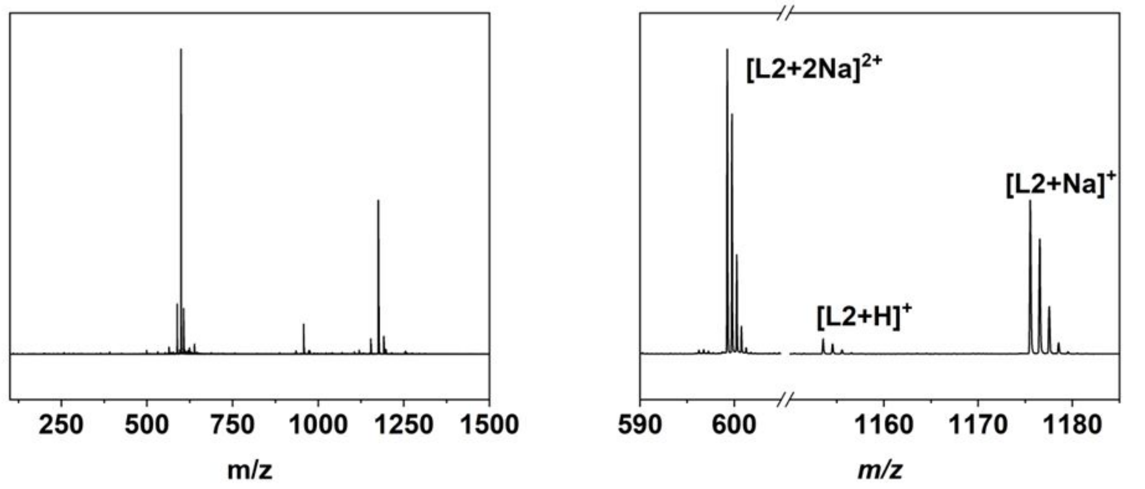

Figure S6. ESI-MS spectrum of **L2**.

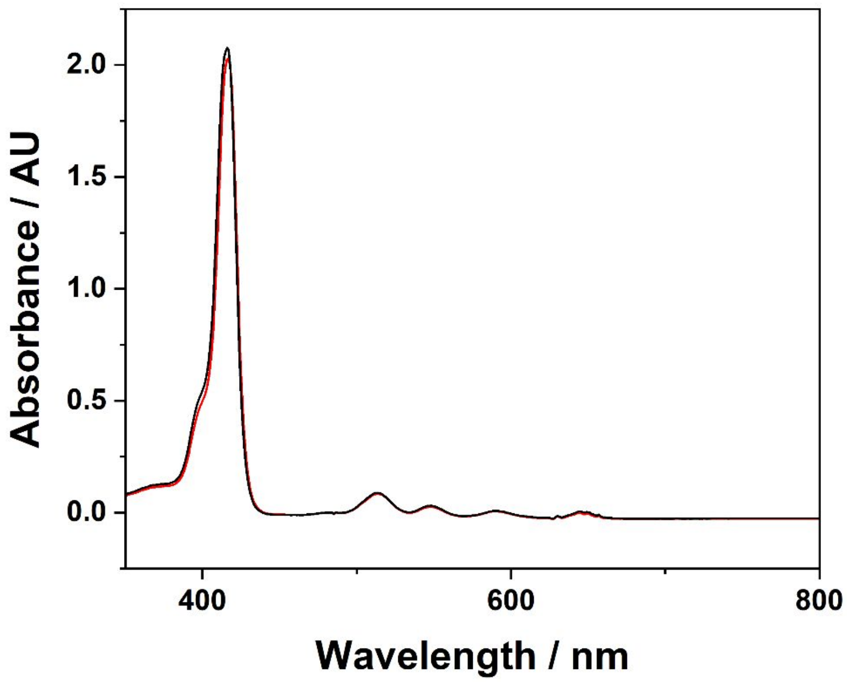

Figure S7. Electronic absorption spectrum of **L1** (black) and **L2** (red) (60 μM, 20 °C, CH_3CN).

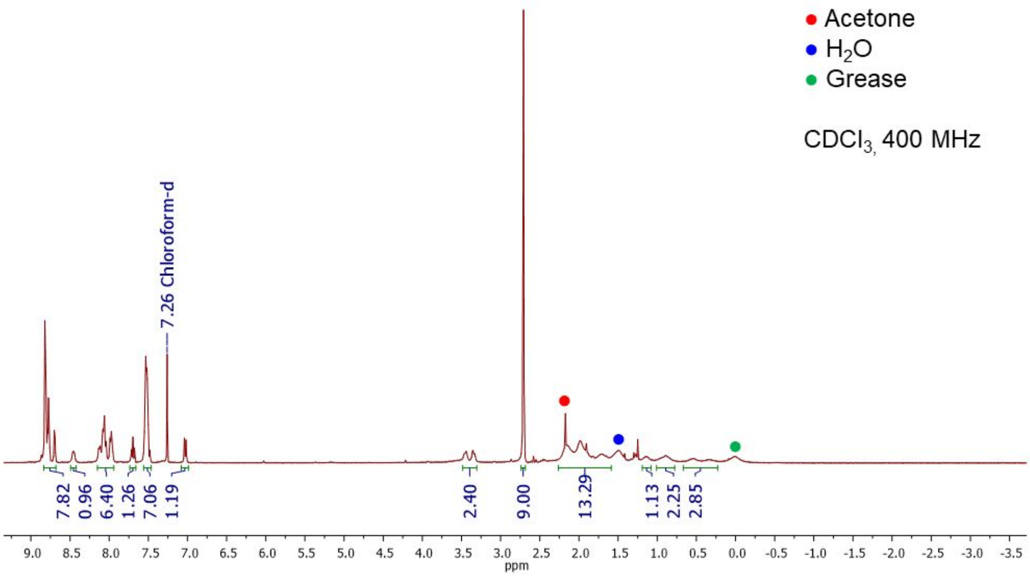

Figure S8. ^{1}H NMR spectrum of **1**.

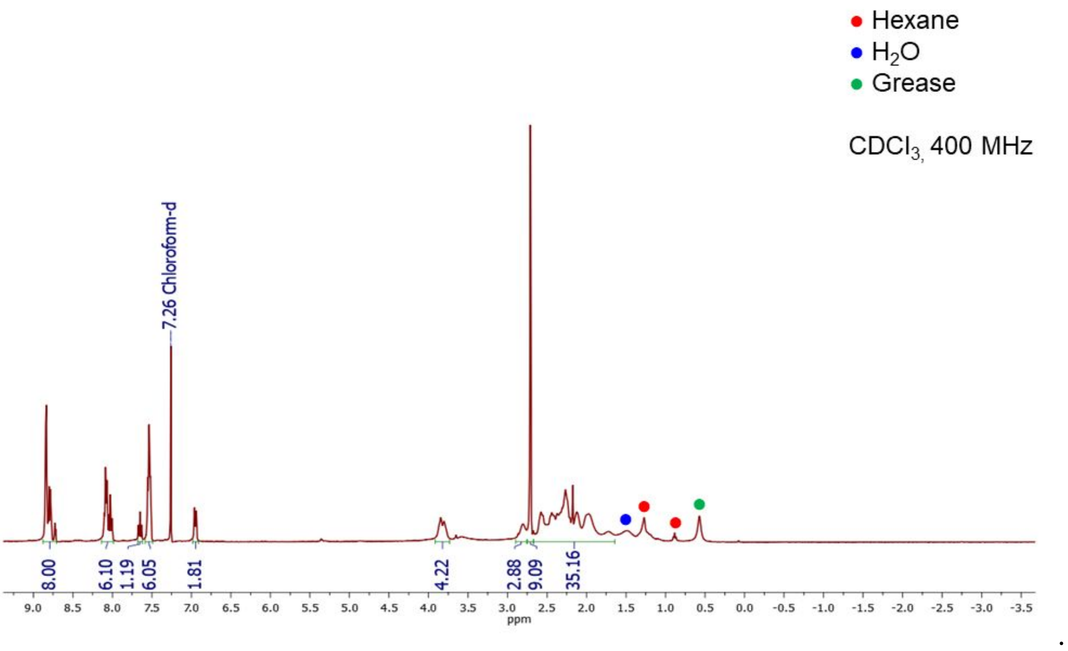

Figure S9. ^{1}H NMR spectrum of **2**.

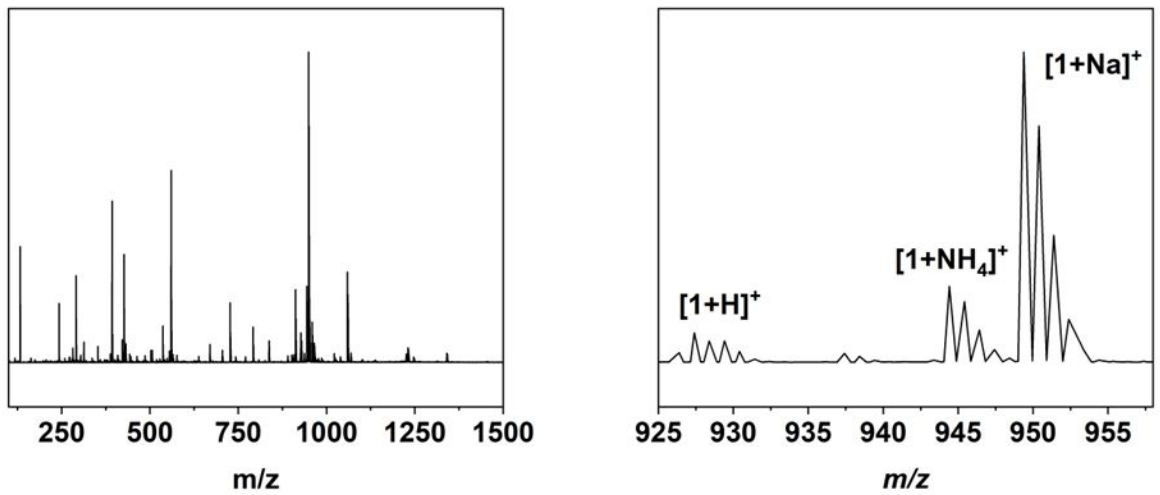

Figure S10. ESI-MS spectrum of **1**.

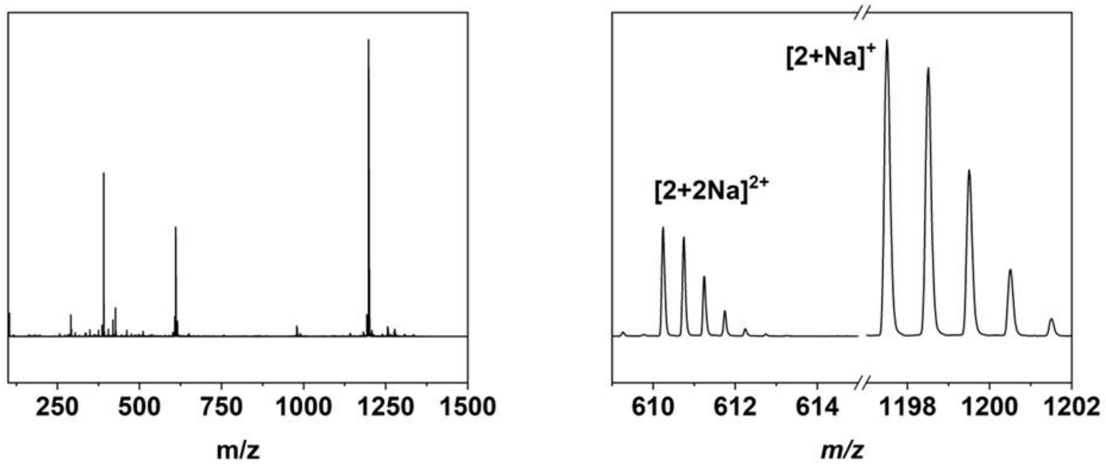

Figure S11. ESI-MS spectrum of **2**.

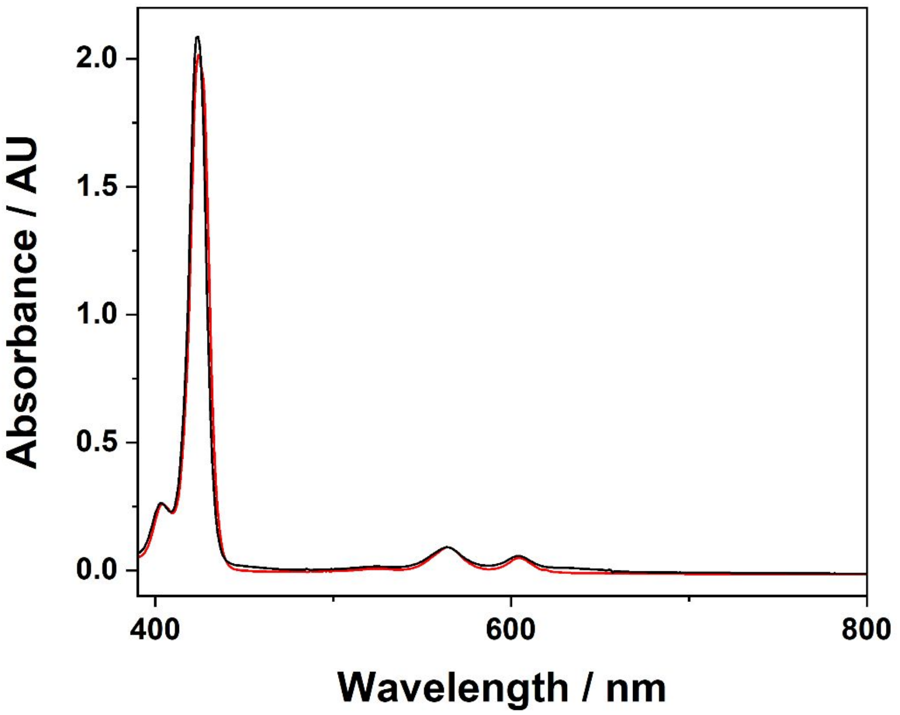

Figure S12. Electronic absorption spectra of **1** (black) and **2** (red) (6 µM, 20 °C, CH_3CN).

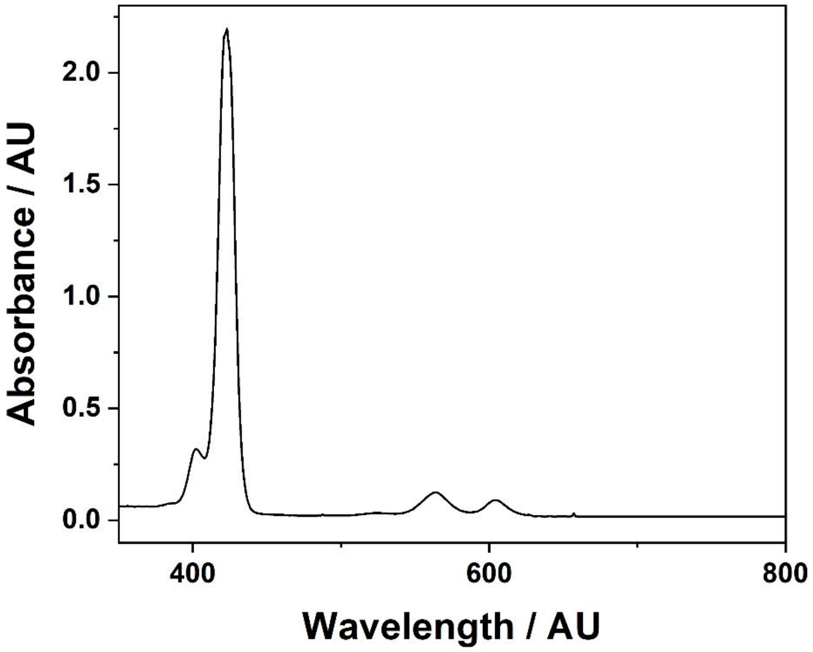

Figure S13. Electronic absorption spectrum of [Mg(H_2O)(TTP)] (6 μM, 20°C, CH_3CN).

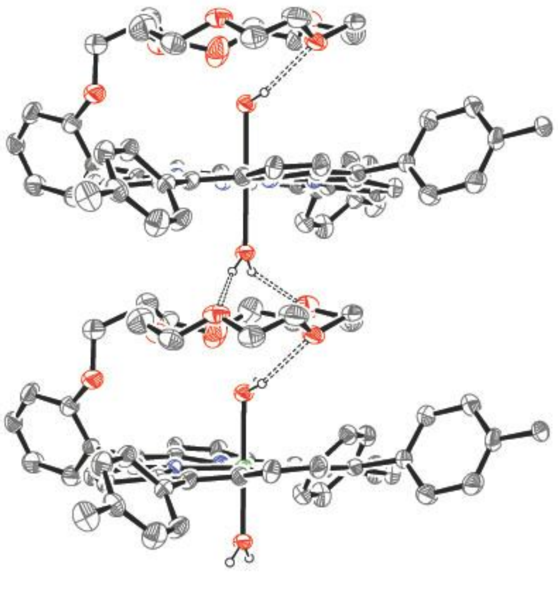

Figure S14. ORTEP of **1** drawn at 30% probability showing the intra- and intermolecular hydrogen bonding in the crystal. Water hydrogen atoms involved in H-bonding shown, all other hydrogens omitted with solvent of crystallization for clarity.

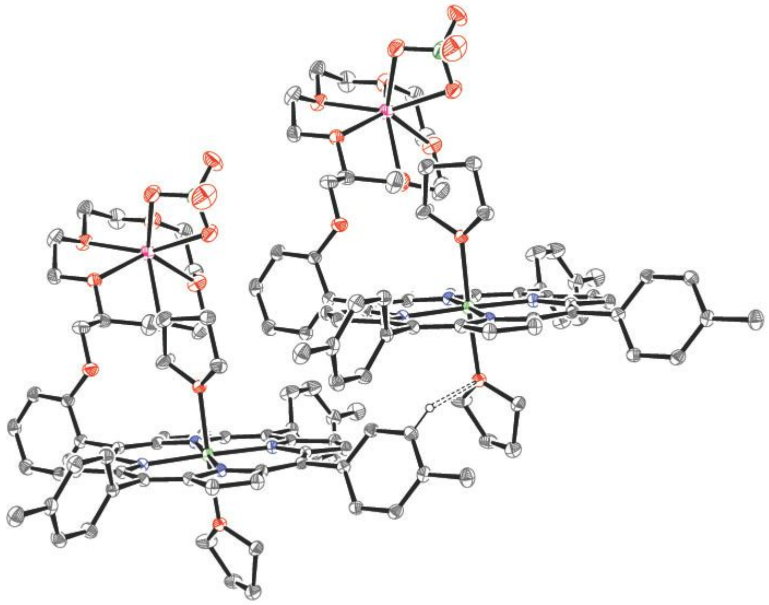

Figure S15. ORTEP of **1(THF)$_2$.Na** drawn at 30% probability showing intermolecular H-bonding in the crystal. Phenyl C-H involved in intermolecular H-bonding shown, all other hydrogens omitted with solvent of crystallization for clarity

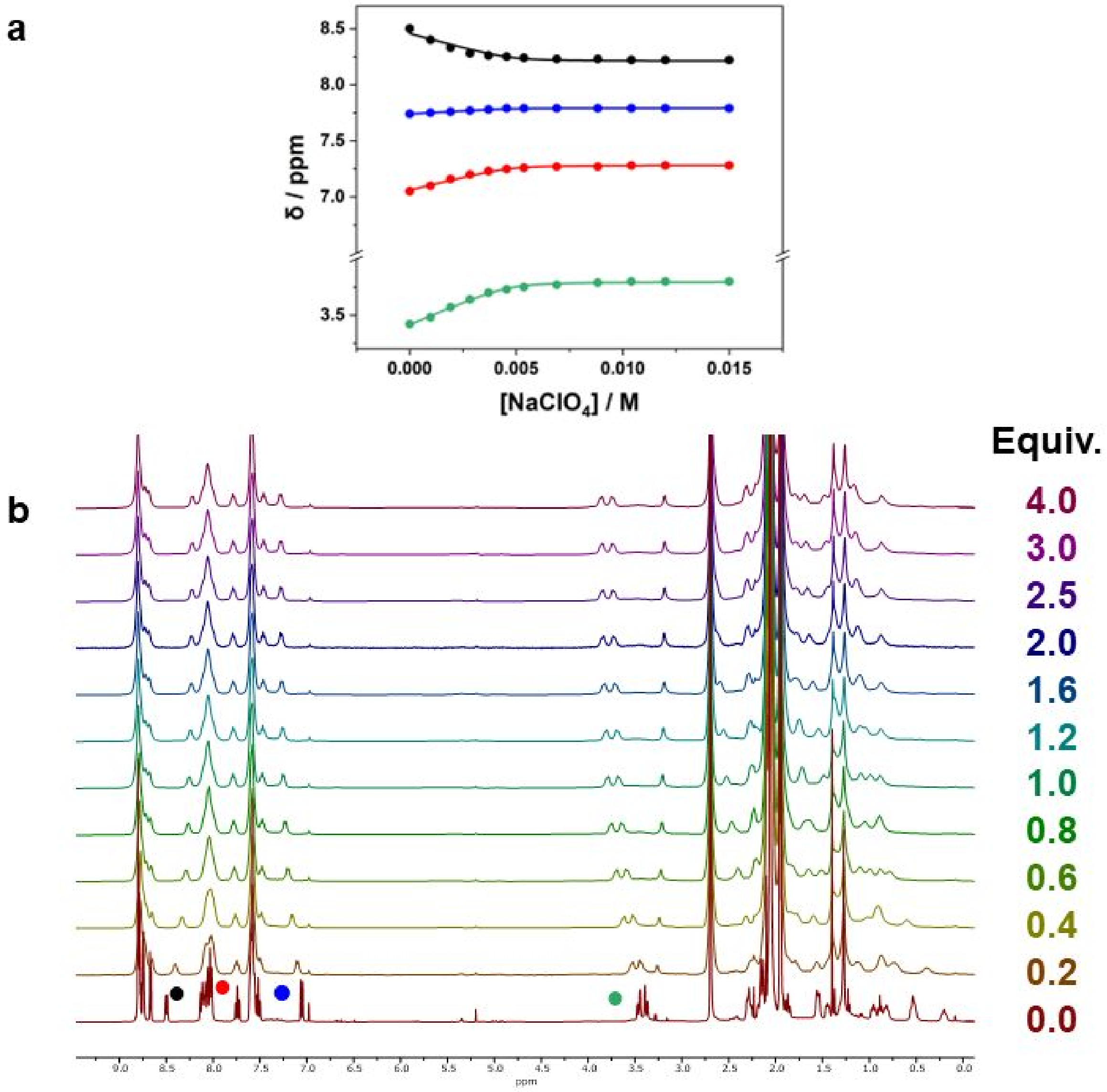

Figure S16. a) Global non-linear fitting of the changes to the chemical shifts at δ = 8.50, 7.74, 7.05 and 3.42 ppm upon titration of **1** against $NaClO_4$. b) Changes to the ^1H NMR spectra of **1** upon titration against $NaClO_4$. The peaks used in the non-linear fit are indicated with colored markers that correspond to the datapoints of the same color in a).

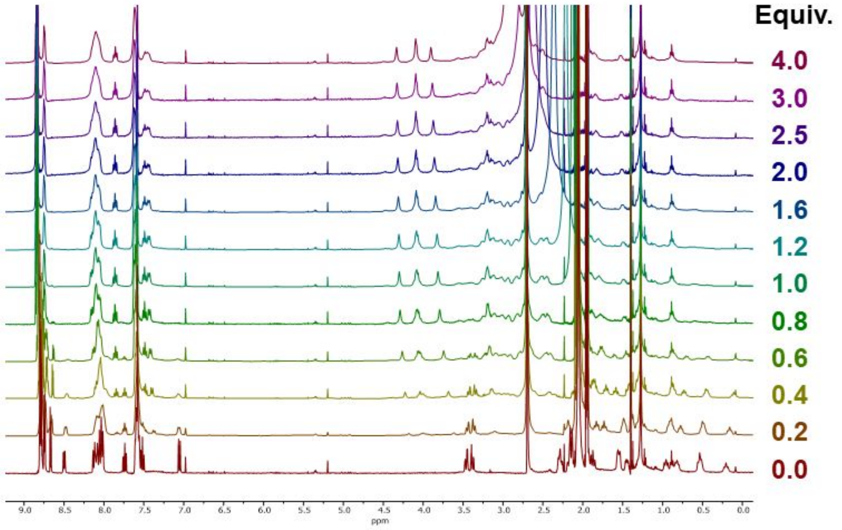

Figure S17. Changes to the ^{1}H NMR spectra of **1** upon titration against $Mg(ClO_4)_2$.

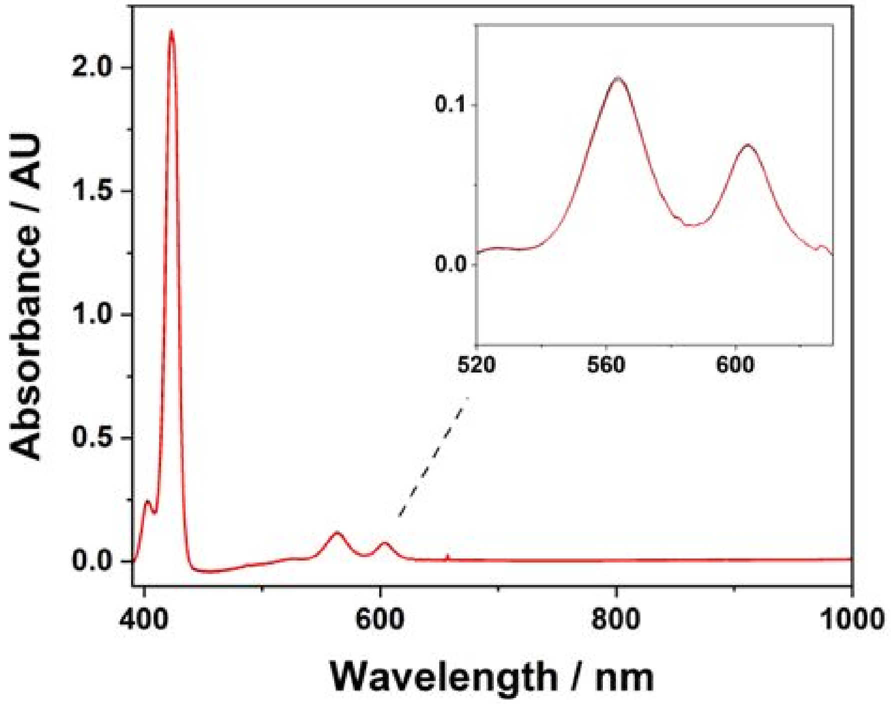

Figure S18. Electronic absorption spectra of **1** (black) and **1** upon addition of $NaClO_4$ (4 equiv., red trace).

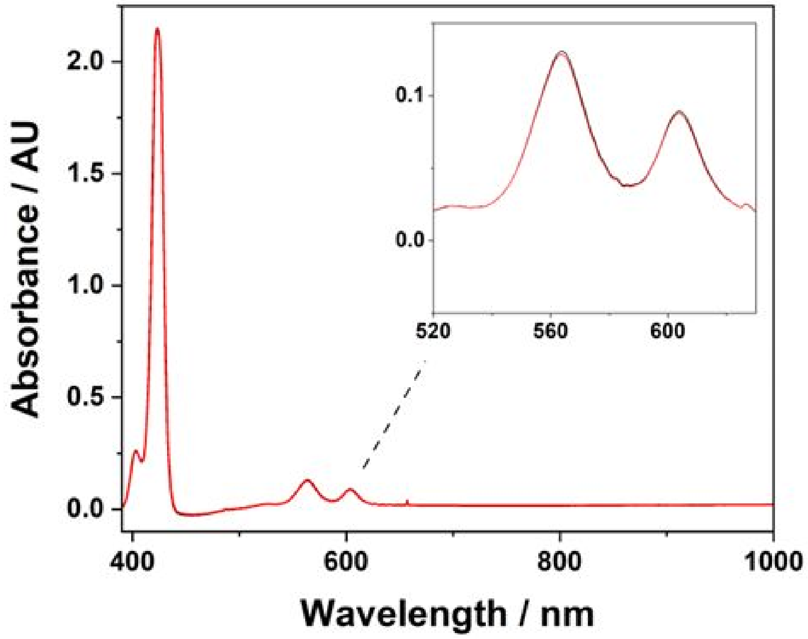

Figure S19. Electronic absorption spectra of **1** (black) and **1** upon addition of $Mg(ClO_4)_2$ (one equiv., red trace).

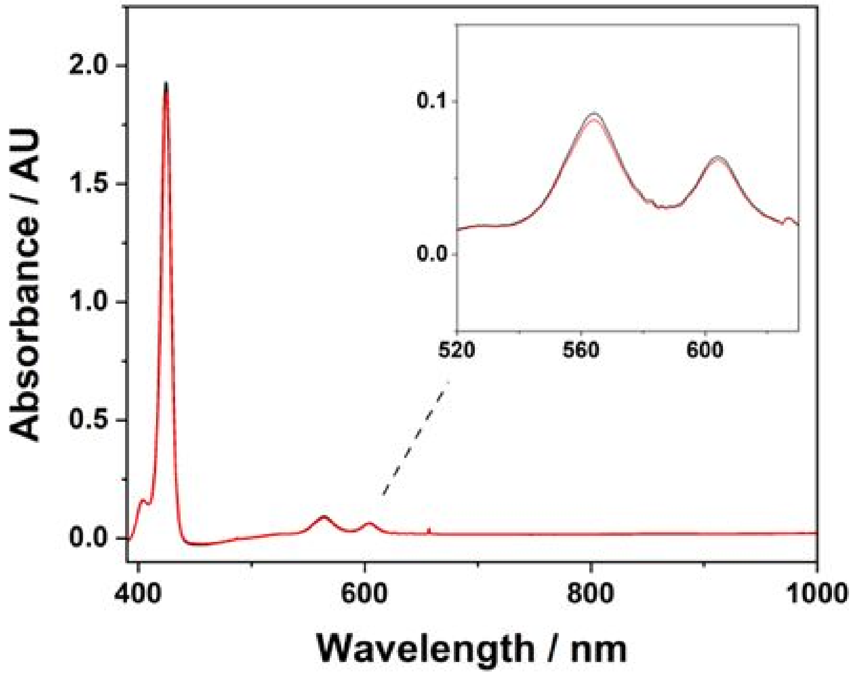

Figure S20. Electronic absorption spectra of **2** (black) and **2** upon addition of $NaClO_4$ (8 equiv., red trace).

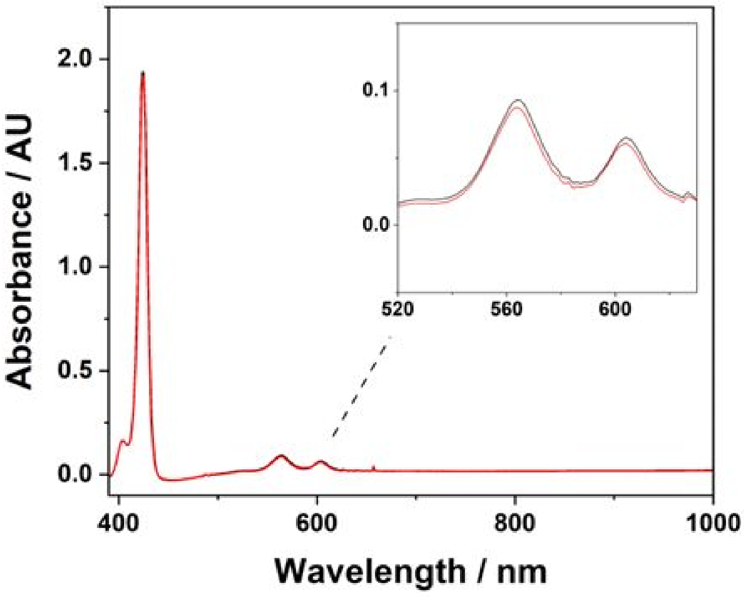

Figure S21. Electronic absorption spectra of **2** (black) and **2** upon addition of $Mg(ClO_4)_2$ (2 equiv., red trace).

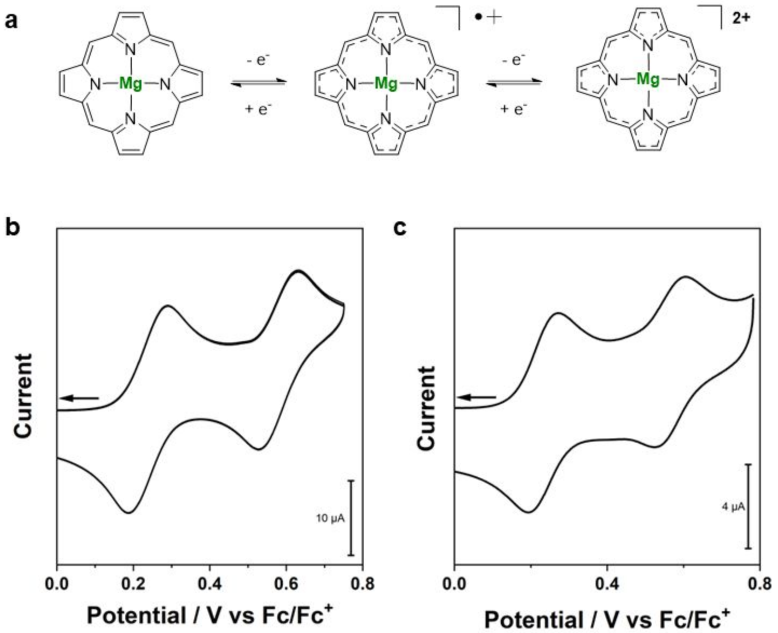

Figure S22. a) Scheme depicting the redox events observed in the cyclic voltammograms of b) **1** (0.6 mM, CH_3CN, RT) and c) **2** (0.6 mM, CH_3CN, RT). Scan rate = 0.2 V/s. The arrows indicate the direction of the potential sweep (cathodic).

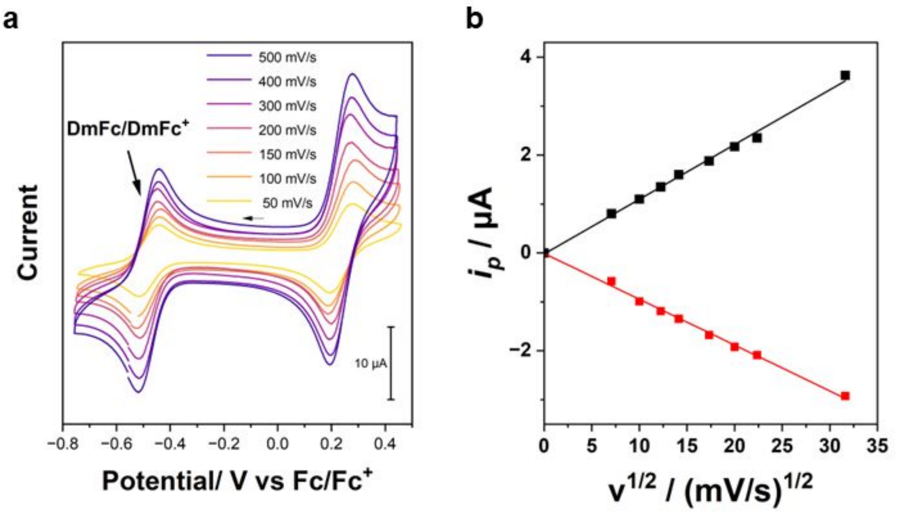

Figure S23. a) The first redox event of **1** measured at various scan rates. b) Randles-Sevic plot of the anodic (black) and cathodic (red) peak currents as a function of the square root of the scan rate.

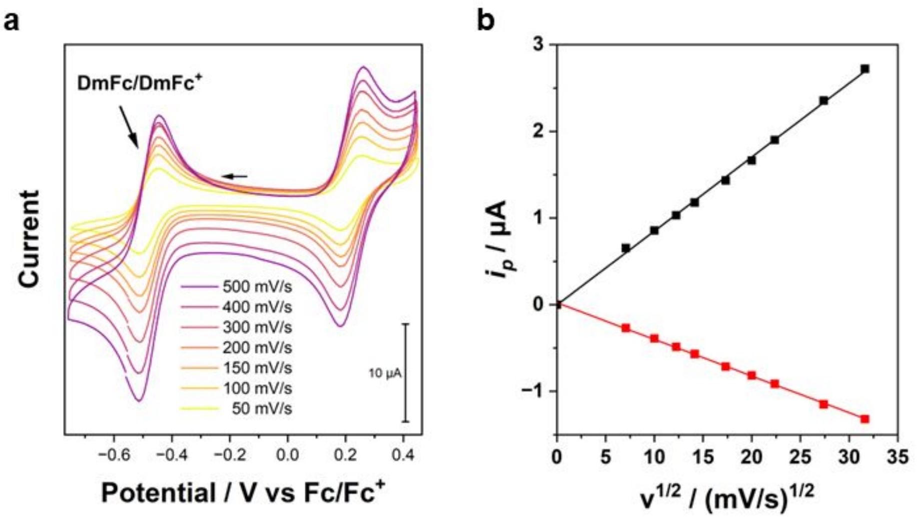

Figure S24. a) The first redox event of **2** measured at various scan rates. b) Randles-Sevic plot of the anodic (black) and cathodic (red) peak currents as a function of the square root of the scan rate.

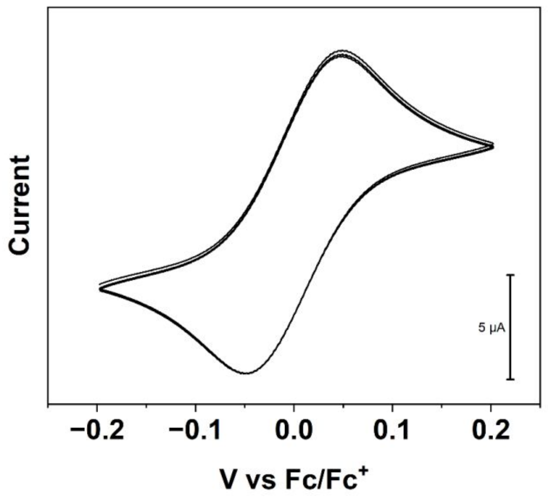

Figure S25. Cyclic voltammogram of ferrocene (Fc) showing the Fc/Fc^+ couple (0.6 mM, CH_3CN, RT).

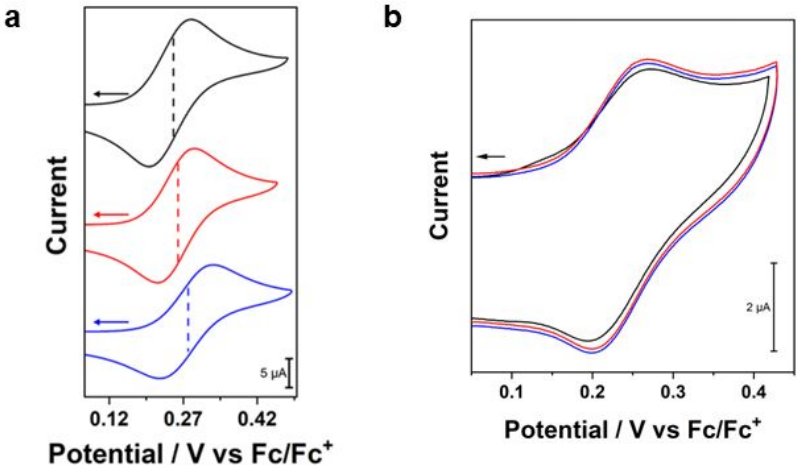

Figure S26. a) Cyclic voltammograms showing the first redox event of **1** (black), showing the shift in $E_{1/2}$ upon addition of $NaClO_4$ (red, 4 equiv.) and $Mg(ClO_4)_2$ (blue, 1 equiv.). b) Cyclic voltammograms showing the first redox event of $[Mg(H_2O)(TTP)]$ (black, 0.2 mM), showing the effect of adding $NaClO_4$ (red, 8 equiv.) and $Mg(ClO_4)_2$ (blue, 2 equiv.). 200 mV/s, 0.1 M NBu_4PF_6 in CH_3CN, RT. The arrows indicate the direction of the potential sweep (cathodic).

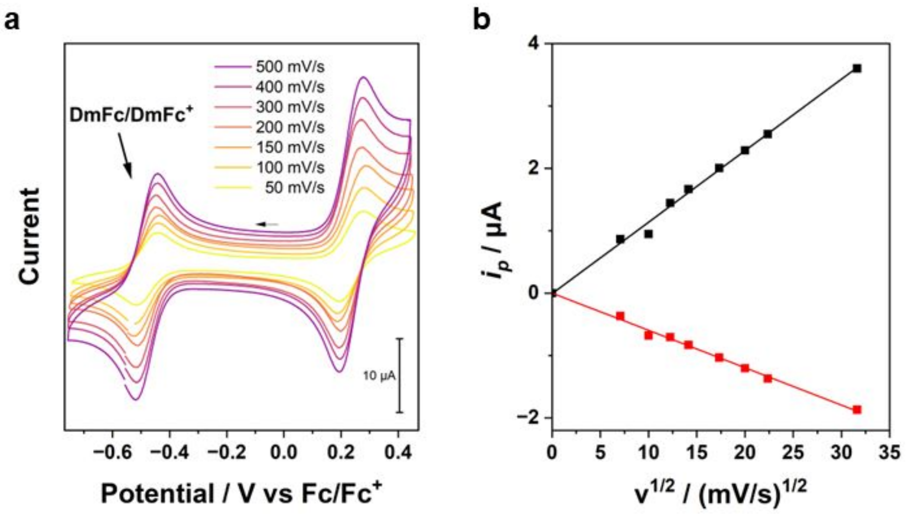

Figure S27. a) The first redox event of **1** in the presence of $NaClO_4$ (4 equiv.) measured at various scan rates. b) Randles-Sevic plot of the anodic (black) and cathodic (red) peak currents as a function of the square root of the scan rate.

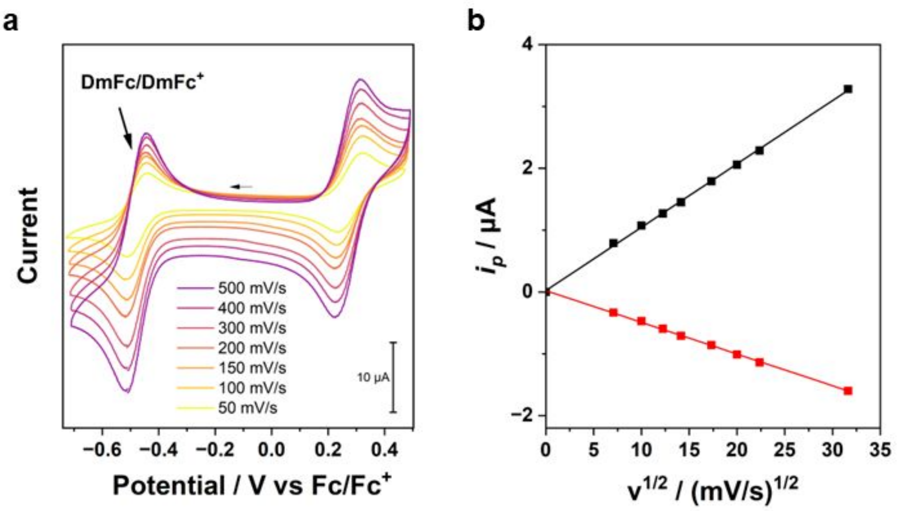

Figure S28. a) The first redox event of **1** in the presence of $Mg(ClO_4)_2$ (1 equiv.) measured at various scan rates. b) Randles-Sevic plot of the anodic (black) and cathodic (red) peak currents as a function of the square root of the scan rate.

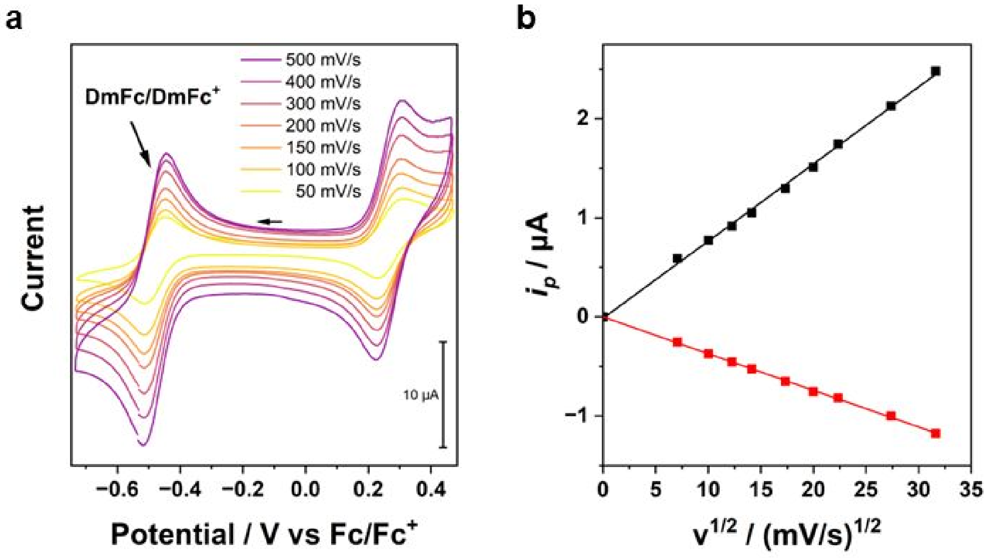

Figure S29. a) The first redox event of **2** in the presence of $NaClO_4$ (8 equiv.) measured at various scan rates. b) Randles-Sevic plot of the anodic (black) and cathodic (red) peak currents as a function of the square root of the scan rate.

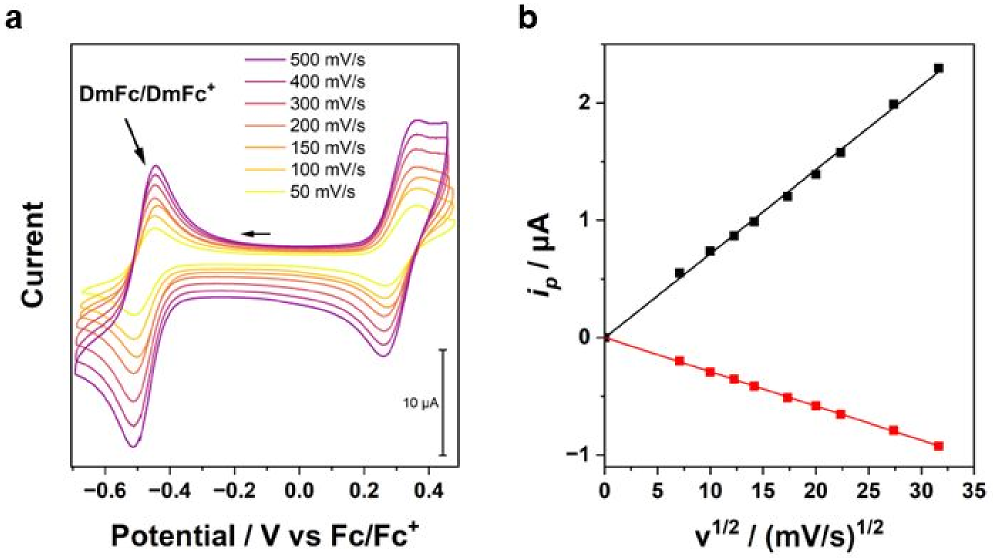

Figure S30. a) The first redox event of **2** in the presence of $Mg(ClO_4)_2$ (2 equiv.) measured at various scan rates. b) Randles-Sevic plot of the anodic (black) and cathodic (red) peak currents as a function of the square root of the scan rate.

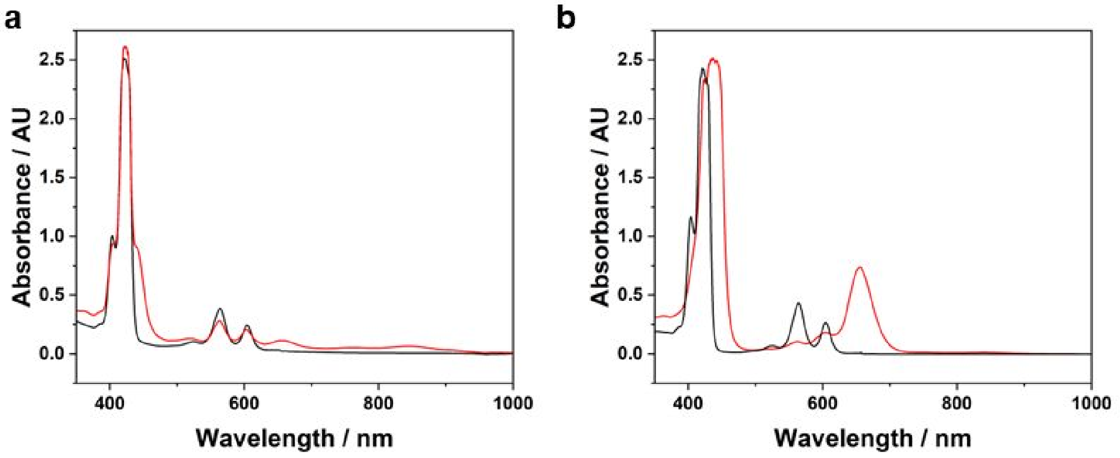

Figure S31. Electronic absorption spectra of **1** (black) upon addition of a) $Al(ClO_4)_3$ and b) $Sc(OTf)_3$ (red traces, CH_3CN, 20 °C).

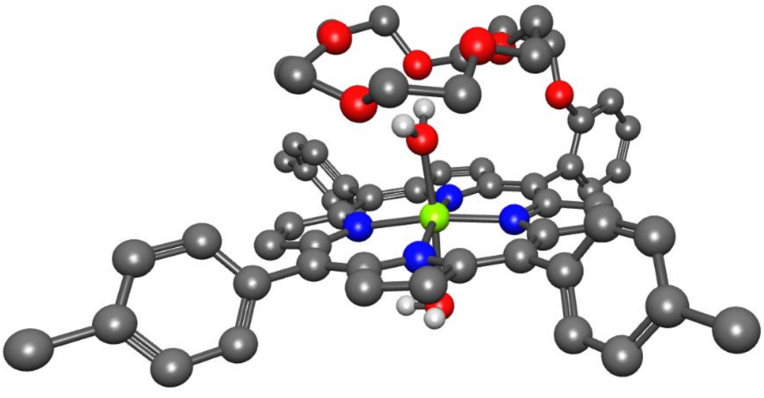

Figure S32. DFT-optimized structure of **1** (B97-3c).

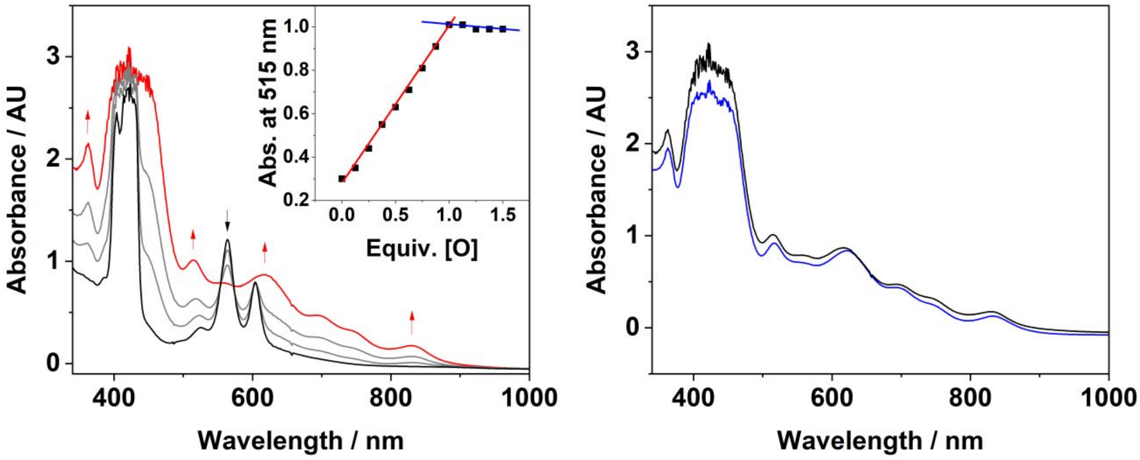

Figure S33. (Left) Electronic absorption spectra of **1** (black trace, CH_3CN, 60 μM) and new species $\mathbf{1}^{\bullet+}$ (red trace) formed upon addition of $[Ru(bpy)_3](PF_6)_3$ (1 equiv.) at 20 °C. Inset: Titration of **1** with sub-stoichiometric quantities of oxidant ([O]), showing maximum yield of $\mathbf{1}^{\bullet+}$ at one equivalent. (Right) Comparison between the electronic absorption spectra of $\mathbf{1}^{\bullet+}$ (black trace) and $\mathbf{2}^{\bullet+}$ (blue trace) generated with 1 equiv. $[Ru(bpy)_3](PF_6)_3$. Conditions: CH_3CN, 60 μM.

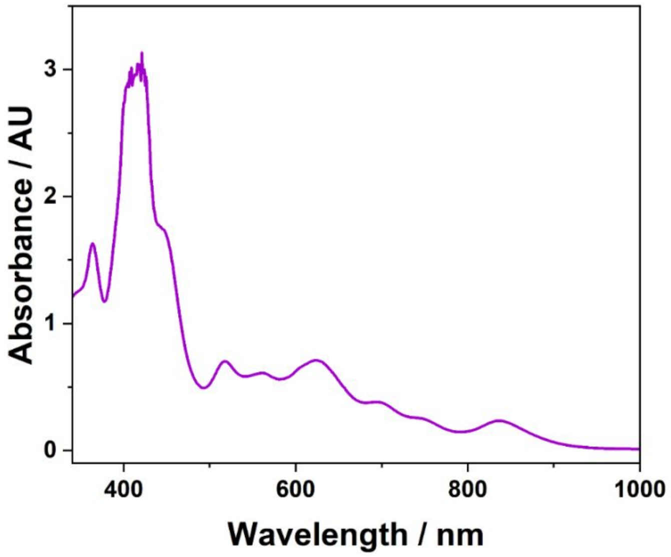

Figure S34. Electronic absorption spectrum of $\mathbf{2}^{\bullet+}$ (60 μM, CH_3CN, 20 °C) generated with CAN (1 equiv.).

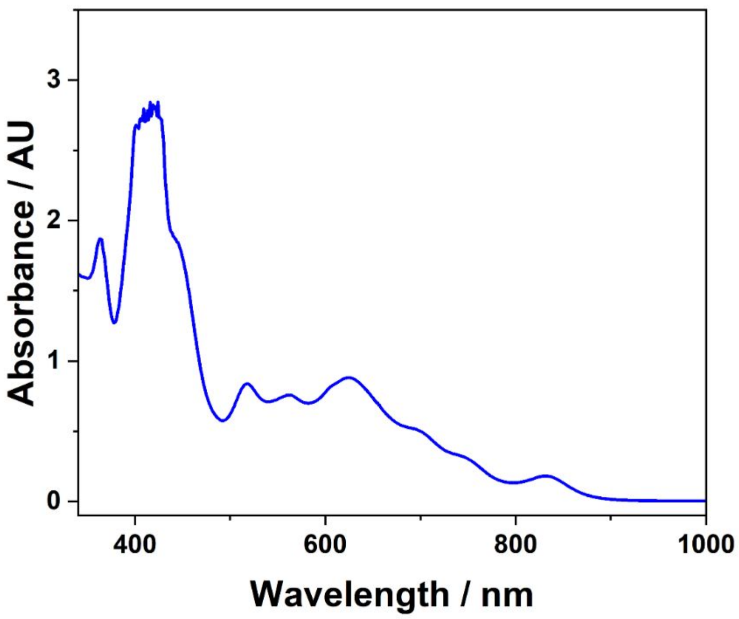

Figure S35. Electronic absorption spectrum of **2**$^{•+}$ (60 μM, CH_3CN, 20 °C) generated with $[N(p\text{-tol})_3]SbCl_6$ (1 equiv.).

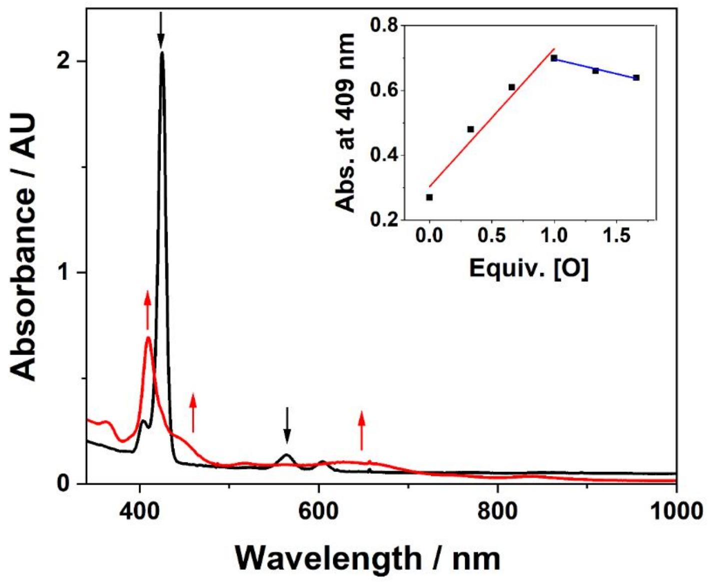

Figure S36. Titration of **2** (black trace) with sub-stoichiometric equivalents of $[N(p\text{-tol})_3]SbCl_6$ (CH_3CN, 6 μM, RT) showing a maximum yield of **2**$^{•+}$ (red trace) after 1 equivalent of oxidant ([O]) was added (inset).

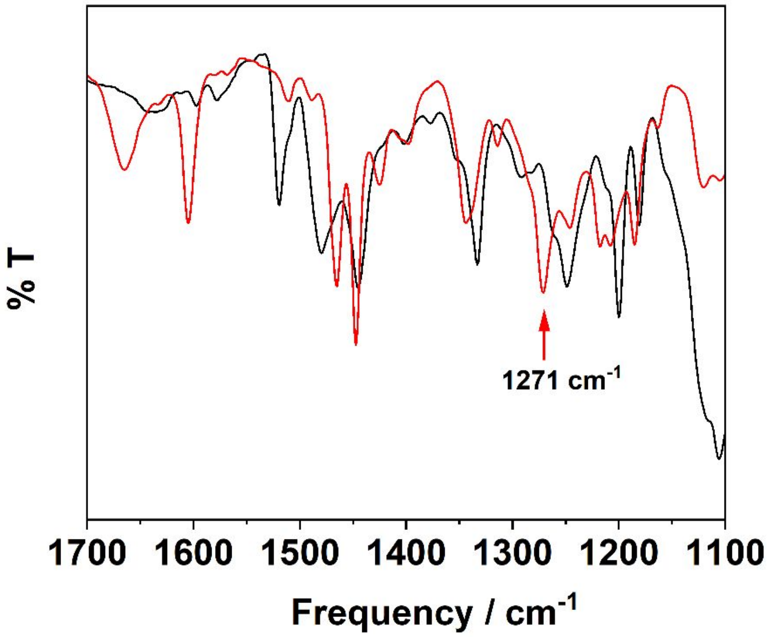

Figure S37. FT-IR spectrum of **1**$^{•+}$ generated with $[Ru(bpy)_3](PF_6)_3$ (red trace) compared with the FT-IR spectrum of **1** (black trace).

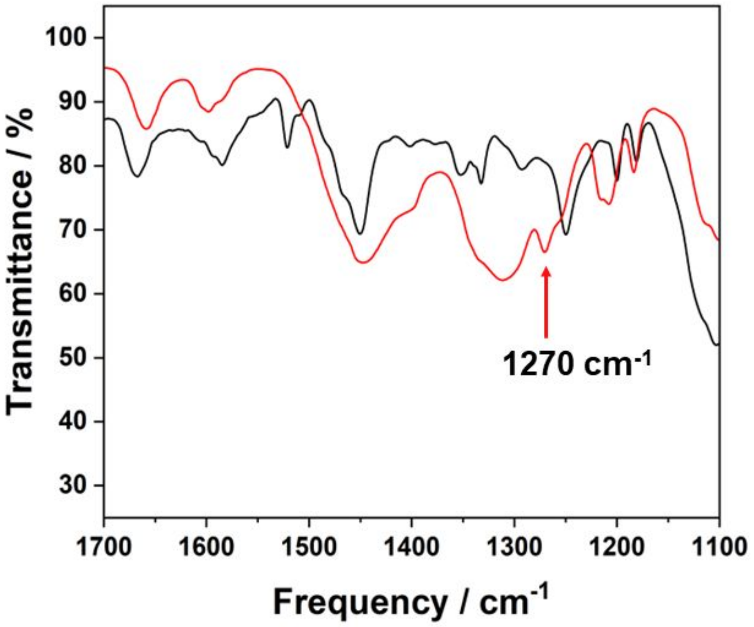

Figure S38. FT-IR spectrum of **2**$^{•+}$ generated with CAN (red trace) compared with the FT-IR spectrum of **2** (black trace).

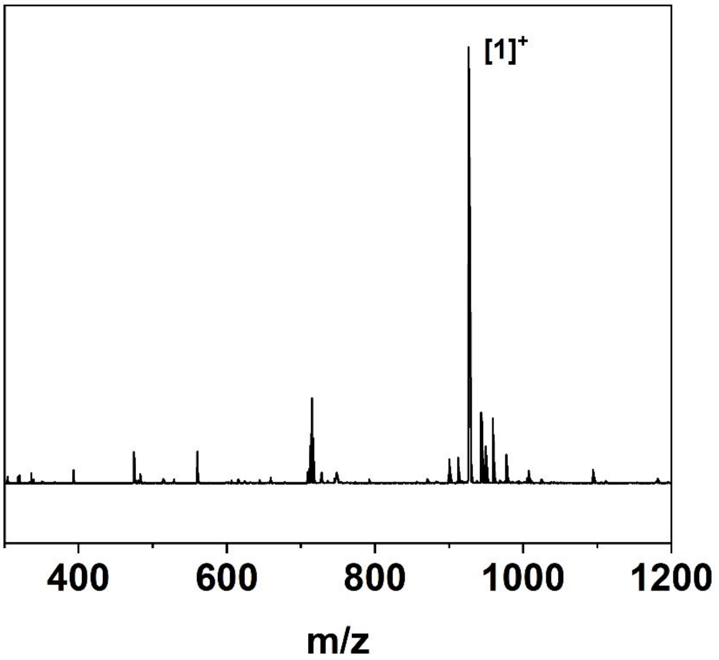

Figure S39. ESI-MS spectrum of $\mathbf{1}^{\bullet+}$ (generated with $[Ru(bpy)_3](PF_6)_3$) showing the dominant [**1**]$^+$ cation peak (*m/z* = 926.3906).

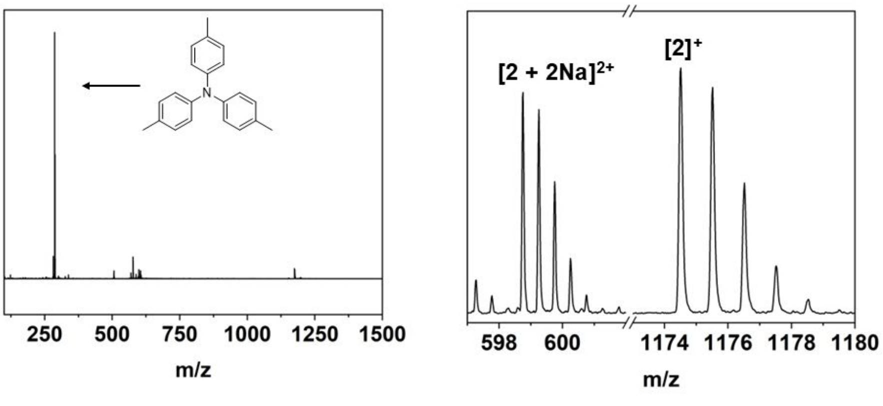

Figure S40. ESI-MS spectrum of $\mathbf{2}^{\bullet+}$ (generated with $[N(p\text{-tol})_3]SbCl_6$) showing the [**2**]$^+$ and [**2**+Na]$^{2+}$ cations (*m/z* = 1174.5156 and 598.7515).

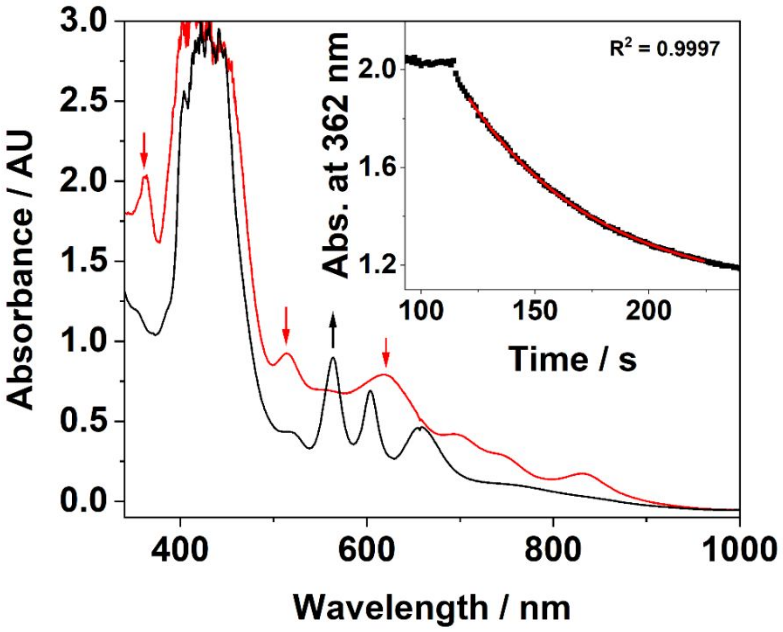

Figure S41. Changes to the electronic absorption spectrum of $1^{\bullet+}$ upon reaction with 4-CH_3O-2,6-DTBP (20 equiv.). Inset: Exponential fit of the decay of the absorption feature of $1^{\bullet+}$ at λ = 362 nm.

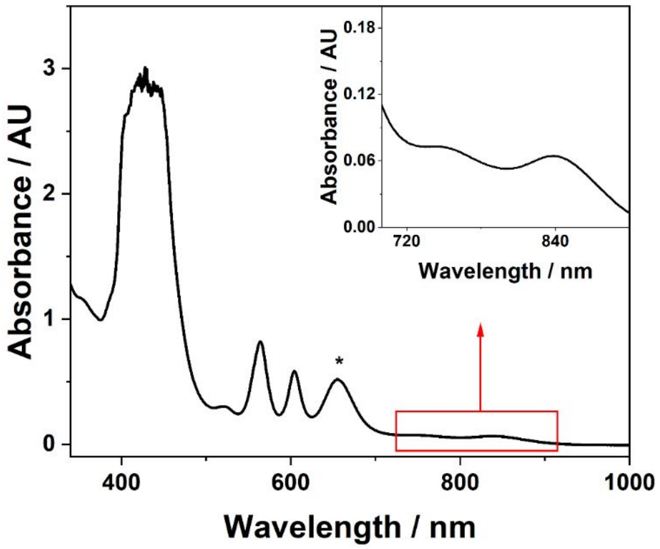

Figure S42. Post-reaction electronic absorption spectrum of the reaction between $2^{\bullet+}$ and 4-CH_3O-2,6-DTBP (20 equiv.) in CH_3CN at 20 °C, showing new bands at λ = 656 (marked by *) and λ = 750, 840 nm (inset).

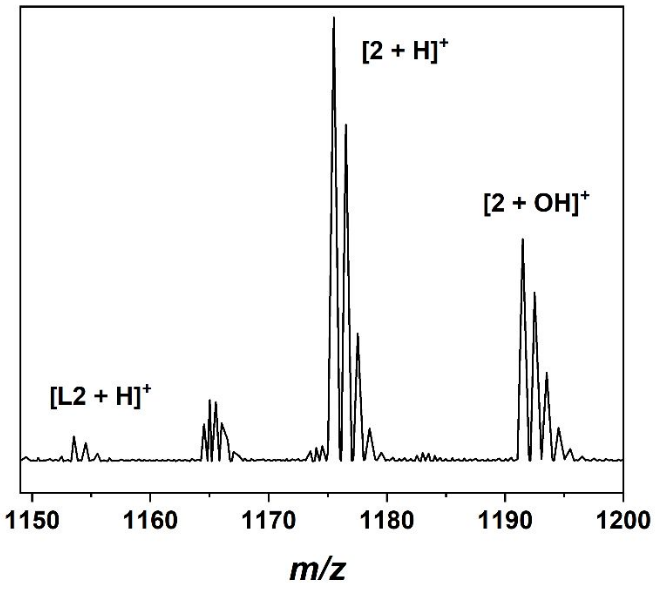

Figure S43. Post-reaction positive mode ESI-MS showing the [**L2** + H]$^+$ (m/z = 1153.5560, calculated m/z = 1153.5533), [**2** + H]$^+$ (m/z = 1175.5245, calculated m/z = 1175.5227) and [**2** + OH]$^+$ (m/z = 1191.5186, calculated m/z = 1191.5176) cations.

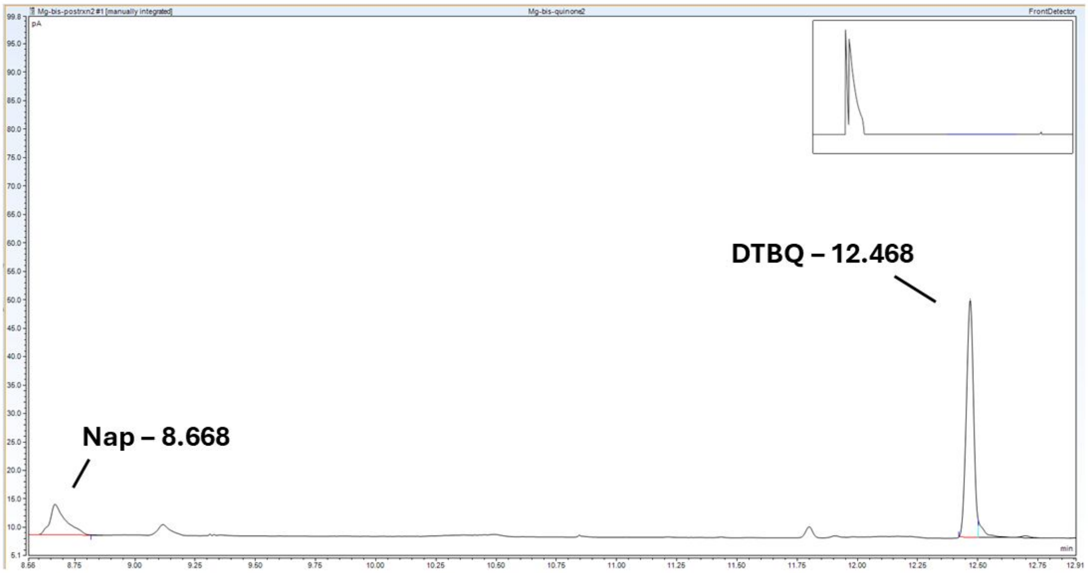

Figure S44. GC-FID chromatogram of the post-reaction mixture of the reaction between **2**$^{•+}$ and 4-CH_3O-2,6-DTBP, showing 2,6-di-*tert*-butylbenzoquinone (DTBQ), the naphthalene internal standard (Nap) and their respective retention times.

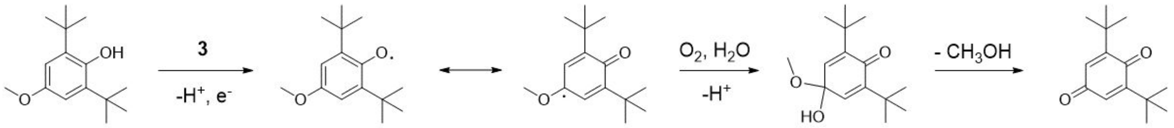

Scheme S2. Postulated mechanism of DTBQ formation following 1-electron oxidation of 4-CH_3O-2,6-DTBP by **2**$^{\bullet+}$.

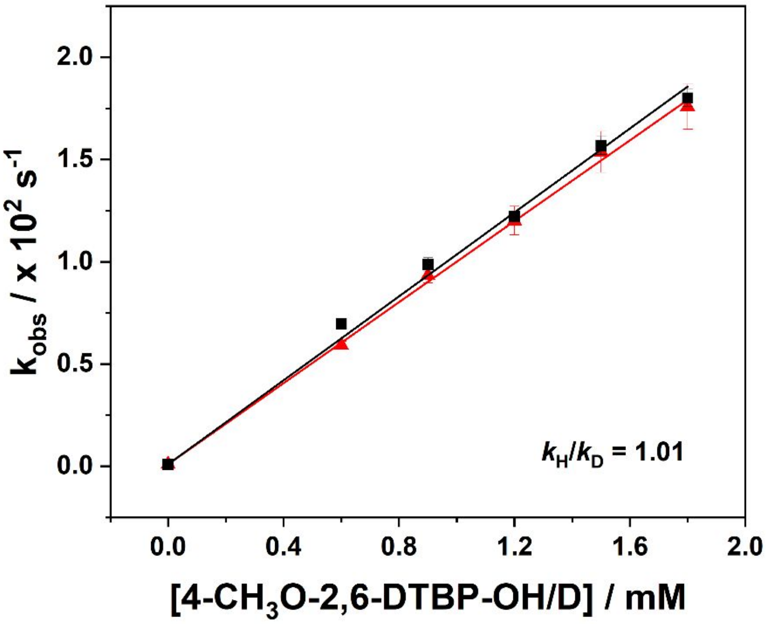

Figure S45. Plot of k_{obs} against substrate concentration for the reaction between **2**$^{\bullet+}$ and 4-CH_3O-2,6-DTBP (black) and 4-CH_3O-2,6-DTBP-OD (red).

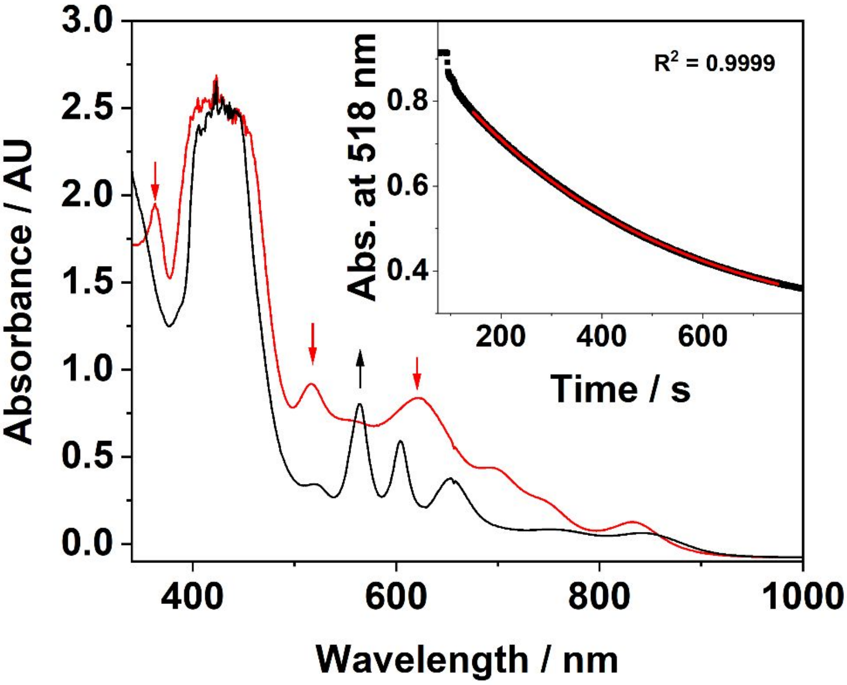

Figure S46. Changes to the electronic absorption spectrum of **2**$^{•+}$ upon reaction with 1,3-di-*tert*-butyl-2,5-dimethoxybenzene (120 equiv.). Inset: Exponential fit of the decay of the absorption feature of **2**$^{•+}$ at λ = 518 nm.

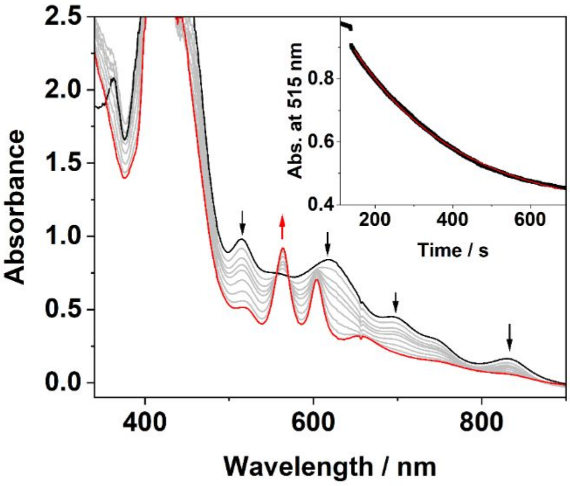

Figure S47. Changes to the electronic absorption spectrum of **1**$^{•+}$ upon reaction with 1,3-DTB-2,5-DMB (120 equiv.). Conditions: 60 μM **1**$^{•+}$, CH_3CN, 20 °C. Inset: Exponential fit of the decay of the absorption feature of **1**$^{•+}$ at λ = 515 nm.

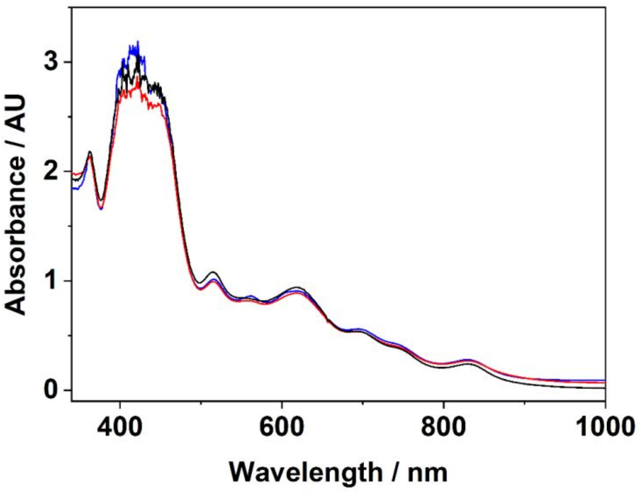

Figure S48. Comparison of the electronic absorption spectrum of $\mathbf{1}^{\bullet+}$ generated in the absence of cations (black trace), in the presence of $NaClO_4$ (10 equiv., red trace) and in the presence of $Mg(ClO_4)_2$ (10 equiv., blue trace).

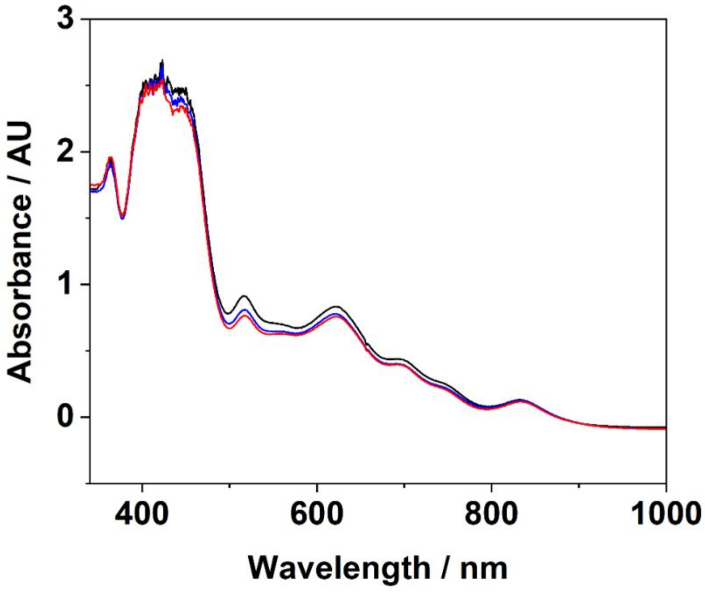

Figure S49. Electronic absorption spectrum of $\mathbf{2}^{\bullet+}$ generated in the absence of cations (black trace), in the presence of $NaClO_4$ (10 equiv., red trace) and in the presence of $Mg(ClO_4)_2$ (10 equiv., blue trace).

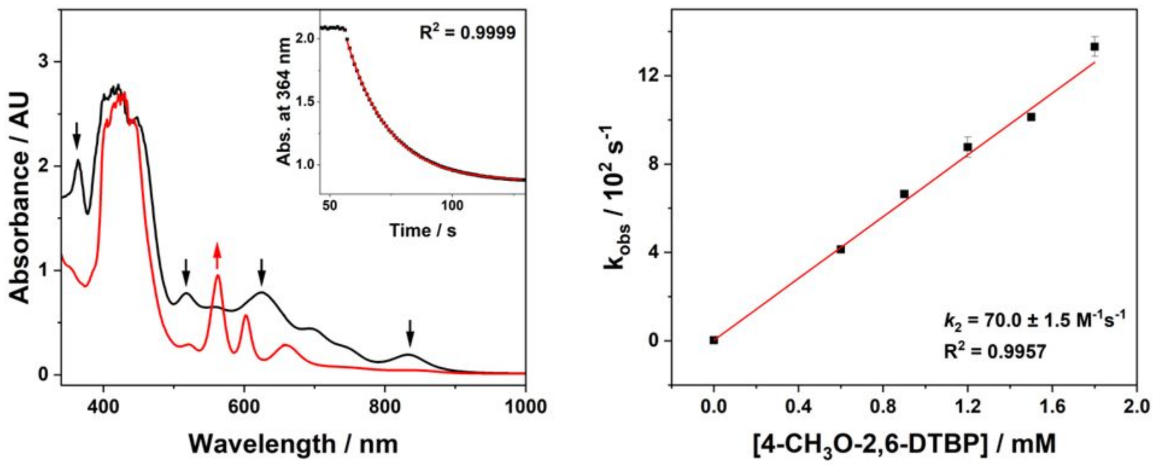

Figure S50. (Left) Changes to the electronic absorption spectrum of $\mathbf{2}^{\bullet+}$ upon reaction with 4-CH_3O-2,6-DTBP in the presence of $Mg(ClO_4)_2$ (10 equiv.). Inset: Exponential fit of the decay of the absorption feature of $\mathbf{2}^{\bullet+}$ at λ = 364 nm. (Right) Plot of k_{obs} against the concentration of substrate for the reaction of $\mathbf{2}^{\bullet+}$ with 4-CH_3O-2,6-DTBP in the presence of $Mg(ClO_4)_2$. The quoted error on k_2 refers to the standard error of the fit.

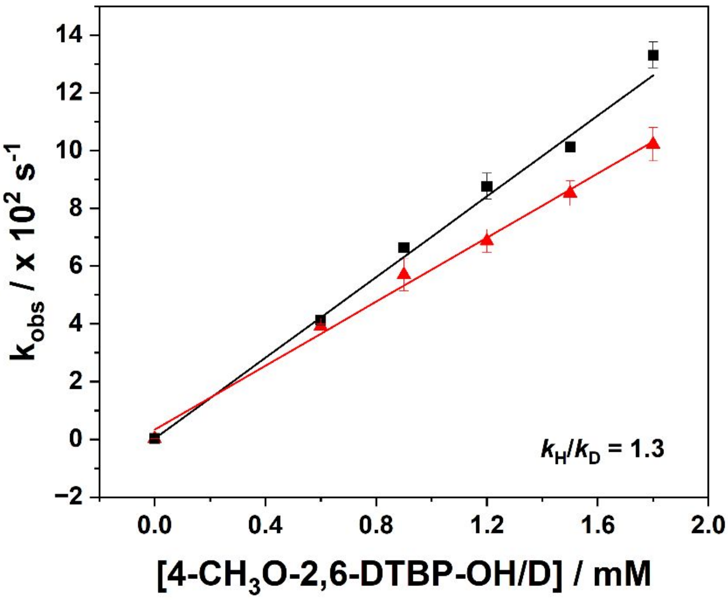

Figure S51. Plot of k_{obs} against substrate concentration for the reaction between $\mathbf{2}^{\bullet+}$ and 4-CH_3O-2,6-DTBP-OH (black) and 4-CH_3O-2,6-DTBP-OD (red) in the presence of $Mg(ClO_4)_2$ (10 equiv.)

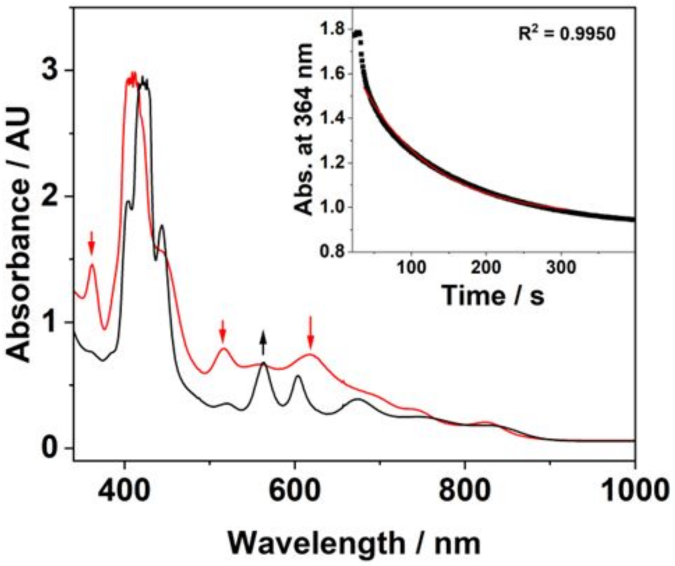

Figure S52. Reaction between [Mg(TTP$^{\bullet+}$)] (red trace) and 4-CH_3O-2,6-DTBP (10 equiv.). [Mg(TTP$^{\bullet+}$)] was generated using 1 equivalent of [N(*p*-tol)$_3$]$SbCl_6$ as oxidant. Conditions: 60 μM, CH_3CN, 20 °C.

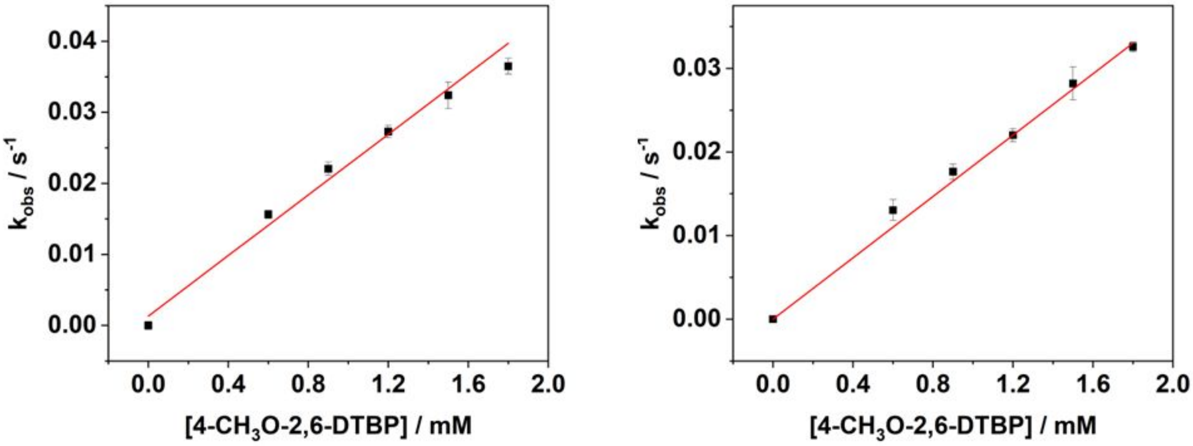

Figure S53. Plot of k_{obs} against the concentration of substrate for the reaction of [Mg(TTP$^{\bullet+}$)] with 4-CH_3O-2,6-DTBP in a) the absence of $Mg(ClO_4)_2$ (k_2 = 21.4 ± 1.4 $M^{-1}s^1$, R^2 = 0.9787), and b) the presence of $Mg(ClO_4)_2$ (k_2 = 18.3 ± 0.3 $M^{-1}s^1$, R^2 = 0.9986).

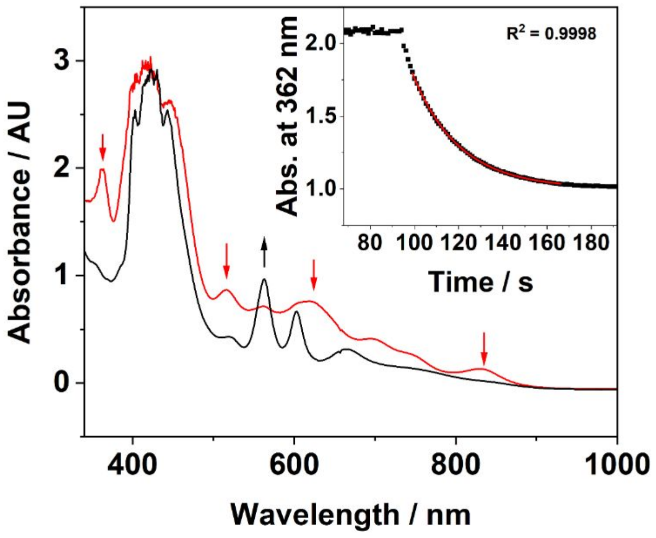

Figure S54. Changes to the electronic absorption spectrum of **1**$^{\bullet+}$ upon reaction with 4-CH_3O-2,6-DTBP (20 equiv.) in the presence of $Mg(ClO_4)_2$ (10 equiv.). Inset: Exponential fit of the decay of the absorption feature of **1**$^{\bullet+}$ at λ = 362 nm.

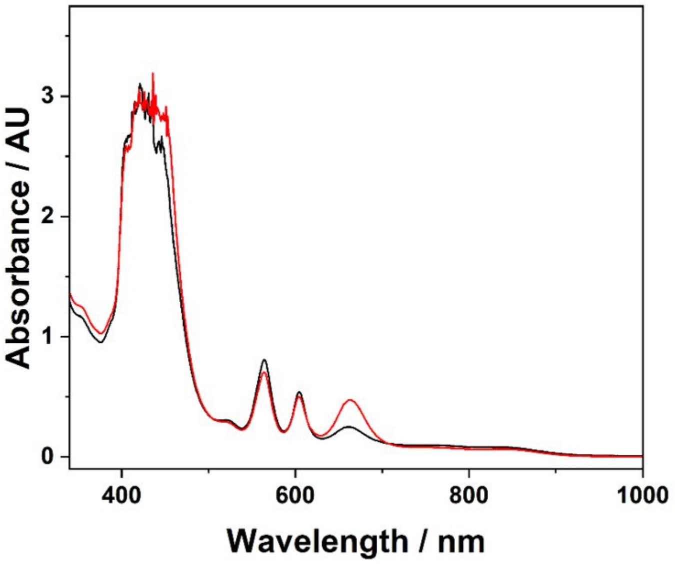

Figure S55. Comparison of the post reaction mixture electronic absorption spectra of **2**$^{\bullet+}$ with 4-CH_3O-2,6-DTBP in the absence of $NaClO_4$ (black trace) and the presence of 10 equivalents of $NaClO_4$ (red trace), showing the increased yield at λ = 656 nm in the presence of $NaClO_4$. Conditions: CH_3CN, 20 °C.

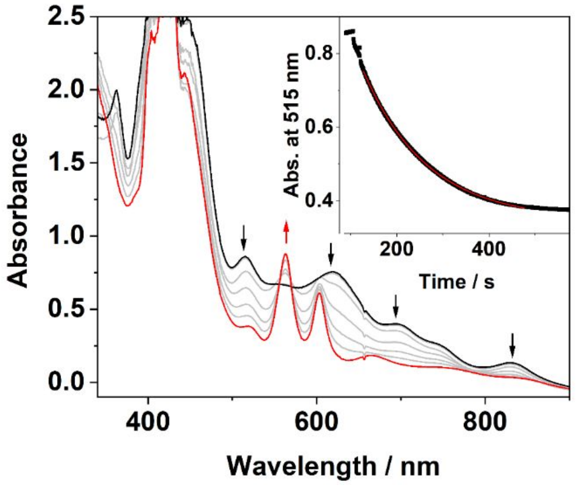

Figure S56. Changes to the electronic absorption spectrum of $\mathbf{1}^{\bullet+}$ upon reaction with 1,3-DTB-2,5-DMB (120 equiv.) in the presence of $NaClO_4$ (100 equiv.). Conditions: 60 µM $\mathbf{1}^{\bullet+}$, CH_3CN, 20 °C. Inset: Exponential fit of the decay of the absorption feature of $\mathbf{1}^{\bullet+}$ at λ = 515 nm.

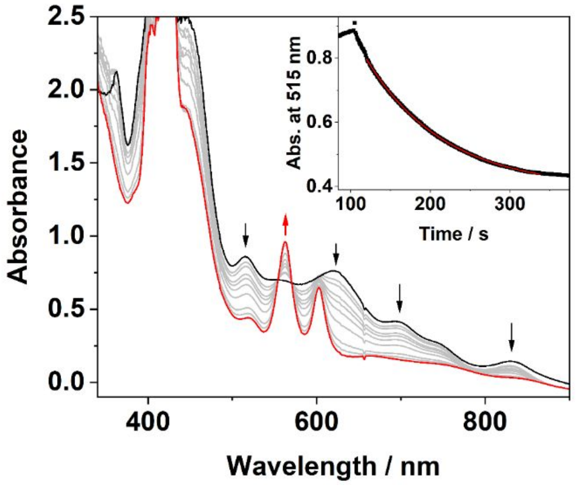

Figure S57. Changes to the electronic absorption spectrum of $\mathbf{1}^{\bullet+}$ upon reaction with 1,3-DTB-2,5-DMB (120 equiv.) in the presence of $Mg(ClO_4)_2$ (10 equiv.). Conditions: 60 µM $\mathbf{1}^{\bullet+}$, CH_3CN, 20 °C. Inset: Exponential fit of the decay of the absorption feature of $\mathbf{1}^{\bullet+}$ at λ = 515 nm.

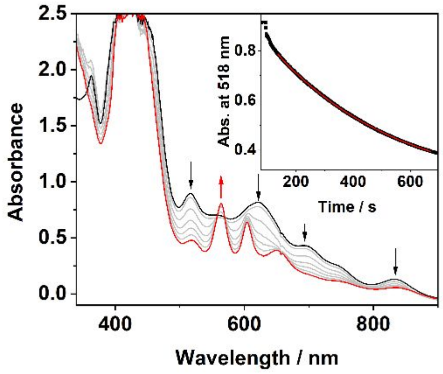

Figure S58. Changes to the electronic absorption spectrum of $\mathbf{2}^{\bullet+}$ upon reaction with 1,3-DTB-2,5-DMB (120 equiv.). Conditions: 60 μM $\mathbf{2}^{\bullet+}$, CH_3CN, 20 °C. Inset: Exponential fit of the decay of the absorption feature of $\mathbf{1}^{\bullet+}$ at λ = 518 nm.

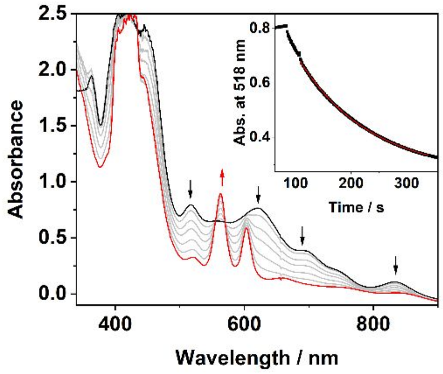

Figure S59. Changes to the electronic absorption spectrum of $\mathbf{2}^{\bullet+}$ upon reaction with 1,3-DTB-2,5-DMB (120 equiv.) in the presence of $NaClO_4$ (100 equiv.). Conditions: 60 μM $\mathbf{2}^{\bullet+}$, CH_3CN, 20 °C. Inset: Exponential fit of the decay of the absorption feature of $\mathbf{1}^{\bullet+}$ at λ = 518 nm.

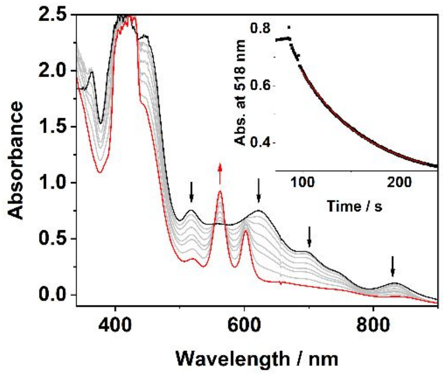

Figure S60. Changes to the electronic absorption spectrum of $2^{•+}$ upon reaction with 1,3-DTB-2,5-DMB (120 equiv.) in the presence of $Mg(ClO_4)_2$ (10 equiv.). Conditions: 60 μM $2^{•+}$, CH_3CN, 20 °C. Inset: Exponential fit of the decay of the absorption feature of $1^{•+}$ at λ = 518 nm.

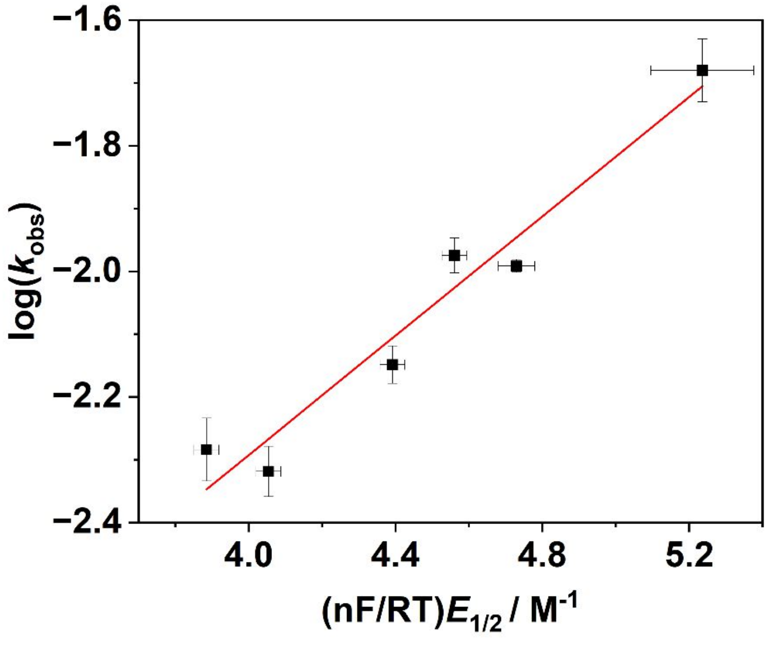

Figure S61. Plot of log(k_{obs}) against $E_{1/2}$ for the reactions of $1^{•+}$, $2^{•+}$ (and their Na^+ and Mg^{2+} adducts) with 1,3-DTB-2,5-DMB (120 equiv.) in CH_3CN at 20 °C.

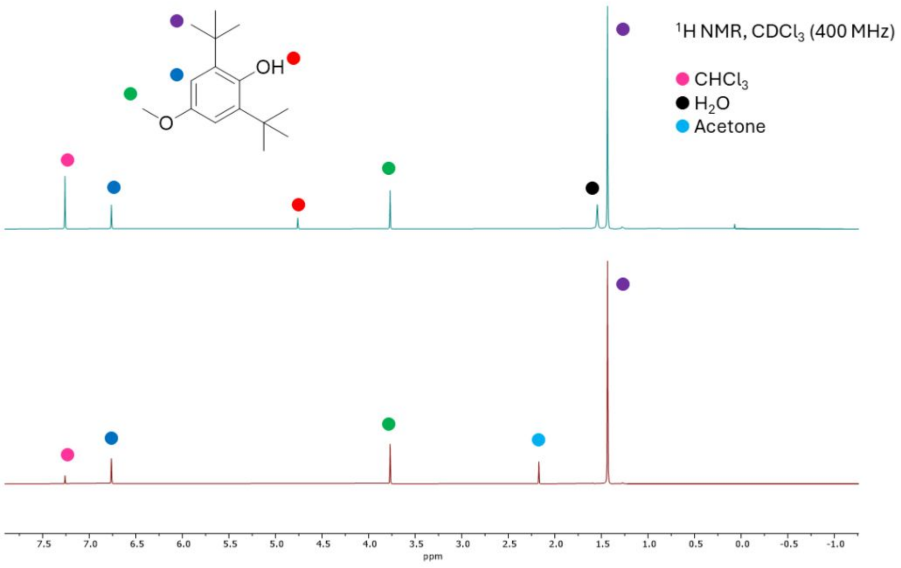

Figure S62. ^{1}H NMR spectra of 4-CH_3O-2,6-DTBP-OH (blue) and 4-CH_3O-2,6-DTBP-OD (red) showing the absence of the O*H* resonance in the latter.

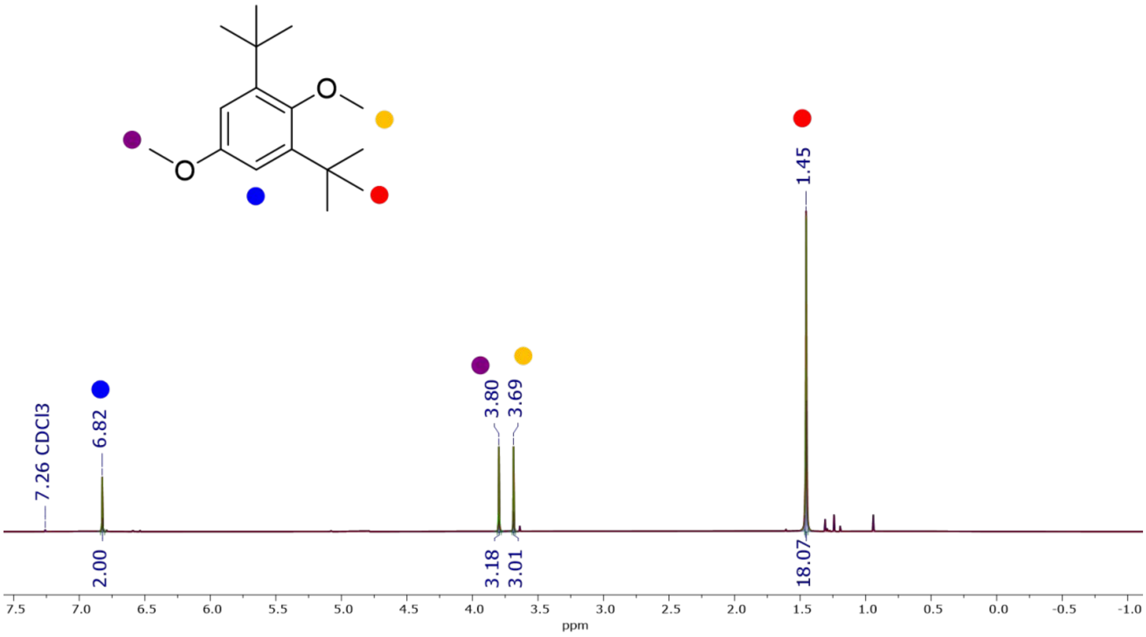

Figure S63. ^{1}H NMR spectrum (400 MHz, $CDCl_3$) of 2,6-di-*tert*-butyl-1,4-dimethoxybenzene.

Table S1. Selected bond lengths (Å) for complexes **1** and **1(THF)$_2$.Na** determined by SC-XRD.

	**1**	**1(THF)$_2$.Na**
Mg–N	2.076(5)	2.071(2)
	2.084(5)	2.070(2)
	2.081(5)	2.068(2)
	2.080(5)	2.077(2)
Mg–O (axial)*	2.103(4)	2.1854(19)
	2.106(5)	2.2901(19)
Na–O (crown)	-	2.441(5)
	-	2.458(5)
	-	2.408(4)
	-	2.389(4)
	-	2.463(4)
Na–O (perchlorate)	-	2.473(5)
	-	2.501(5)

* H_2O for **1**, THF for **1(THF)$_2$.Na**

Table S2. Crystal data and structure refinement for **1** and **1(THF)$_2$.Na**.

	**1**	**1(THF)$_2$.Na**
**CCDC number**	2390925	2390926
**Empirical formula**	$C_{63}H_{70}MgN_4O_8$	$C_{70}H_{78}ClMgN_4NaO_{13}$
**Formula weight**	1035.54	1266.11
**Temperature [K]**	100(2)	100(2)
**Crystal system**	triclinic	monoclinic
**Space group (number)**	$P\bar{1}$ (2)	$P2_1/c$ (14)
a **[Å]**	8.1632(4)	27.6715(8)
b **[Å]**	16.0211(9)	9.4496(3)
c **[Å]**	23.4596(12)	24.3694(7)
**α [°]**	70.752(5)	90
**β [°]**	79.984(3)	92.3961(18)
**γ [°]**	75.490(4)	90
**Volume [Å^3]**	2790.2(3)	6366.7(3)
Z	2	4
**ρ_{calc} [gcm^{-3}]**	1.233	1.321
**μ [mm^{-1}]**	0.749	1.255
***F*(000)**	1104	2680
**Crystal size [mm^3]**	0.173×0.08×0.065	0.821×0.057×0.052
**Crystal color**	blue	red
**Crystal shape**	block	needle
**Radiation**	Cu K_α (λ=1.54178 Å)	Cu K_α (λ=1.54178 Å)
**2θ range [°]**	4.01 to 136.86 (0.83 Å)	3.20 to 136.83 (0.83 Å)
**Index ranges**	$-9 \le h \le 9$<br>$-19 \le k \le 17$<br>$-28 \le l \le 27$	$-33 \le h \le 33$<br>$-11 \le k \le 10$<br>$-29 \le l \le 28$
**Reflections collected**	37236	94617
**Independent reflections**	10199<br>R_{int} = 0.0764<br>R_{sigma} = 0.0733	11663<br>R_{int} = 0.0868<br>R_{sigma} = 0.0511
**Completeness to θ = 67.679°**	99.8 %	100.0 %
**Data / Restraints / Parameters**	10199/1072/900	11663/631/1005
**Absorption correction T_{min}/T_{max} (method)**	0.6327/0.7531 (multi-scan)	0.5327/0.7531 (multi-scan)
**Goodness-of-fit on F^2**	1.059	1.034
**Final *R* indexes [$I \geq 2\sigma(I)$]**	R_1 = 0.1297<br>wR_2 = 0.3251	R_1 = 0.0596<br>wR_2 = 0.1595
**Final *R* indexes [all data]**	R_1 = 0.1673<br>wR_2 = 0.3475	R_1 = 0.0808<br>wR_2 = 0.1757
**Largest peak/hole [eÅ^{-3}]**	0.55/−0.42	0.54/−0.42

Table S3. Maximum shifts in $E_{1/2}$ observed for the addition of $NaClO_4$ and $Mg(ClO_4)_2$ to $[Mg(H_2O)(TTP)]$, **1**, and **2**. Errors are given as the standard deviation of three measurements.

	**Max. $\Delta E_{1/2}$ (mV)**	
**Compound**	**Additive**	
	**$NaClO_4$**	**$Mg(ClO_4)_2$**
$[Mg(H_2O)(TTP)]$	0 ± 2	0 ± 2
**1**	17 ± 2	43 ± 3
**2**	38 ± 2	84 ± 5

Table S4. Calculated frontier MO energies (eV, DCM; B97-3c).

Species	HOMO	LUMO	$\Delta E_{HOMO-LUMO}$
**[Mg(TTP)]**	-4.394	-2.718	1.675
**1**	-4.447	-2.719	1.727
**1.Na**	-4.464	-2.748	1.716
**1.Mg**	-4.487	-2.766	1.720
**2**	-4.429	-2.711	1.719
**2.Na**	-4.501	-2.791	1.710
**2.Mg**	-4.470	-2.754	1.716

References

(1) Krause, L.; Herbst-Irmer, R.; Sheldrick, G. M.; Stalke, D. Comparison of silver and molybdenum microfocus X-ray sources for single-crystal structure determination. *J. Appl. Crystallogr.* **2015**, *48* (1), 3-10.

(2) *Bruker, SAINT*; Bruker AXS Inc.: Madison, Wisconsin, USA, 2016.

(3) Dolomanov, O. V.; Bourhis, L. J.; Gildea, R. J.; Howard, J. A. K.; Puschmann, H. OLEX2: a complete structure solution, refinement and analysis program. *J. Appl. Crystallogr.* **2009**, *42* (2), 339-341.

(4) Sheldrick, G. SHELXT - Integrated space-group and crystal-structure determination. *Acta Cryst. A* **2015**, *71* (1), 3-8.

(5) Sheldrick, G. Crystal structure refinement with SHELXL. *Acta Cryst. C* **2015**, *71* (1), 3-8.

(6) Groom, C. R.; Bruno, I. J.; Lightfoot, M. P.; Ward, S. C. The Cambridge Structural Database. *Acta Cryst. B* **2016**, *72* (2), 171-179.

(7) *FinalCif*; D. Kratzert: https://dkratzert.de/finalcif.html.

(8) Stoll, S.; Schweiger, A. EasySpin, a comprehensive software package for spectral simulation and analysis in EPR. *J. Magn. Reson.* **2006**, *178* (1), 42-55.

(9) Brandenburg, J. G.; Bannwarth, C.; Hansen, A.; Grimme, S. B97-3c: A revised low-cost variant of the B97-D density functional method. *J. Chem. Phys.* **2018**, *148* (6), 064104.

(10) Neese, F.; Wennmohs, F.; Becker, U.; Riplinger, C. The ORCA quantum chemistry program package. *J. Chem. Phys.* **2020**, *152* (22), 224108.

(11) Neese, F. Software update: The ORCA program system—Version 5.0. *WIREs Computational Molecular Science* **2022**, *12* (5), e1606.

(12) Dishong, D. M.; Diamond, C. J.; Cinoman, M. I.; Gokel, G. W. Crown cation complex effects. 20. Syntheses and cation binding properties of carbon-pivot lariat ethers. *J. Am. Chem. Soc.* **1983**, *105* (3), 586-593.

(13) Kuś, P. Tetraphenylporphyrins monosubstituted with a crown ether in one phenyl ring. Synthesis and characterization. *Monatsh. Chem.* **1997**, *128* (8), 911-917.

(14) Gust, D.; Moore, T. A.; Bensasson, R. V.; Mathis, P.; Land, E. J.; Chachaty, C.; Moore, A. L.; Liddell, P. A.; Nemeth, G. A. Stereodynamics of intramolecular triplet energy transfer in carotenoporphyrins. *J. Am. Chem. Soc.* **1985**, *107* (12), 3631-3640.

(15) Iida, K.; Yoshida, J.-i. Radical Polymerization Initiated by Electron Transfer Driven C−Si Bond Dissociation. *Macromolecules* **2006**, *39* (19), 6420-6424.

(16) Hidalgo-Acosta, J. C.; Méndez, M. A.; Scanlon, M. D.; Vrubel, H.; Amstutz, V.; Adamiak, W.; Opallo, M.; Girault, H. H. Catalysis of water oxidation in acetonitrile by iridium oxide nanoparticles. *Chem. Sci.* **2015**, *6* (3), 1761-1769.

(17) Lee, J. Y.; Peterson, R. L.; Ohkubo, K.; Garcia-Bosch, I.; Himes, R. A.; Woertink, J.; Moore, C. D.; Solomon, E. I.; Fukuzumi, S.; Karlin, K. D. Mechanistic Insights into the Oxidation of Substituted Phenols via Hydrogen Atom Abstraction by a Cupric–Superoxo Complex. *J. Am. Chem. Soc.* **2014**, *136* (28), 9925-9937.

(18) Connelly, N. G.; Geiger, W. E. Chemical Redox Agents for Organometallic Chemistry. *Chem. Rev.* **1996**, *96* (2), 877-910.

(19) Lindsey, J. S.; Woodford, J. N. A Simple Method for Preparing Magnesium Porphyrins. *Inorg. Chem.* **1995**, *34* (5), 1063-1069.

(20) Hess, K. M.; Leach, I. F.; Wijtenhorst, L.; Lee, H.; Klein, J. E. M. N. Valence Tautomerism Induced Proton Coupled Electron Transfer:X−H Bond Oxidation with a Dinuclear Au(II) Hydroxide Complex. *Angew. Chem. Int. Ed.* **2024**, *63* (14), e202318916.

(21) Rathore, R.; Bosch, E.; Kochi, J. K. Selective nitration versus oxidative dealkylation of hydroquinone ethers with nitrogen dioxide. *Tetrahedron* **1994**, *50* (23), 6727-6758.

(22) Thordarson, P. Determining association constants from titration experiments in supramolecular chemistry. *Chem. Soc. Rev.* **2011**, *40* (3), 1305-1323.
